# Supplementary figures and images for: Loss of Ecrg4 improves calcium oxalate nephropathy
Source: PLoS One. 2022 Oct 13;17(10):e0275972. doi: 10.1371/journal.pone.0275972 (PMC9560046; doi:10.1371/journal.pone.0275972)

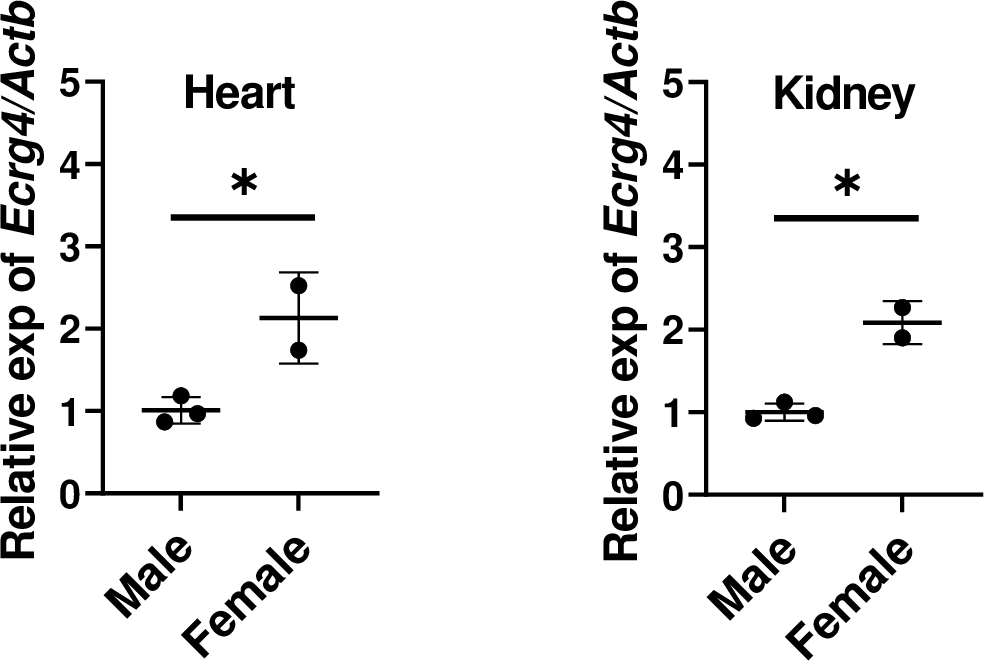

Supplement: S1 Fig — Ecrg4 mRNA expression in the indicated organs relative to Actb (n = 2–3). Data are shown as individual dots and mean ± SD. Stars indicate *p<0.05, calculated using Student t-test. (TIF) [file pone.0275972.s001.tif]

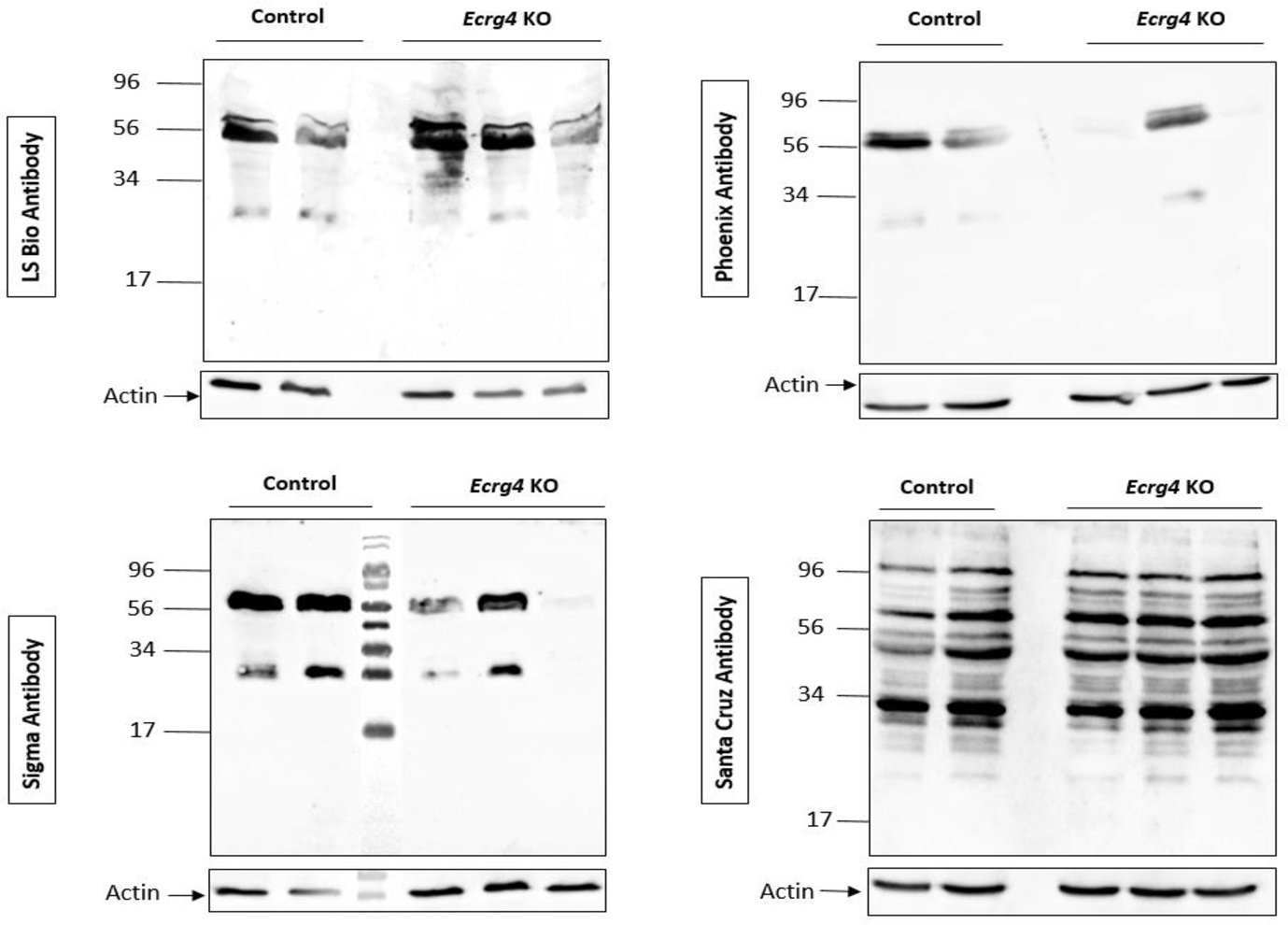

Supplement: S2 Fig — The equivalent of 50ug of protein was loaded on 13% SDS gel. The LS-Bio antibody (diluted 1:500, #LS-C172856), the Phoenix antibody (diluted 1:300, #012–25), the Sigma antibody (diluted 1:500, #HPA008546) and the Santa Cruz antibody (diluted 1:500, # H-118) were used. Actin (diluted 1:500, #A2066) was used as loading control. (TIF) [file pone.0275972.s002.tif]

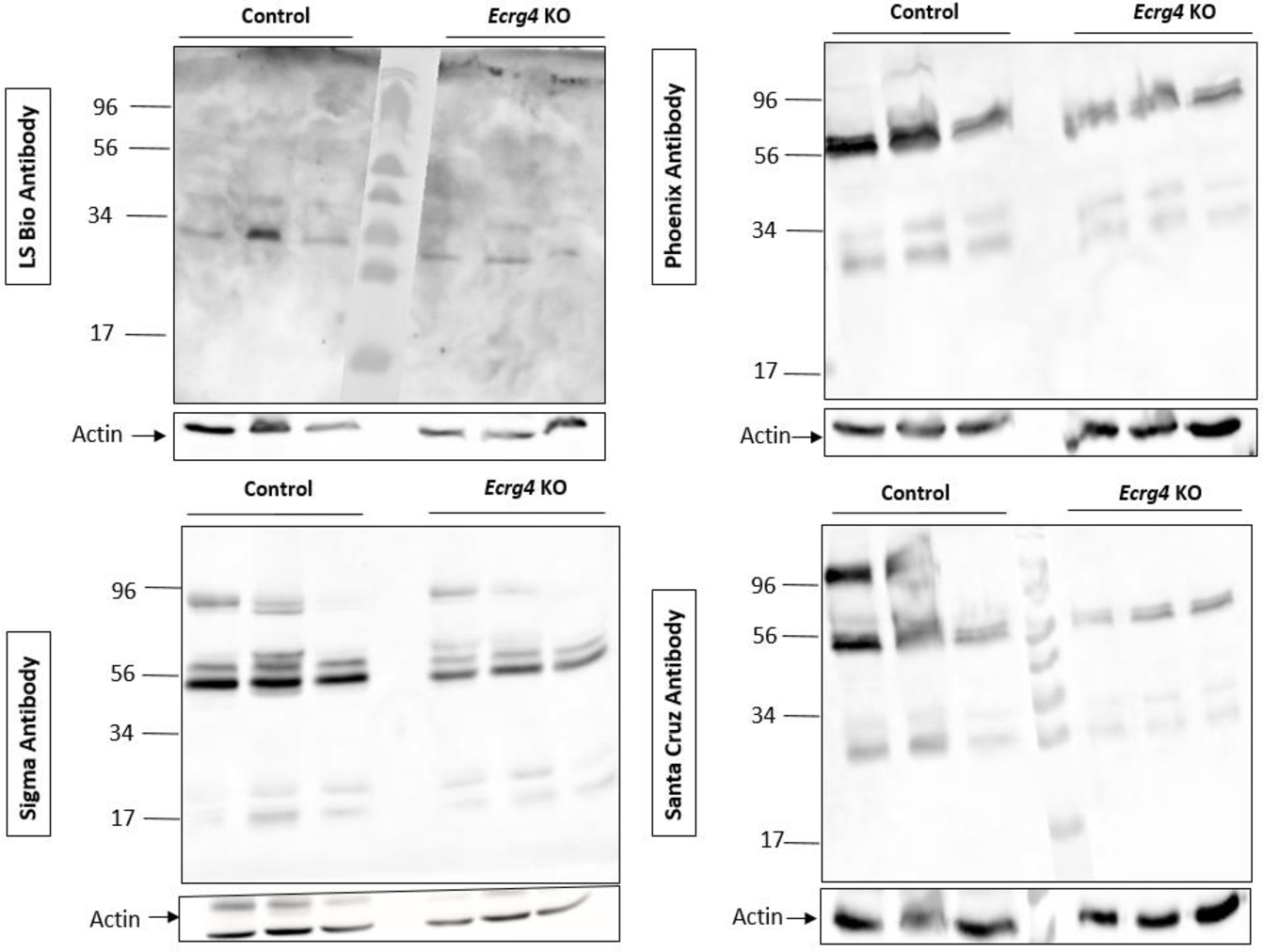

Supplement: S3 Fig — The equivalent of 50ug of protein was loaded on 13% SDS gel. The LS-Bio antibody (diluted 1:500, #LS-C172856), the Phoenix antibody (diluted 1:300, #012–25), the Sigma antibody (diluted 1:500, #HPA008546) and the Santa Cruz antibody (diluted 1:500, # H-118) were used. Actin (diluted 1:500, #A2066) was used as loading control. (TIF) [file pone.0275972.s003.tif]

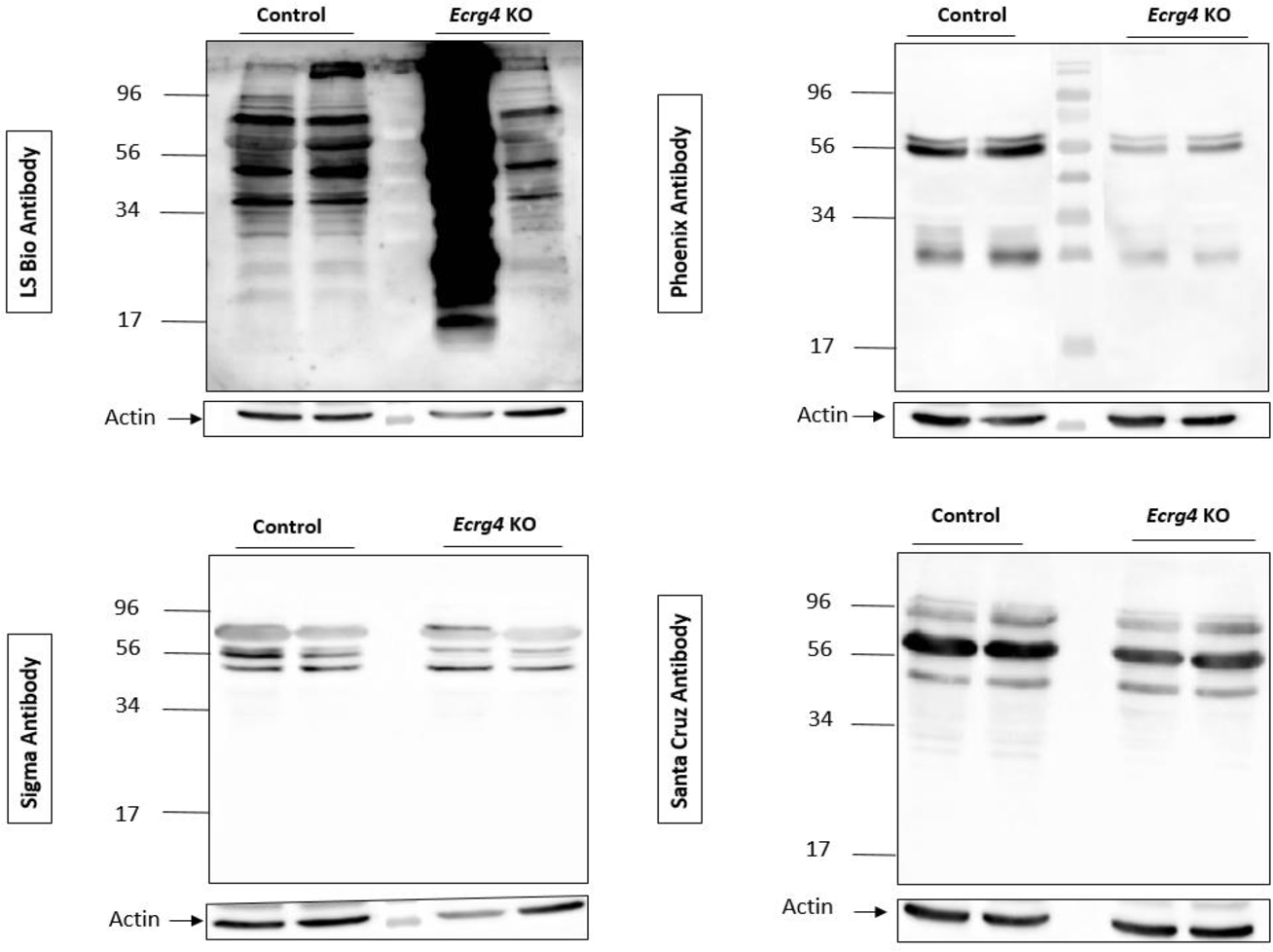

Supplement: S4 Fig — 100ug of protein was loaded on 13% SDS gel. The LS-Bio antibody (diluted 1:500, #LS-C172856), the Phoenix antibody (diluted 1:300, #012–25), the Sigma antibody (diluted 1:500, #HPA008546) and the Santa Cruz antibody (diluted 1:500, # H-118) were used. Actin (diluted 1:500, #A2066) was used as loading control. (TIF) [file pone.0275972.s004.tif]

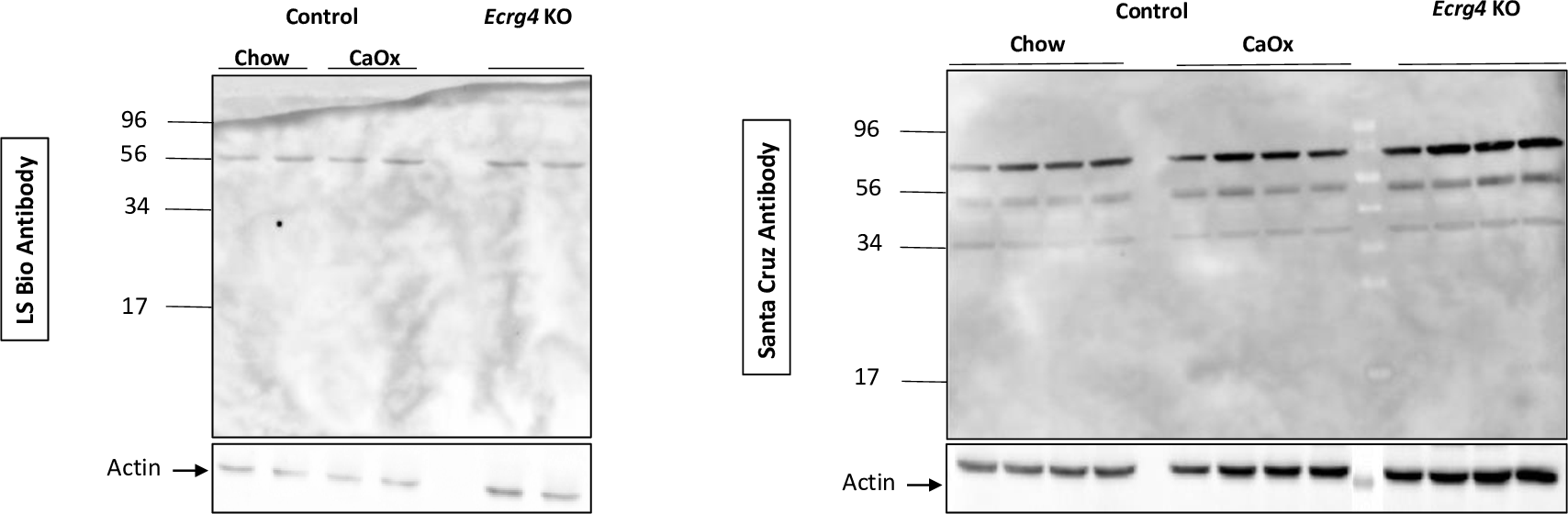

Supplement: S5 Fig — 30ug of protein was loaded on 13% SDS gel. The LS-Bio antibody (diluted 1:500, #LS-C172856), and the Santa Cruz antibody (diluted 1:500, # H-118) were used. Actin (diluted 1:500, #A2066) was used as loading control. (TIF) [file pone.0275972.s005.tif]

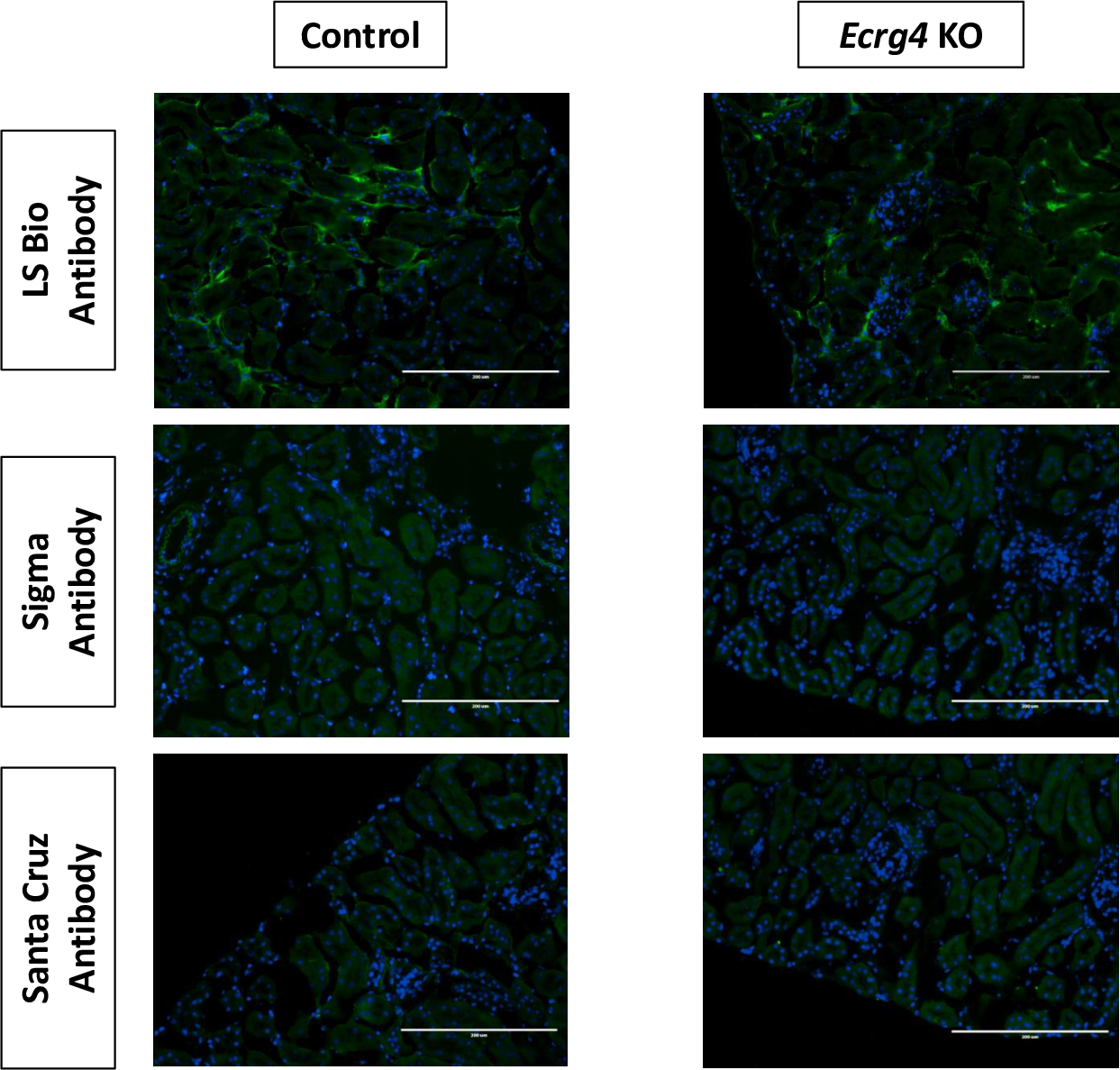

Supplement: S6 Fig — Scale bars represent 200um. The LS-Bio antibody (diluted 1:100, #LS-C172856), the Sigma antibody (diluted 1:100, #HPA008546) and the Santa Cruz antibody (diluted 1:100, # H-118) were used. (TIF) [file pone.0275972.s006.tif]

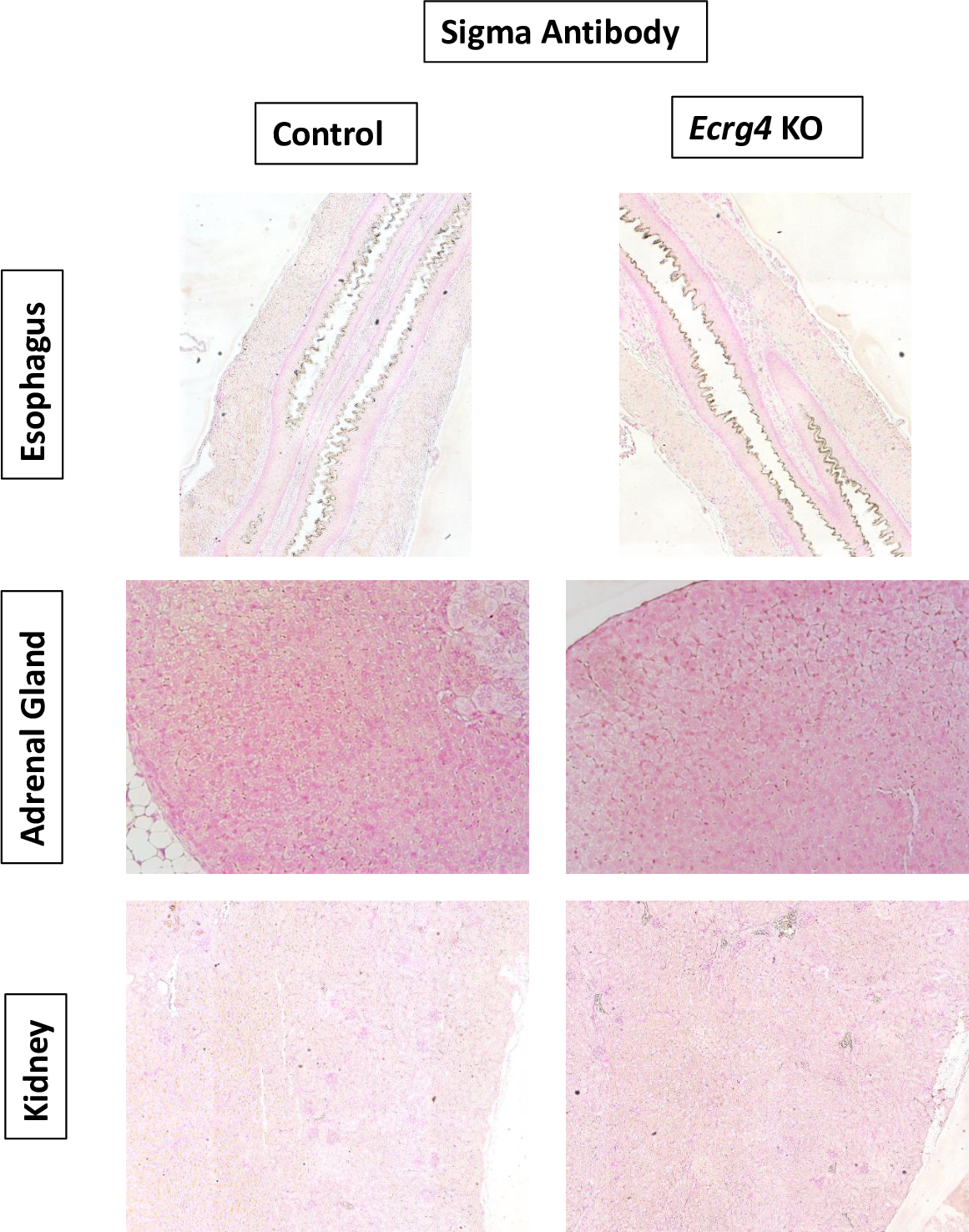

Supplement: S7 Fig — The Sigma antibody (diluted 1:50, #HPA008546) was used. (TIF) [file pone.0275972.s007.tif]

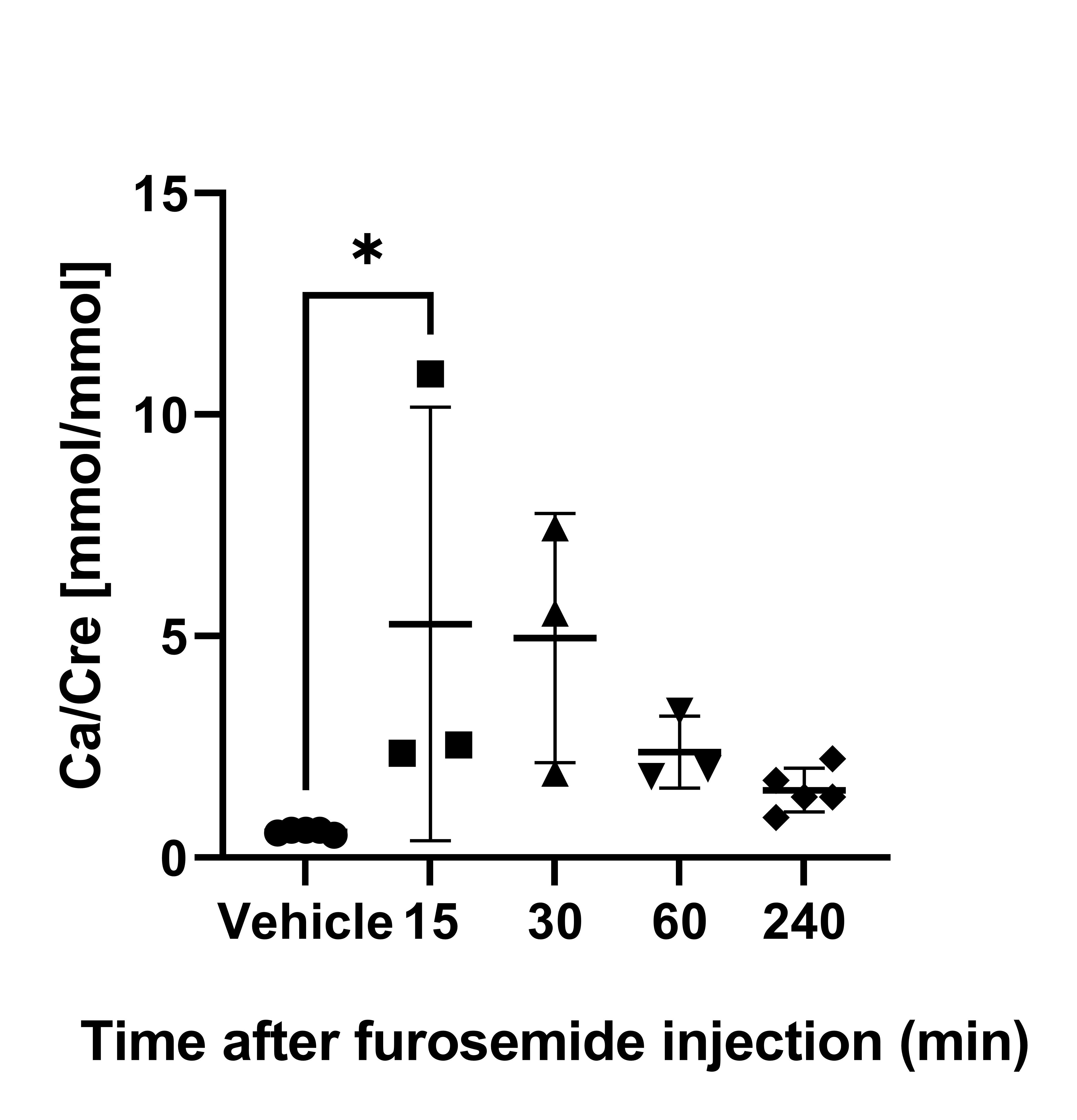

Supplement: S8 Fig — Mice were injected with 20mg/kg body weight furosemide or vehicle, and sacrificed after 15, 30, 60 and 240 min. P values are calculated using one-way ANOVA test, with Dunnett correction for multiple comparison. Each time point was compared to the vehicle. Data are shown as mean ± SD. Stars indicate * p<0.05. (TIF) [file pone.0275972.s008.tif]

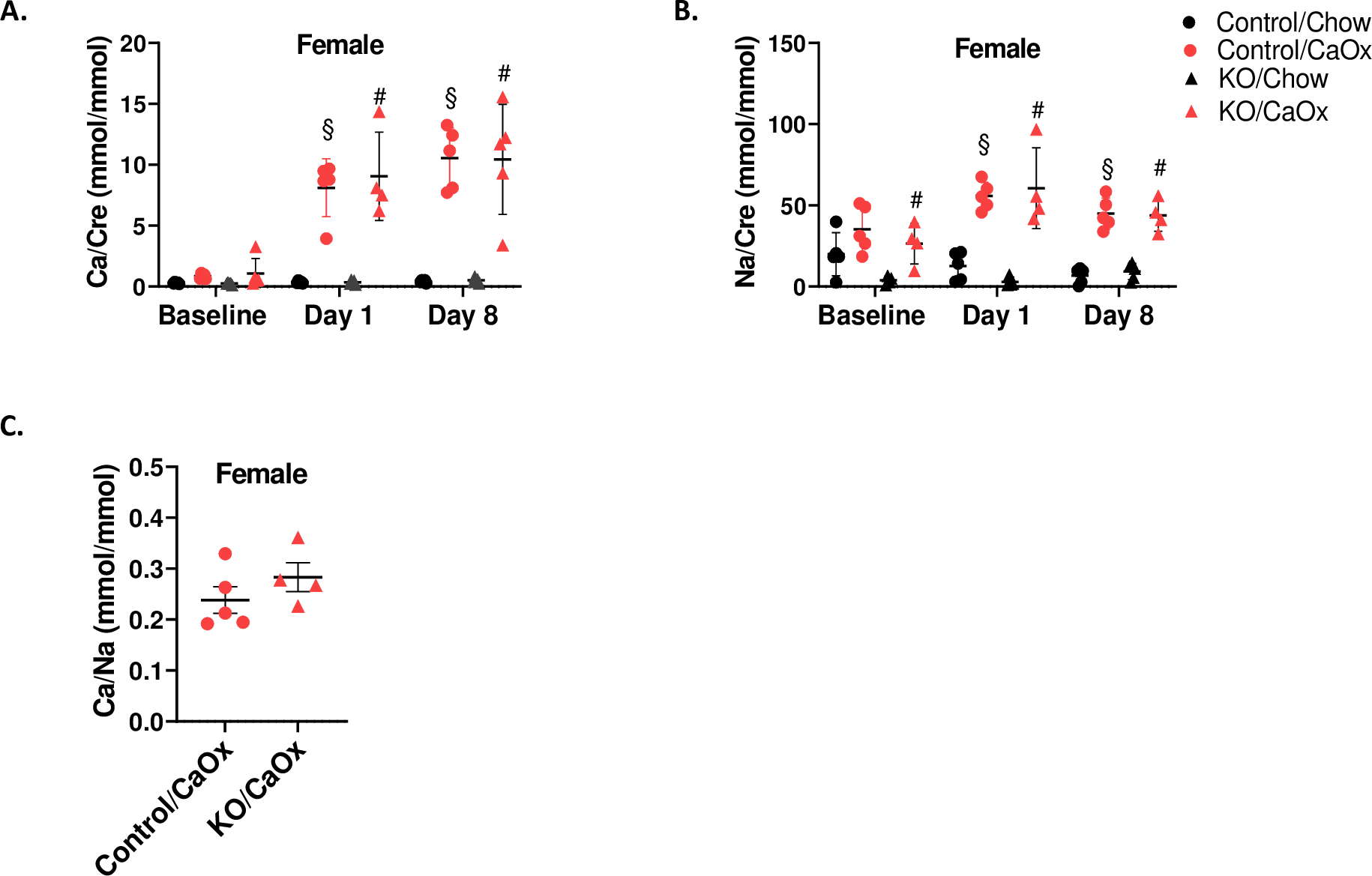

Supplement: S9 Fig — (A) Increase in the urinary calcium-creatinine ratio in both control and Ecrg4 KO female mice upon CaOx diet. The ratios were calculated from the urine collected at baseline, and after 1 day and 8 days of exposure to either chow diet or the CaOx diet. (B) Urinary sodium-creatinine ratio in both control and Ecrg4 KO female mice. (C) Urinary calcium/sodium excretion at day 8 of the CaOx diet. Data are shown as mean ± SD. p value was calculated using two-way ANOVA with Tukey correction for multiple comparison. Stars indicate * p<0.05 between the control and Ecrg4 KO, § indicates p<0.05 between the controls of different diets, # indicates p<0.05 between the Ecrg4 KO of different diets. (TIF) [file pone.0275972.s009.tif]

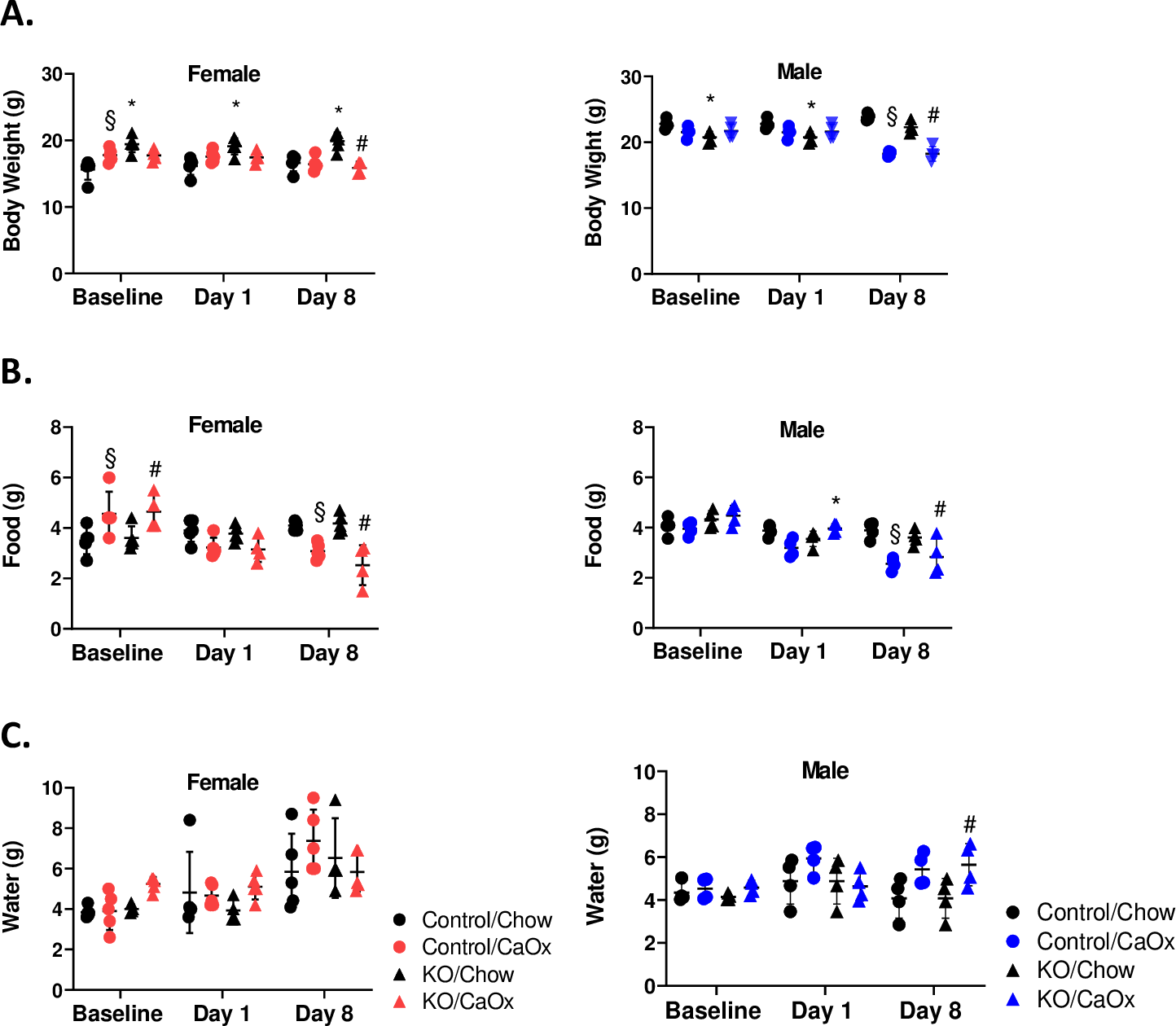

Supplement: S10 Fig — (A) Body weight, (B) food and (C) water intake parameters were measured over 24h period for males and females. At baseline, all mice were fed the chow diet and then exposed to either CaOx diet or chow diet (n = 4–5). Data are shown as mean ± SD. p value was calculated using two-way ANOVA with Tukey correction for multiple comparison. Stars indicate * p<0.05 between the control and Ecrg4 KO, § indicates p<0.05 between the controls of different diets, # indicates p<0.05 between the Ecrg4 KO of different diets. (TIF) [file pone.0275972.s010.tif]

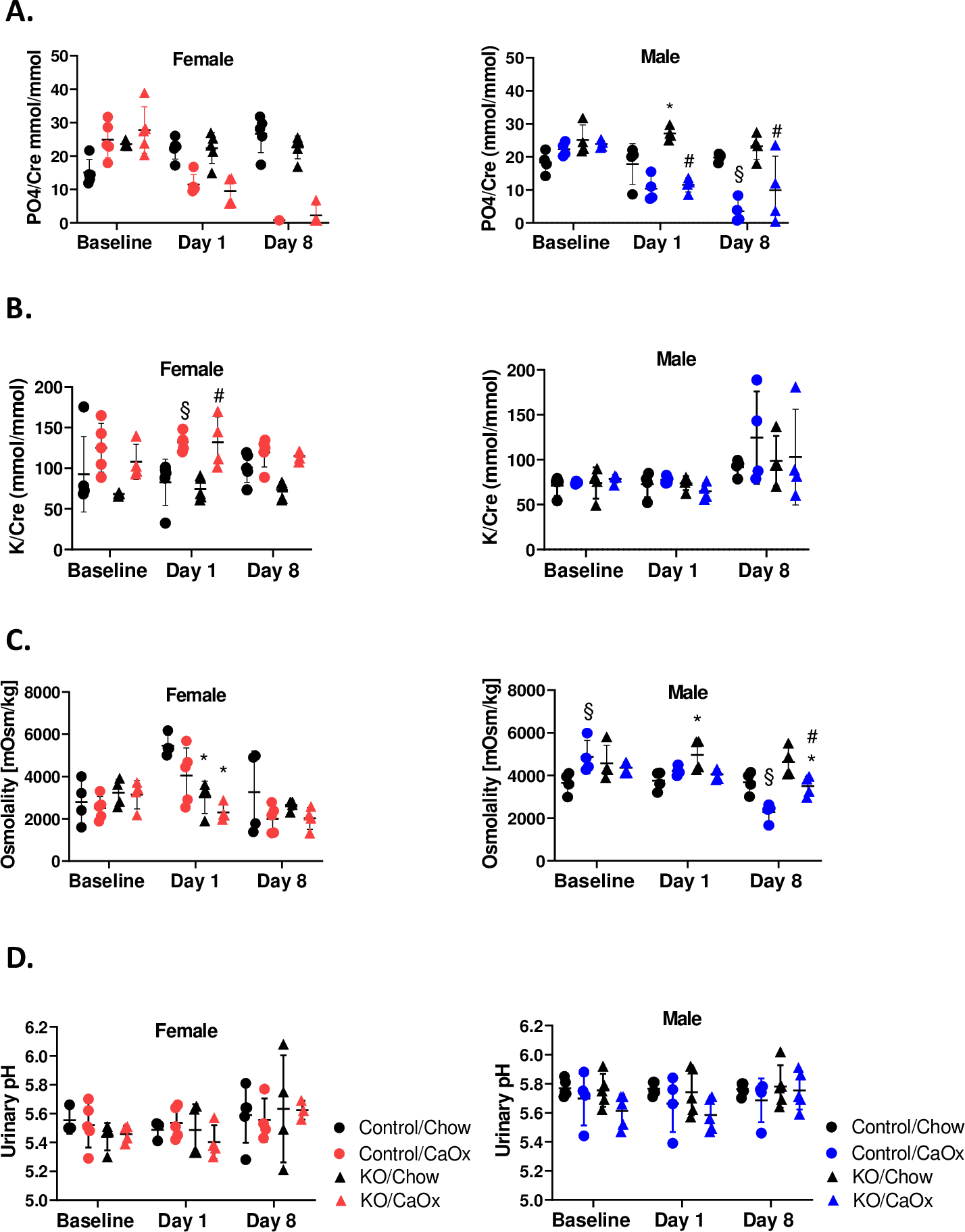

Supplement: S11 Fig — Urinary phosphate (A), potassium (B) excretion, osmolality (C) and pH (D). Mice were individually housed in metabolic cages, after exposure to chow or CaOx diet (n = 4–5). Data are shown as mean ± SD. p value was calculated using two-way ANOVA with Tukey correction for multiple comparison. Stars indicate * p<0.05 between the control and Ecrg4 KO, § indicates p<0.05 between the controls of different diets, # indicates p<0.05 between the Ecrg4 KO of different diets. (TIF) [file pone.0275972.s011.tif]

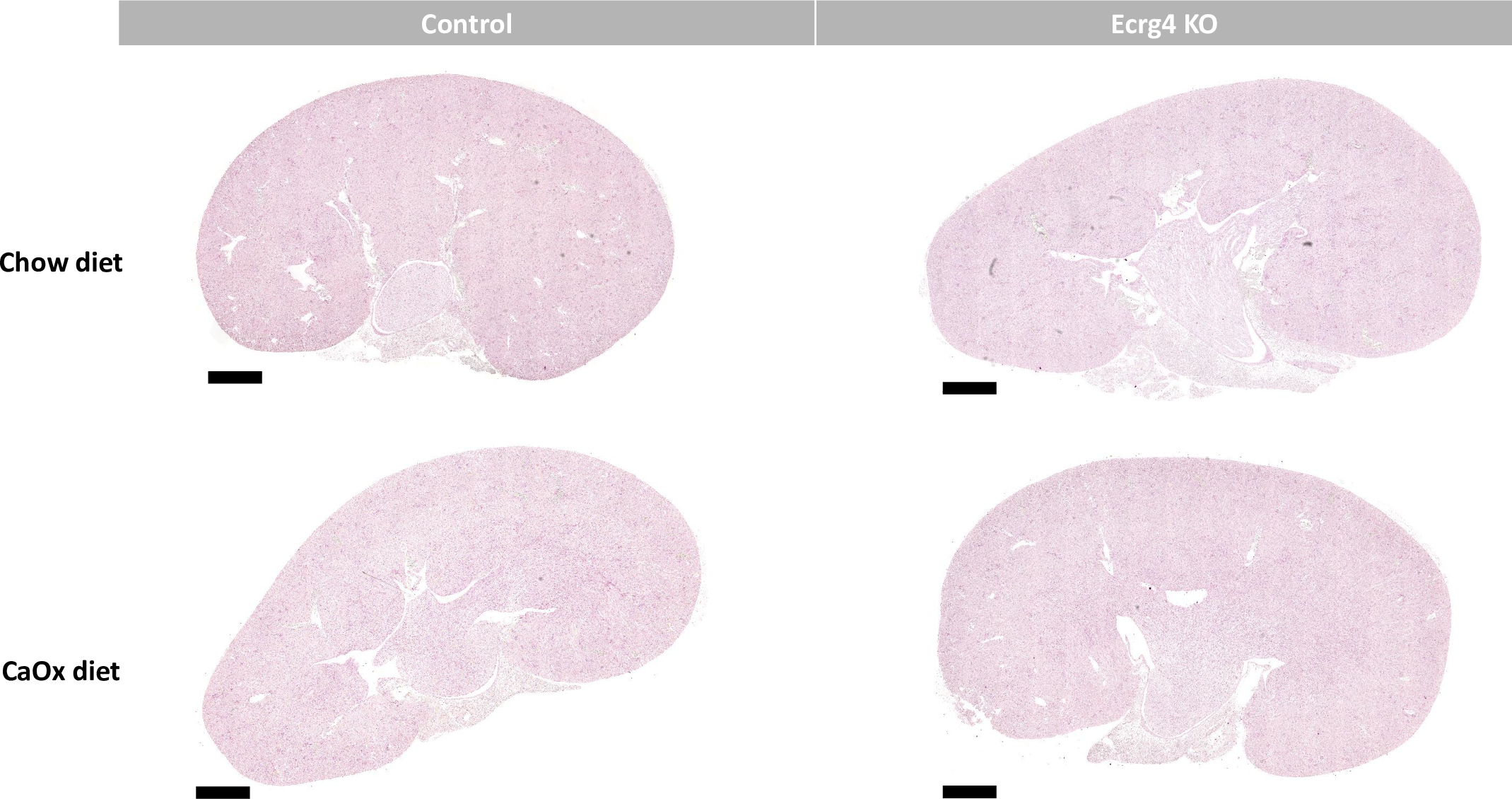

Supplement: S12 Fig — Representative pictures of the Pizzolato staining of the (A) control and (B) Ecrg4 KO female mice kidneys, under the CaOx. No calcium oxalate crystals can be observed in either of the kidney samples. Scale bars represent 1mm. (TIF) [file pone.0275972.s012.tif]

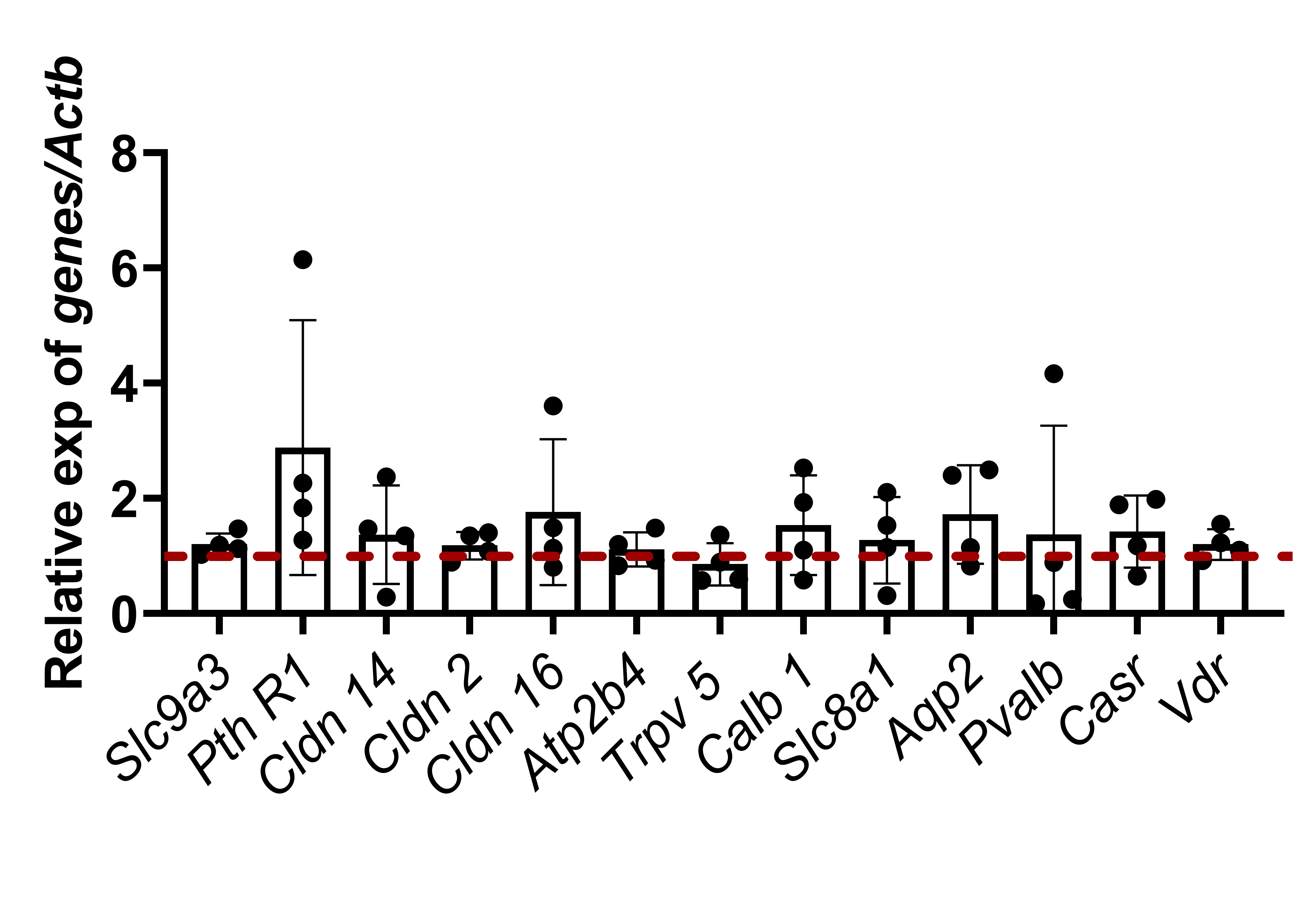

Supplement: S13 Fig — Relative expression of several renal genes involved calcium handling following the CaOx diet in Ecrg4 KO mice is illustrated relative to Actb and to controls (1, dashed line) (n = 4). Data are shown as mean ± SD. (TIF) [file pone.0275972.s013.tif]

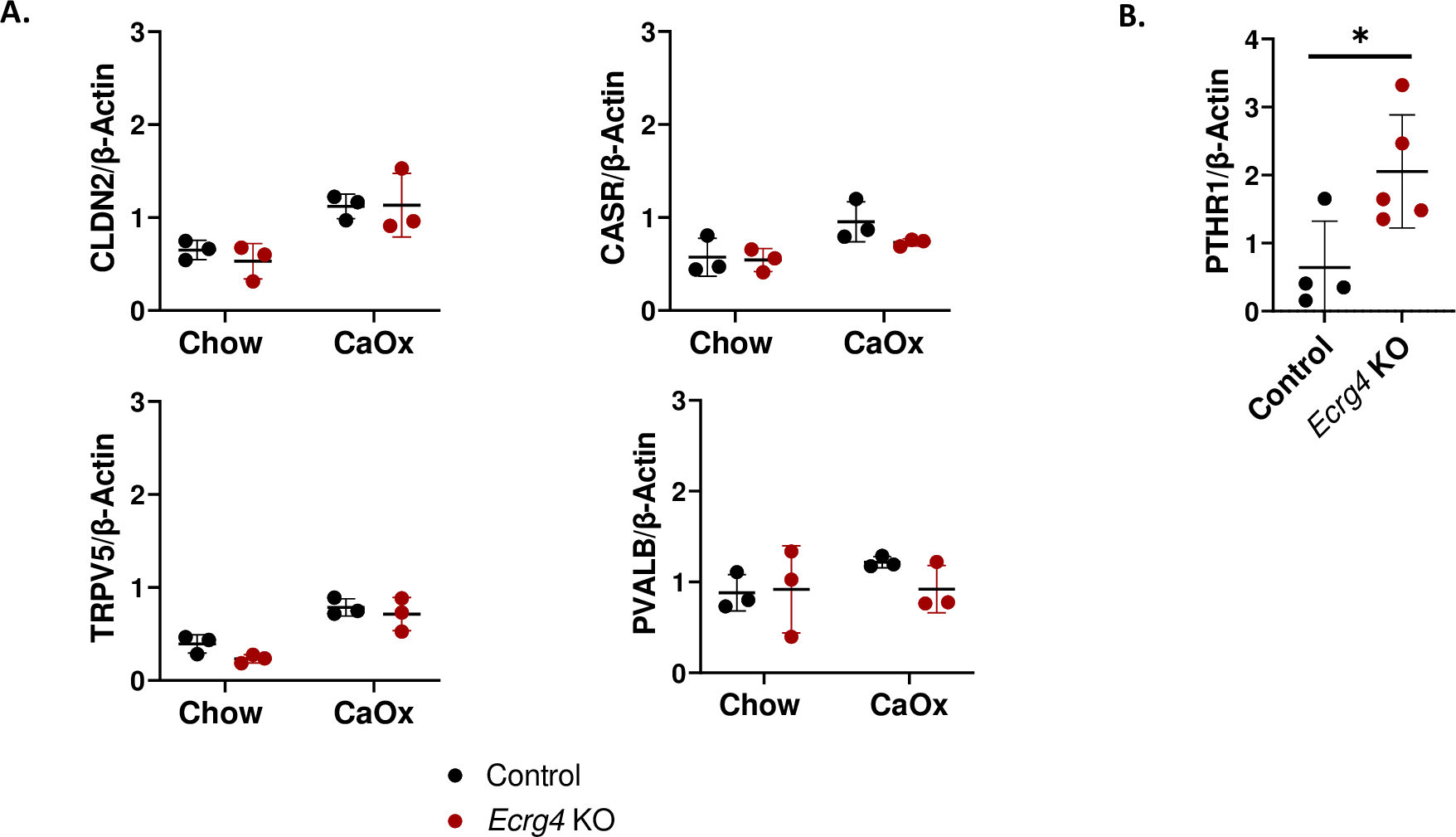

Supplement: S14 Fig — Western blot analysis of (A) CLDN2, TRPV5, PVALB, CASR proteins in male mice kidney tissue (n = 3). Mice were fed either the chow diet or challenged with the CaOx diet. Western blot analysis of (B) PTHR1 protein in control vs Ecrg4 KO male mice kidney tissue (n = 4–5). Mice were challenged with the CaOx diet. Quantities represented by the gel bands are expressed as intensity relative to β-Actin. Quantities represented by the gel bands are expressed as intensity relative to β-actin. All relative intensity results are presented as the means ± SD. Stars indicates * p<0.05. P values are calculated using two-way ANOVA test, with Sidak’s correction for multiple comparison (A) or Student t-test (B). (TIF) [file pone.0275972.s014.tif]

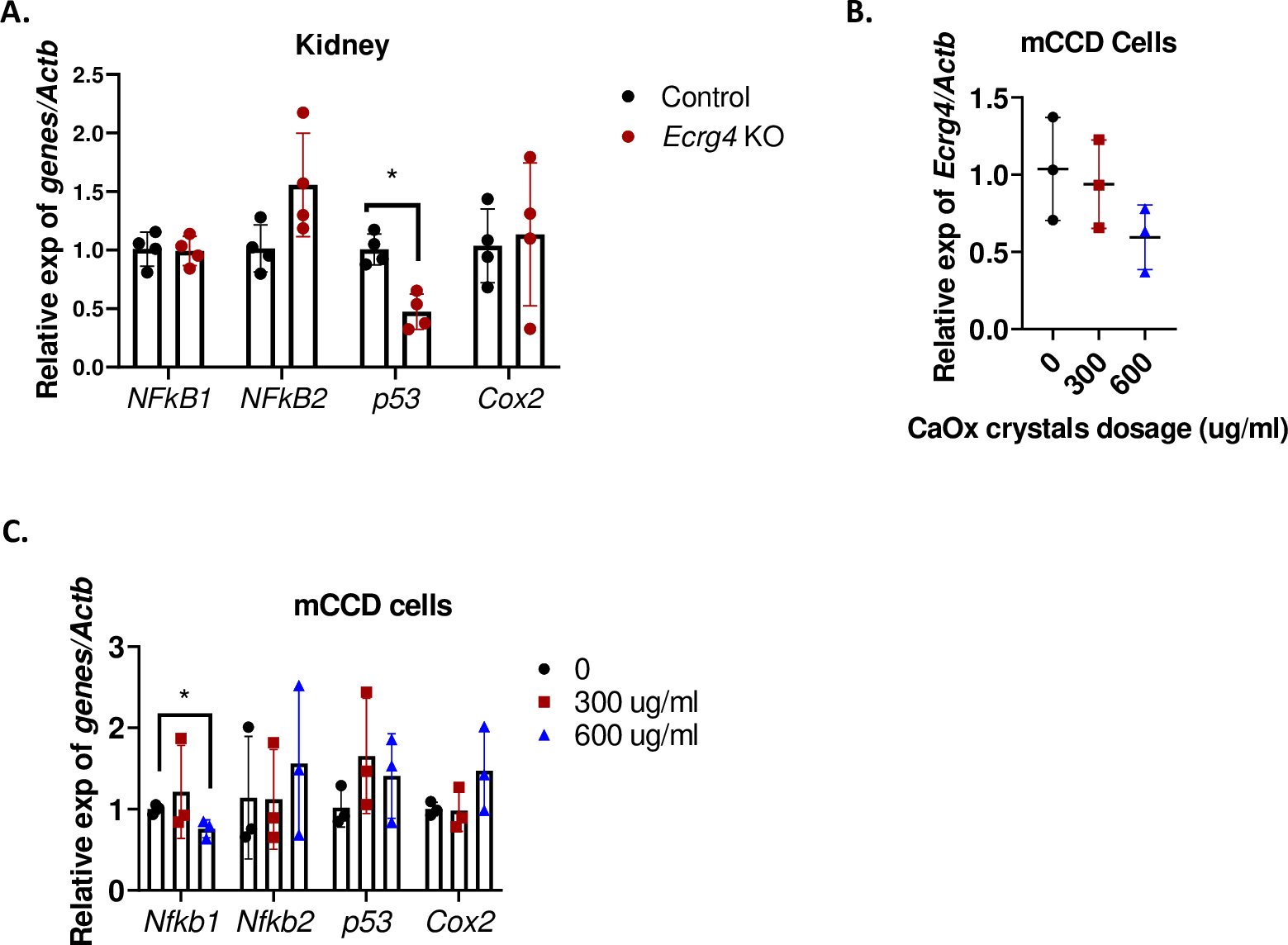

Supplement: S15 Fig — (A) The downstream genes that are activated upon ECRG4 signaling were quantified by qPCR in kidney extracts from control and Ecrg4 KO male mice. Stars indicates * p<0.05. P values are calculated using Student t-test (n = 4). (B) mCCD cells were incubated with CaOx monohydrate crystals for 16h. The Ecrg4 mRNA expression is shown as response to different crystals concentrations. (C) The downstream genes that are activated upon ECRG4 signaling were quantified in the mCCD cells upon calcium-oxalate monohydrate crystals incubation. Stars indicates * p<0.05. P values are calculated using one-way ANOVA comparing the mean of each CaOx crystal dosage (300 and 600 ug/ml CaOx) with the mean of the control column at 0 ug/ml CaOx crystal dosage (n = 3). Data are shown as mean ± SD. (TIF) [file pone.0275972.s015.tif]

Esophagus (100g protein)

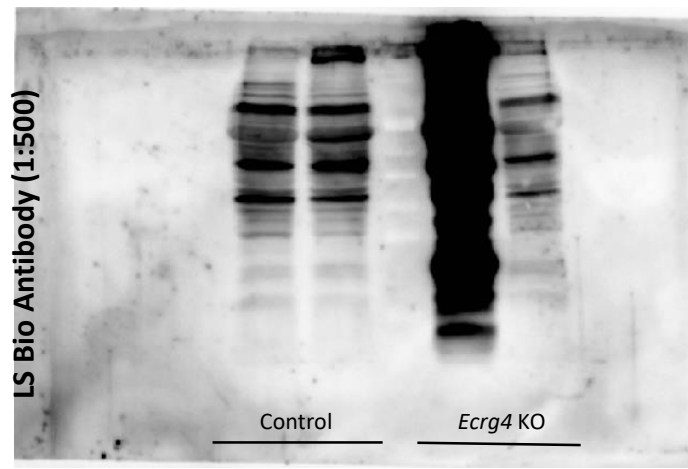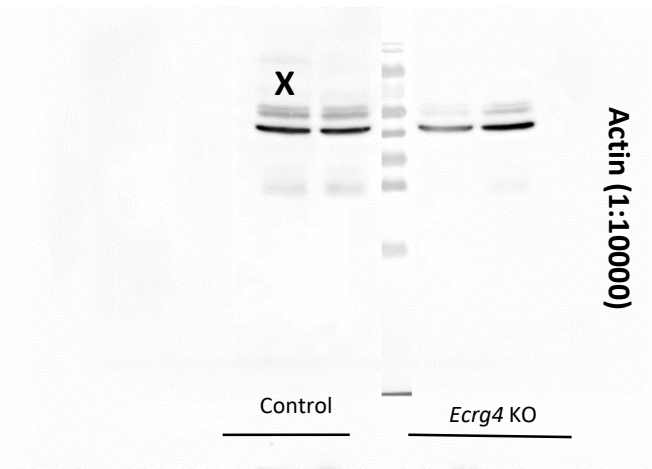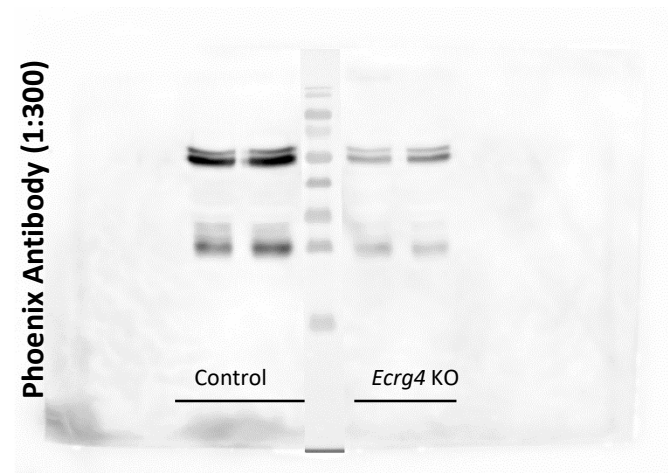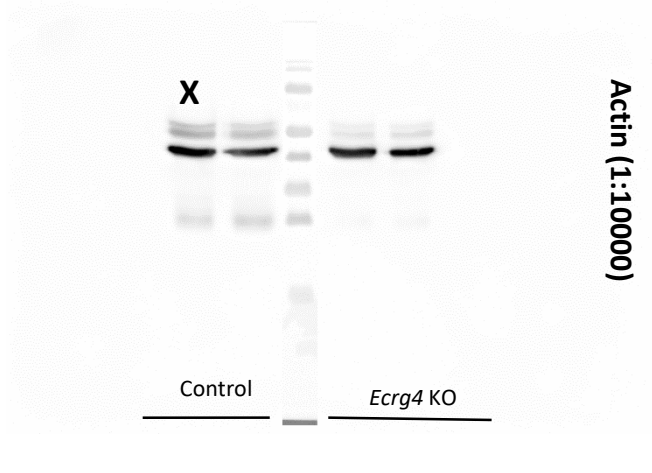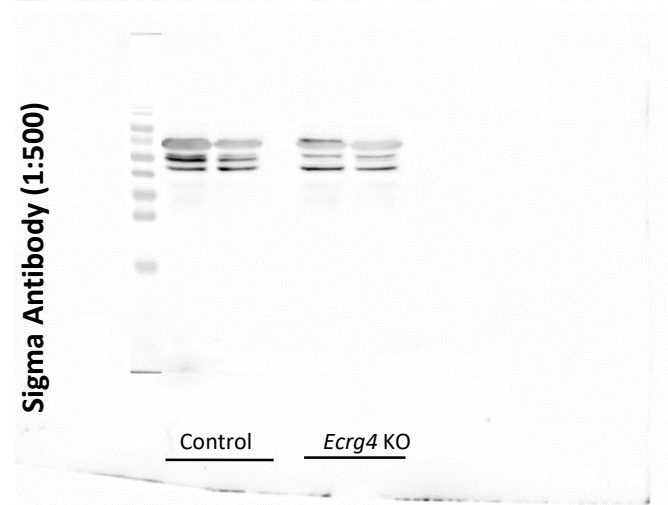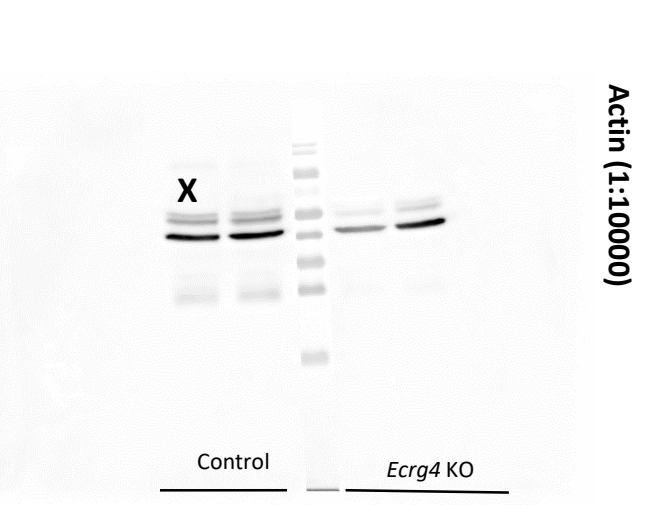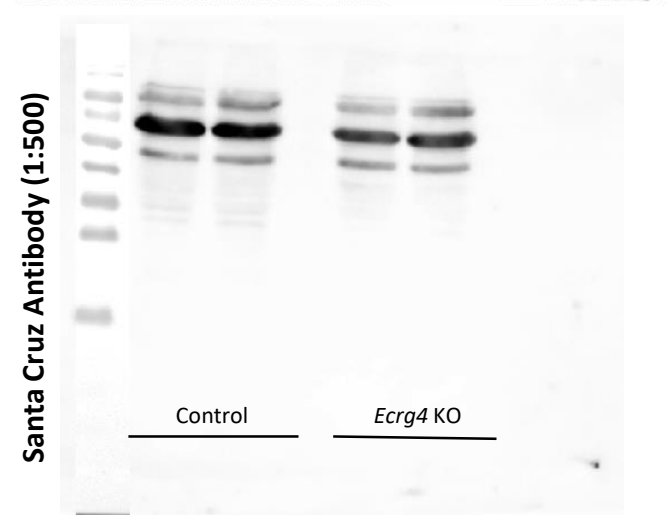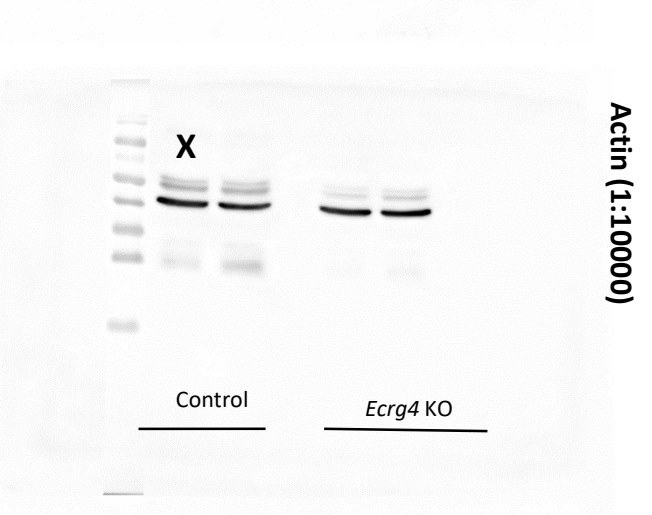

Supplement: S3 Raw images — (PDF) [file pone.0275972.s021.pdf]

Kidney (30g protein)

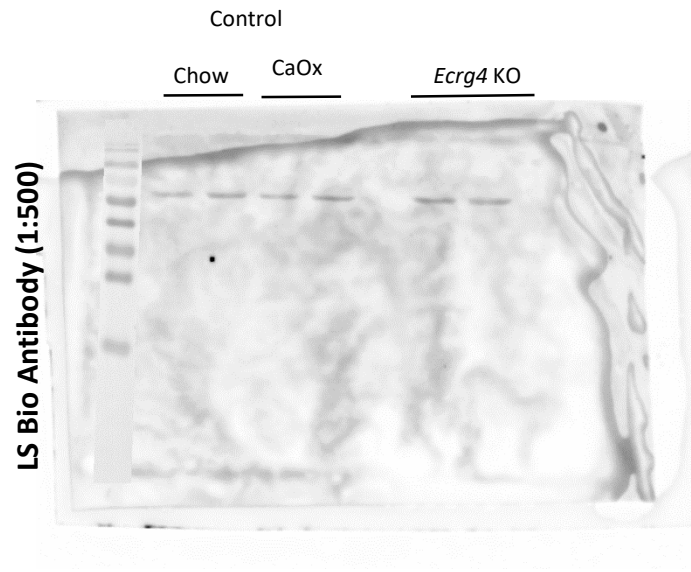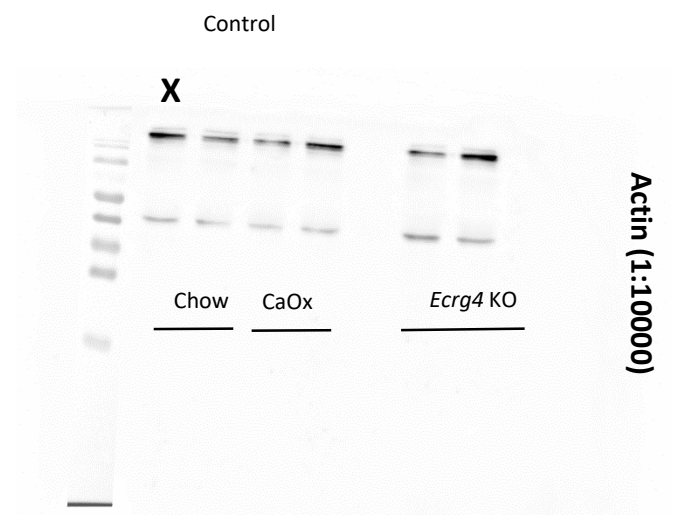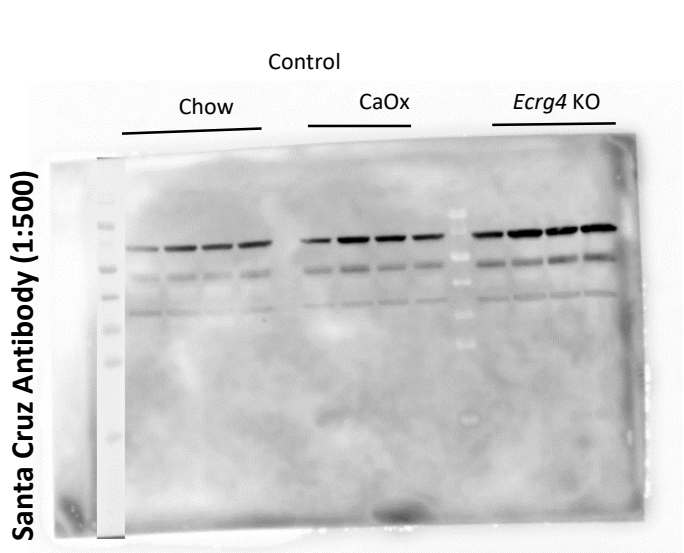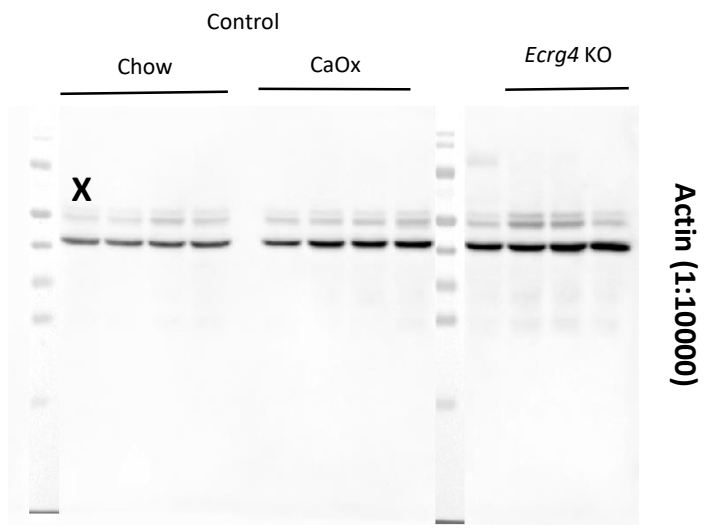

Supplement: S4 Raw images — (PDF) [file pone.0275972.s022.pdf]
