# Supplementary material for: Loss of Ecrg4 improves calcium oxalate nephropathy
Source: PLoS One. 2022 Oct 13;17(10):e0275972. doi: 10.1371/journal.pone.0275972 (PMC9560046; doi:10.1371/journal.pone.0275972)
Supplement: S1 Table — (DOCX) [file pone.0275972.s016.docx]

**Supplemental Table 1**. **Identified proteins by mass spectrometry in control heart and brain tissues**

| **Identified proteins** | **Alternate ID** | **Molecular Weight** | **Total spectrum count**  **WT Brain**  (below ±20kDA section) | **Total spectrum count**  **WT Heart** (below ±20kDA section) |
| --- | --- | --- | --- | --- |
| HBB1_MOUSE | Hbb-b1 | 16 kDa | 286 | 806 |
| HBB2_MOUSE | Hbb-b2 | 16 kDa | 119 | 310 |
| HBA_MOUSE | Hba | 15 kDa | 228 | 596 |
| MYG_MOUSE | Mb | 17 kDa | 1 | 302 |
| FABPH_MOUSE | Fabp3 | 15 kDa | 25 | 204 |
| COX6C_MOUSE | Cox6c | 8 kDa | 56 | 150 |
| CYC_MOUSE | Cycs | 12 kDa | 44 | 88 |
| CYC_BOVIN | CYCS | 12 kDa | 26 | 49 |
| ADT1_MOUSE | Slc25a4 | 33 kDa | 12 | 89 |
| ADT2_MOUSE | Slc25a5 | 33 kDa | 5 | 41 |
| ATP5I_MOUSE | Atp5me | 8 kDa | 37 | 91 |
| NDUAC_MOUSE | Ndufa12 | 17 kDa | 36 | 79 |
| FABP4_MOUSE | Fabp4 | 15 kDa | 4 | 78 |
| COX41_MOUSE | Cox4i1 | 20 kDa | 73 | 77 |
| NDKB_MOUSE | Nme2 | 17 kDa | 48 | 72 |
| NDUB7_MOUSE | Ndufb7 | 16 kDa | 32 | 64 |
| ATP5L_MOUSE | Atp5mg | 11 kDa | 27 | 64 |
| NDUA4_MOUSE | Ndufa4 | 9 kDa | 24 | 64 |
| COX5A_MOUSE | Cox5a | 16 kDa | 39 | 57 |
| COX5B_MOUSE | Cox5b | 14 kDa | 30 | 57 |
| NDUB8_MOUSE | Ndufb8 | 22 kDa | 26 | 54 |
| QCR7_MOUSE | Uqcrb | 14 kDa | 23 | 54 |
| NDUAD_MOUSE | Ndufa13 | 17 kDa | 31 | 53 |
| NDUA2_MOUSE | Ndufa2 | 11 kDa | 28 | 53 |
| NDKA_MOUSE | Nme1 | 17 kDa | 48 | 51 |
| ATPK_MOUSE | Atp5mf | 10 kDa | 16 | 51 |
| RS27A_MOUSE | Rps27a | 18 kDa | 76 | 50 |
| CX6B1_MOUSE | Cox6b1 | 10 kDa | 28 | 50 |
| QCR8_MOUSE | Uqcrq | 10 kDa | 22 | 50 |
| ARF1_MOUSE | Arf1 | 21 kDa | 103 | 32 |
| ARF3_MOUSE | Arf3 | 21 kDa | 103 | 31 |
| ARF5_MOUSE | Arf5 | 21 kDa | 72 | 27 |
| ARF4_MOUSE | Arf4 | 20 kDa | 58 | 23 |
| NDUB6_MOUSE | Ndufb6 | 16 kDa | 30 | 47 |
| PRDX5_MOUSE | Prdx5 | 22 kDa | 40 | 46 |
| UCRI_MOUSE | Uqcrfs1 | 29 kDa | 28 | 45 |
| ACO13_MOUSE | Acot13 | 15 kDa | 16 | 43 |
| QCR9_MOUSE | Uqcr10 | 7 kDa | 28 | 40 |
| MLRV_MOUSE | Myl2 | 19 kDa | 0 | 40 |
| NDUA7_MOUSE | Ndufa7 | 13 kDa | 18 | 39 |
| PPIA_MOUSE | Ppia | 18 kDa | 137 | 38 |
| USMG5_MOUSE | Atp5md | 6 kDa | 14 | 36 |
| PROF1_MOUSE | Pfn1 | 15 kDa | 53 | 35 |
| NDUA6_MOUSE | Ndufa6 | 15 kDa | 21 | 34 |
| NDUB4_MOUSE | Ndufb4 | 15 kDa | 23 | 33 |
| NDUA5_MOUSE | Ndufa5 | 13 kDa | 14 | 33 |
| NDUC2_MOUSE | Ndufc2 | 14 kDa | 19 | 32 |
| NDUB5_MOUSE | Ndufb5 | 22 kDa | 26 | 31 |
| ATPD_MOUSE | Atp5f1d | 18 kDa | 17 | 31 |
| NDUBB_MOUSE | Ndufb11 | 17 kDa | 17 | 30 |
| CX7A1_MOUSE | Cox7a1 | 9 kDa | 0 | 30 |
| CISD1_MOUSE | Cisd1 | 12 kDa | 23 | 29 |
| NDUA3_MOUSE | Ndufa3 | 9 kDa | 12 | 29 |
| C560_MOUSE | Sdhc | 18 kDa | 3 | 29 |
| PPLA_MOUSE | Pln | 6 kDa | 0 | 28 |
| IF5A1_MOUSE | Eif5a | 17 kDa | 27 | 27 |
| IF5A2_MOUSE | Eif5a2 | 17 kDa | 13 | 0 |
| NDUS4_MOUSE | Ndufs4 | 20 kDa | 16 | 27 |
| HBE_MOUSE | Hbb-y | 16 kDa | 0 | 27 |
| ATPB_MOUSE | Atp5f1b | 56 kDa | 24 | 25 |
| SODC_MOUSE | Sod1 | 16 kDa | 18 | 23 |
| MPC1_MOUSE | Mpc1 | 12 kDa | 17 | 23 |
| TTHY_MOUSE | Ttr | 16 kDa | 15 | 23 |
| HINT2_MOUSE | Hint2 | 17 kDa | 12 | 23 |
| NDUAB_MOUSE | Ndufa11 | 15 kDa | 7 | 23 |
| MIF_MOUSE | Mif | 13 kDa | 55 | 22 |
| CALM1_MOUSE (+2) | Calm1 | 17 kDa | 54 | 22 |
| MGST3_MOUSE | Mgst3 | 17 kDa | 24 | 22 |
| ATPA_MOUSE | Atp5f1a | 60 kDa | 19 | 22 |
| TOM22_MOUSE | Tomm22 | 16 kDa | 23 | 21 |
| F162A_MOUSE | Fam162a | 18 kDa | 12 | 21 |
| MTFP1_MOUSE | Mtfp1 | 18 kDa | 6 | 21 |
| ALBU_MOUSE | Alb | 69 kDa | 2 | 21 |
| DEST_MOUSE | Dstn | 19 kDa | 45 | 20 |
| UB2V1_MOUSE | Ube2v1 | 16 kDa | 28 | 18 |
| UB2V2_MOUSE | Ube2v2 | 16 kDa | 29 | 15 |
| MPC2_MOUSE | Mpc2 | 14 kDa | 13 | 20 |
| LEG1_MOUSE | Lgals1 | 15 kDa | 12 | 20 |
| MIC26_MOUSE | Apoo | 23 kDa | 11 | 20 |
| COQ7_MOUSE | Coq7 | 24 kDa | 8 | 20 |
| AT5G1_MOUSE (+2) | Atp5mc1 | 14 kDa | 19 | 20 |
| RHOA_MOUSE | Rhoa | 22 kDa | 40 | 18 |
| RHOC_MOUSE | Rhoc | 22 kDa | 0 | 13 |
| NDUS5_MOUSE | Ndufs5 | 13 kDa | 15 | 19 |
| MIC13_MOUSE | Mic13 | 13 kDa | 10 | 19 |
| NDUB3_MOUSE | Ndufb3 | 12 kDa | 9 | 19 |
| NDUS6_MOUSE | Ndufs6 | 13 kDa | 8 | 19 |
| QCR6_MOUSE | Uqcrh | 10 kDa | 7 | 19 |
| ATP5J_MOUSE | Atp5pf | 12 kDa | 6 | 19 |
| THIM_MOUSE | Acaa2 | 42 kDa | 0 | 19 |
| TNNC1_MOUSE | Tnnc1 | 18 kDa | 0 | 19 |
| COX7C_MOUSE | Cox7c | 7 kDa | 12 | 19 |
| ATP5E_MOUSE | Atp5f1e | 6 kDa | 18 | 19 |
| SYUA_MOUSE | Snca | 14 kDa | 50 | 18 |
| FABP5_MOUSE | Fabp5 | 15 kDa | 33 | 18 |
| CH10_MOUSE | Hspe1 | 11 kDa | 31 | 18 |
| NDUS7_MOUSE | Ndufs7 | 25 kDa | 12 | 17 |
| VDAC1_MOUSE | Vdac1 | 32 kDa | 12 | 17 |
| TM14C_MOUSE | Tmem14c | 12 kDa | 9 | 17 |
| MIC10_MOUSE | Minos1 | 9 kDa | 7 | 17 |
| DYL2_MOUSE | Dynll2 | 10 kDa | 38 | 13 |
| DYL1_MOUSE | Dynll1 | 10 kDa | 23 | 6 |
| FIS1_MOUSE | Fis1 | 17 kDa | 23 | 16 |
| RS19_MOUSE | Rps19 | 16 kDa | 10 | 16 |
| PPIF_MOUSE | Ppif | 22 kDa | 4 | 16 |
| CCD58_MOUSE | Ccdc58 | 17 kDa | 3 | 16 |
| COF1_MOUSE | Cfl1 | 19 kDa | 76 | 15 |
| RS17_MOUSE | Rps17 | 16 kDa | 15 | 15 |
| RS14_MOUSE | Rps14 | 16 kDa | 15 | 15 |
| HIG1A_MOUSE | Higd1a | 10 kDa | 10 | 15 |
| FUND2_MOUSE | Fundc2 | 17 kDa | 8 | 15 |
| COQ8A_MOUSE | Coq8a | 72 kDa | 0 | 15 |
| CX6A2_MOUSE | Cox6a2 | 11 kDa | 0 | 15 |
| DOPD_MOUSE | Ddt | 13 kDa | 19 | 14 |
| RS15_MOUSE | Rps15 | 17 kDa | 9 | 14 |
| ACPM_MOUSE | Ndufab1 | 17 kDa | 4 | 14 |
| ARL8B_MOUSE | Arl8b | 22 kDa | 29 | 14 |
| UBE2N_MOUSE | Ube2n | 17 kDa | 33 | 13 |
| HINT1_MOUSE | Hint1 | 14 kDa | 29 | 13 |
| RS13_MOUSE | Rps13 | 17 kDa | 12 | 13 |
| RS16_MOUSE | Rps16 | 16 kDa | 11 | 13 |
| TMM65_MOUSE | Tmem65 | 25 kDa | 10 | 13 |
| SLIRP_MOUSE | Slirp | 13 kDa | 8 | 13 |
| RT36_MOUSE | Mrps36 | 11 kDa | 3 | 13 |
| CAV3_MOUSE | Cav3 | 17 kDa | 0 | 13 |
| 68MP_MOUSE | Mp68 | 7 kDa | 9 | 13 |
| FKB1A_MOUSE | Fkbp1a | 12 kDa | 30 | 12 |
| ELOB_MOUSE | Elob | 13 kDa | 24 | 12 |
| PIN1_MOUSE | Pin1 | 18 kDa | 21 | 12 |
| ACBP_MOUSE | Dbi | 10 kDa | 20 | 12 |
| UB2L3_MOUSE | Ube2l3 | 18 kDa | 16 | 12 |
| ATIF1_MOUSE | ATP5IF1 | 12 kDa | 13 | 12 |
| THIO_MOUSE | Txn | 12 kDa | 10 | 9 |
| TIM13_MOUSE | Timm13 | 10 kDa | 9 | 12 |
| ELOC_MOUSE | Eloc | 12 kDa | 9 | 12 |
| PXMP2_MOUSE | Pxmp2 | 22 kDa | 2 | 12 |
| HSPB6_MOUSE | Hspb6 | 18 kDa | 0 | 12 |
| ARL8A_MOUSE | Arl8a | 21 kDa | 34 | 12 |
| VAMP3_MOUSE | Vamp3 | 11 kDa | 39 | 12 |
| VAMP2_MOUSE | Vamp2 | 13 kDa | 117 | 11 |
| PROF2_MOUSE | Pfn2 | 15 kDa | 25 | 11 |
| G3P_MOUSE | Gapdh | 36 kDa | 21 | 9 |
| G3P_BOVIN | GAPDH | 36 kDa | 7 | 3 |
| CYB5B_MOUSE | Cyb5b | 16 kDa | 21 | 11 |
| CX7A2_MOUSE | Cox7a2 | 9 kDa | 20 | 11 |
| ML12B_MOUSE | Myl12b | 20 kDa | 19 | 9 |
| MYL9_MOUSE | Myl9 | 20 kDa | 0 | 9 |
| ISC2A_MOUSE | Isoc2a | 22 kDa | 16 | 11 |
| NLTP_MOUSE | Scp2 | 59 kDa | 10 | 11 |
| RL30_MOUSE | Rpl30 | 13 kDa | 10 | 11 |
| TIM14_MOUSE | Dnajc19 | 12 kDa | 9 | 11 |
| MGDP1_MOUSE | Mdp1 | 19 kDa | 7 | 11 |
| MDHM_MOUSE | Mdh2 | 36 kDa | 5 | 11 |
| S10A1_MOUSE | S100a1 | 11 kDa | 3 | 11 |
| APOC3_MOUSE | Apoc3 | 11 kDa | 2 | 11 |
| ANF_MOUSE | Nppa | 17 kDa | 0 | 11 |
| RTN2_MOUSE | Rtn2 | 51 kDa | 0 | 11 |
| NDUB1_MOUSE | Ndufb1 | 7 kDa | 6 | 11 |
| NDUB2_MOUSE | Ndufb2 | 12 kDa | 3 | 11 |
| COF2_MOUSE | Cfl2 | 19 kDa | 32 | 10 |
| ARPC4_MOUSE | Arpc4 | 20 kDa | 25 | 10 |
| SNX3_MOUSE | Snx3 | 19 kDa | 17 | 10 |
| PPAC_MOUSE | Acp1 | 18 kDa | 12 | 10 |
| AP4A_MOUSE | Nudt2 | 17 kDa | 12 | 10 |
| CYB5_MOUSE | Cyb5a | 15 kDa | 10 | 10 |
| RM22_MOUSE | Mrpl22 | 24 kDa | 9 | 10 |
| F210A_MOUSE | Fam210a | 32 kDa | 9 | 10 |
| GLRX1_MOUSE | Glrx | 12 kDa | 8 | 10 |
| PHP14_MOUSE | Phpt1 | 14 kDa | 7 | 10 |
| RLA2_MOUSE | Rplp2 | 12 kDa | 7 | 10 |
| ATPO_MOUSE | Atp5po | 23 kDa | 6 | 10 |
| NDUA8_MOUSE | Ndufa8 | 20 kDa | 6 | 10 |
| NDUV3_MOUSE | Ndufv3 | 12 kDa | 4 | 10 |
| S10A6_MOUSE | S100a6 | 10 kDa | 3 | 10 |
| ETFB_MOUSE | Etfb | 28 kDa | 1 | 10 |
| MLRA_MOUSE | Myl7 | 19 kDa | 0 | 10 |
| CAV1_MOUSE | Cav1 | 21 kDa | 0 | 10 |
| PLM_MOUSE | Fxyd1 | 10 kDa | 8 | 10 |
| RAC1_MOUSE | Rac1 | 21 kDa | 33 | 9 |
| RAC3_MOUSE | Rac3 | 21 kDa | 23 | 0 |
| RAC2_MOUSE | Rac2 | 21 kDa | 18 | 0 |
| RS18_MOUSE | Rps18 | 18 kDa | 14 | 9 |
| RL23_MOUSE | Rpl23 | 15 kDa | 14 | 9 |
| NEDD8_MOUSE (+1) | Nedd8 | 9 kDa | 13 | 9 |
| TIM10_MOUSE | Timm10 | 10 kDa | 11 | 9 |
| T126A_MOUSE | Tmem126a | 22 kDa | 11 | 9 |
| EIF1_MOUSE | Eif1 | 13 kDa | 8 | 9 |
| EIF1B_MOUSE | Eif1b | 13 kDa | 6 | 0 |
| NTF2_MOUSE | Nutf2 | 14 kDa | 7 | 9 |
| PTMA_MOUSE | Ptma | 12 kDa | 7 | 9 |
| ACYP2_MOUSE | Acyp2 | 12 kDa | 7 | 9 |
| GLRX5_MOUSE | Glrx5 | 16 kDa | 7 | 9 |
| DAD1_MOUSE | Dad1 | 12 kDa | 5 | 9 |
| ARP19_MOUSE | Arpp19 | 12 kDa | 3 | 9 |
| COX8B_MOUSE | Cox8b | 7 kDa | 0 | 9 |
| RS21_MOUSE | Rps21 | 9 kDa | 10 | 9 |
| DHSD_MOUSE | Sdhd | 17 kDa | 0 | 9 |
| REEP5_MOUSE | Reep5 | 21 kDa | 5 | 9 |
| NDUA1_MOUSE | Ndufa1 | 8 kDa | 4 | 9 |
| DUS3_MOUSE | Dusp3 | 20 kDa | 19 | 8 |
| SNX12_MOUSE | Snx12 | 19 kDa | 17 | 8 |
| PFD2_MOUSE | Pfdn2 | 17 kDa | 15 | 8 |
| RL12_MOUSE | Rpl12 | 18 kDa | 11 | 8 |
| RL27_MOUSE | Rpl27 | 16 kDa | 11 | 8 |
| RHOG_MOUSE | Rhog | 21 kDa | 10 | 8 |
| RT28_MOUSE | Mrps28 | 21 kDa | 10 | 8 |
| RL32_MOUSE | Rpl32 | 16 kDa | 9 | 8 |
| RS12_MOUSE | Rps12 | 15 kDa | 9 | 8 |
| H2B1B_MOUSE (+7) | Hist1h2bb | 14 kDa | 7 | 8 |
| H2B2E_MOUSE (+2) | Hist2h2be | 14 kDa | 5 | 0 |
| BOLA1_MOUSE | Bola1 | 14 kDa | 6 | 8 |
| TAGL_MOUSE | Tagln | 23 kDa | 5 | 8 |
| ISCA2_MOUSE | Isca2 | 17 kDa | 5 | 8 |
| ACOX1_MOUSE | Acox1 | 75 kDa | 4 | 8 |
| MSRB2_MOUSE | Msrb2 | 19 kDa | 3 | 8 |
| TIM8B_MOUSE | Timm8b | 9 kDa | 3 | 8 |
| MPV17_MOUSE | Mpv17 | 20 kDa | 2 | 8 |
| IDHP_MOUSE | Idh2 | 51 kDa | 0 | 8 |
| HSPB7_MOUSE | Hspb7 | 19 kDa | 0 | 8 |
| ACADL_MOUSE | Acadl | 48 kDa | 0 | 8 |
| KCRM_MOUSE | Ckm | 43 kDa | 0 | 8 |
| ATP8_MOUSE | Mtatp8 | 8 kDa | 8 | 8 |
| CX6A1_MOUSE | Cox6a1 | 12 kDa | 29 | 8 |
| ROMO1_MOUSE | Romo1 | 8 kDa | 5 | 8 |
| PEA15_MOUSE | Pea15 | 15 kDa | 36 | 7 |
| VATF_MOUSE | Atp6v1f | 13 kDa | 29 | 7 |
| PRVA_MOUSE | Pvalb | 12 kDa | 26 | 7 |
| MYL6_MOUSE | Myl6 | 17 kDa | 14 | 7 |
| MYL6B_MOUSE | Myl6b | 23 kDa | 2 | 0 |
| VPS29_MOUSE | Vps29 | 20 kDa | 14 | 7 |
| RHEB_MOUSE | Rheb | 20 kDa | 14 | 7 |
| DLRB1_MOUSE | Dynlrb1 | 11 kDa | 13 | 7 |
| RS15A_MOUSE | Rps15a | 15 kDa | 12 | 7 |
| ISCU_MOUSE | Iscu | 18 kDa | 12 | 7 |
| RL31_MOUSE | Rpl31 | 14 kDa | 10 | 7 |
| PXL2B_MOUSE | Prxl2b | 22 kDa | 10 | 7 |
| AATM_MOUSE | Got2 | 47 kDa | 9 | 7 |
| PFD5_MOUSE | Pfdn5 | 17 kDa | 9 | 7 |
| MCA3_MOUSE | Eef1e1 | 20 kDa | 7 | 7 |
| NDUF3_MOUSE | Ndufaf3 | 21 kDa | 7 | 7 |
| SAP_MOUSE | Psap | 61 kDa | 7 | 7 |
| TMCO1_MOUSE | Tmco1 | 21 kDa | 7 | 7 |
| CISD2_MOUSE | Cisd2 | 15 kDa | 7 | 7 |
| LYRM4_MOUSE | Lyrm4 | 11 kDa | 6 | 7 |
| LSM2_MOUSE | Lsm2 | 11 kDa | 5 | 7 |
| RT06_MOUSE | Mrps6 | 14 kDa | 5 | 7 |
| TSPO_MOUSE | Tspo | 19 kDa | 1 | 7 |
| CRYAB_MOUSE | Cryab | 20 kDa | 0 | 7 |
| ECHB_MOUSE | Hadhb | 51 kDa | 0 | 7 |
| LDB3_MOUSE | Ldb3 | 76 kDa | 0 | 7 |
| COX7R_MOUSE | Cox7a2l | 12 kDa | 4 | 7 |
| PDLI5_MOUSE | Pdlim5 | 63 kDa | 0 | 7 |
| RS20_MOUSE | Rps20 | 13 kDa | 8 | 7 |
| RS28_MOUSE | Rps28 | 8 kDa | 7 | 7 |
| RHOB_MOUSE | Rhob | 22 kDa | 20 | 7 |
| HIG2A_MOUSE | Higd2a | 11 kDa | 3 | 7 |
| SKP1_MOUSE | Skp1 | 19 kDa | 24 | 6 |
| AP2S1_MOUSE | Ap2s1 | 17 kDa | 24 | 6 |
| STMN1_MOUSE | Stmn1 | 17 kDa | 19 | 6 |
| GMFB_MOUSE | Gmfb | 17 kDa | 18 | 6 |
| RIDA_MOUSE | Rida | 14 kDa | 14 | 6 |
| BAX_MOUSE | Bax | 21 kDa | 12 | 6 |
| TPPC3_MOUSE | Trappc3 | 20 kDa | 11 | 6 |
| LTOR1_MOUSE | Lamtor1 | 18 kDa | 10 | 6 |
| ANXA2_MOUSE | Anxa2 | 39 kDa | 9 | 6 |
| BRI3B_MOUSE | Bri3bp | 28 kDa | 8 | 6 |
| PFD1_MOUSE | Pfdn1 | 14 kDa | 6 | 6 |
| RL28_MOUSE | Rpl28 | 16 kDa | 6 | 6 |
| RM23_MOUSE | Mrpl23 | 17 kDa | 5 | 6 |
| RM13_MOUSE | Mrpl13 | 21 kDa | 5 | 6 |
| PDCD5_MOUSE | Pdcd5 | 14 kDa | 5 | 6 |
| CIA2B_MOUSE | Ciao2b | 18 kDa | 5 | 6 |
| MCEE_MOUSE | Mcee | 19 kDa | 4 | 6 |
| TXD12_MOUSE | Txndc12 | 19 kDa | 4 | 6 |
| BAF_MOUSE | Banf1 | 10 kDa | 4 | 6 |
| EF1A1_MOUSE | Eef1a1 | 50 kDa | 4 | 6 |
| ABRAL_MOUSE | Abracl | 9 kDa | 3 | 6 |
| ATP5H_MOUSE | Atp5pd | 19 kDa | 3 | 6 |
| QCR2_MOUSE | Uqcrc2 | 48 kDa | 1 | 6 |
| FRIH_MOUSE | Fth1 | 21 kDa | 1 | 6 |
| MPCP_MOUSE | Slc25a3 | 40 kDa | 0 | 6 |
| ISG15_MOUSE | Isg15 | 18 kDa | 0 | 6 |
| NU5M_MOUSE | Mtnd5 | 68 kDa | 0 | 6 |
| JAGN1_MOUSE | Jagn1 | 21 kDa | 6 | 6 |
| TOM20_MOUSE | Tomm20 | 16 kDa | 6 | 6 |
| M2OM_MOUSE | Slc25a11 | 34 kDa | 0 | 6 |
| GBG5_MOUSE | Gng5 | 7 kDa | 6 | 6 |
| UQCC2_MOUSE | Uqcc2 | 16 kDa | 5 | 6 |
| RM14_MOUSE | Mrpl14 | 16 kDa | 5 | 6 |
| VAPB_MOUSE | Vapb | 27 kDa | 10 | 6 |
| MDHC_MOUSE | Mdh1 | 37 kDa | 7 | 6 |
| CISY_MOUSE | Cs | 52 kDa | 1 | 6 |
| DYLT1_MOUSE | Dynlt1 | 12 kDa | 8 | 6 |
| VATL_MOUSE | Atp6v0c | 16 kDa | 36 | 6 |
| ANT3_MOUSE | Serpinc1 | 52 kDa | 0 | 6 |
| VAMP5_MOUSE | Vamp5 | 11 kDa | 0 | 6 |
| ACYP1_MOUSE | Acyp1 | 11 kDa | 16 | 5 |
| ARPC5_MOUSE | Arpc5 | 16 kDa | 16 | 5 |
| ARP5L_MOUSE | Arpc5l | 17 kDa | 11 | 5 |
| MCTS1_MOUSE | Mcts1 | 21 kDa | 11 | 5 |
| H2A2A_MOUSE (+1) | Hist2h2aa1 | 14 kDa | 7 | 5 |
| H2A2B_MOUSE | Hist2h2ab | 14 kDa | 3 | 0 |
| H2AV_MOUSE (+1) | H2afv | 14 kDa | 2 | 0 |
| GPX4_MOUSE | Gpx4 | 22 kDa | 9 | 5 |
| ARL1_MOUSE | Arl1 | 20 kDa | 9 | 5 |
| RLA1_MOUSE | Rplp1 | 11 kDa | 9 | 5 |
| PPIL1_MOUSE | Ppil1 | 18 kDa | 8 | 5 |
| SMD2_MOUSE | Snrpd2 | 14 kDa | 8 | 5 |
| CNPY2_MOUSE | Cnpy2 | 21 kDa | 8 | 5 |
| SYJ2B_MOUSE | Synj2bp | 16 kDa | 7 | 5 |
| NAA50_MOUSE | Naa50 | 19 kDa | 7 | 5 |
| F136A_MOUSE | Fam136a | 16 kDa | 6 | 5 |
| TXD17_MOUSE | Txndc17 | 14 kDa | 6 | 5 |
| RS10_MOUSE | Rps10 | 19 kDa | 6 | 5 |
| RS23_MOUSE | Rps23 | 16 kDa | 6 | 5 |
| TM109_MOUSE | Tmem109 | 26 kDa | 6 | 5 |
| RT10_MOUSE | Mrps10 | 19 kDa | 5 | 5 |
| MANF_MOUSE | Manf | 20 kDa | 4 | 5 |
| FRDA_MOUSE | Fxn | 23 kDa | 4 | 5 |
| SSBP_MOUSE | Ssbp1 | 17 kDa | 4 | 5 |
| RT11_MOUSE | Mrps11 | 20 kDa | 4 | 5 |
| RM48_MOUSE | Mrpl48 | 24 kDa | 4 | 5 |
| TIM16_MOUSE | Pam16 | 14 kDa | 3 | 5 |
| RL27A_MOUSE | Rpl27a | 17 kDa | 3 | 5 |
| TAGL2_MOUSE | Tagln2 | 22 kDa | 3 | 5 |
| COA3_MOUSE | Coa3 | 12 kDa | 2 | 5 |
| SDHB_MOUSE | Sdhb | 32 kDa | 1 | 5 |
| SSPN_MOUSE | Sspn | 24 kDa | 1 | 5 |
| ODPB_MOUSE | Pdhb | 39 kDa | 1 | 5 |
| MUP2_MOUSE | Mup2 | 21 kDa | 0 | 5 |
| ATPG_MOUSE | Atp5f1c | 33 kDa | 0 | 5 |
| COX20_MOUSE | Cox20 | 13 kDa | 0 | 5 |
| KAD1_MOUSE | Ak1 | 22 kDa | 0 | 5 |
| TIM8A_MOUSE | Timm8a1 | 11 kDa | 0 | 5 |
| ACADM_MOUSE | Acadm | 46 kDa | 0 | 5 |
| B3AT_MOUSE | Slc4a1 | 103 kDa | 0 | 5 |
| VATG1_MOUSE | Atp6v1g1 | 14 kDa | 8 | 5 |
| TIM9_MOUSE | Timm9 | 10 kDa | 5 | 5 |
| ETFA_MOUSE | Etfa | 35 kDa | 0 | 5 |
| AAMDC_MOUSE | Aamdc | 13 kDa | 4 | 5 |
| ALDOA_MOUSE (+1) | Aldoa | 39 kDa | 0 | 5 |
| RM55_MOUSE | Mrpl55 | 15 kDa | 4 | 5 |
| CSRP3_MOUSE | Csrp3 | 21 kDa | 0 | 5 |
| RS27_MOUSE | Rps27 | 9 kDa | 5 | 5 |
| RS27L_MOUSE | Rps27l | 9 kDa | 2 | 0 |
| APOC1_MOUSE | Apoc1 | 10 kDa | 0 | 5 |
| RL38_MOUSE | Rpl38 | 8 kDa | 14 | 5 |
| LDHB_MOUSE | Ldhb | 37 kDa | 4 | 5 |
| VAMP8_MOUSE | Vamp8 | 11 kDa | 3 | 5 |
| S10AD_MOUSE | S100a13 | 11 kDa | 3 | 5 |
| QCR10_MOUSE | Uqcr11 | 7 kDa | 7 | 5 |
| CRIP2_MOUSE | Crip2 | 23 kDa | 3 | 5 |
| MTPN_MOUSE | Mtpn | 13 kDa | 16 | 5 |
| GCSH_MOUSE | Gcsh | 19 kDa | 6 | 5 |
| HS90B_MOUSE | Hsp90ab1 | 83 kDa | 0 | 5 |
| ENPL_MOUSE | Hsp90b1 | 92 kDa | 2 | 0 |
| DMAC1_MOUSE | Dmac1 | 11 kDa | 4 | 5 |
| RS29_MOUSE | Rps29 | 7 kDa | 4 | 5 |
| TPC6B_MOUSE | Trappc6b | 18 kDa | 17 | 4 |
| RAB2A_MOUSE | Rab2a | 24 kDa | 15 | 4 |
| RAB2B_MOUSE | Rab2b | 24 kDa | 7 | 0 |
| DCTN3_MOUSE | Dctn3 | 21 kDa | 14 | 4 |
| BT3L4_MOUSE | Btf3l4 | 17 kDa | 13 | 4 |
| TBCA_MOUSE | Tbca | 13 kDa | 12 | 4 |
| PDCD6_MOUSE | Pdcd6 | 22 kDa | 12 | 4 |
| TEBP_MOUSE | Ptges3 | 19 kDa | 11 | 4 |
| RET1_MOUSE | Rbp1 | 16 kDa | 11 | 4 |
| ARF6_MOUSE | Arf6 | 20 kDa | 10 | 4 |
| COPZ1_MOUSE | Copz1 | 20 kDa | 9 | 4 |
| DBLOH_MOUSE | Diablo | 27 kDa | 7 | 4 |
| H4_MOUSE | Hist1h4a | 11 kDa | 7 | 4 |
| RPAB3_MOUSE | Polr2h | 17 kDa | 6 | 4 |
| RS25_MOUSE | Rps25 | 14 kDa | 6 | 4 |
| ATOX1_MOUSE | Atox1 | 7 kDa | 6 | 4 |
| ATP5S_MOUSE | Dmac2l | 23 kDa | 5 | 4 |
| SRP14_MOUSE | Srp14 | 13 kDa | 5 | 4 |
| RBM3_MOUSE | Rbm3 | 17 kDa | 4 | 4 |
| PTPM1_MOUSE | Ptpmt1 | 22 kDa | 4 | 4 |
| RM12_MOUSE | Mrpl12 | 22 kDa | 4 | 4 |
| RM50_MOUSE | Mrpl50 | 18 kDa | 4 | 4 |
| RL34_MOUSE | Rpl34 | 13 kDa | 4 | 4 |
| RM20_MOUSE | Mrpl20 | 18 kDa | 3 | 4 |
| TM186_MOUSE | Tmem186 | 25 kDa | 3 | 4 |
| TI17A_MOUSE | Timm17a | 18 kDa | 3 | 4 |
| AT5F1_MOUSE | Atp5pb | 29 kDa | 2 | 4 |
| RM49_MOUSE | Mrpl49 | 19 kDa | 2 | 4 |
| RT24_MOUSE | Mrps24 | 19 kDa | 1 | 4 |
| CDNF_MOUSE | Cdnf | 21 kDa | 0 | 4 |
| CO8G_MOUSE | C8g | 23 kDa | 0 | 4 |
| TMED2_MOUSE | Tmed2 | 23 kDa | 14 | 4 |
| UBL4A_MOUSE | Ubl4a | 18 kDa | 10 | 4 |
| TOM5_MOUSE | Tomm5 | 6 kDa | 9 | 4 |
| SH3L1_MOUSE | Sh3bgrl | 13 kDa | 8 | 4 |
| NDK3_MOUSE | Nme3 | 19 kDa | 5 | 4 |
| SSRD_MOUSE | Ssr4 | 19 kDa | 5 | 4 |
| LTOR5_MOUSE | Lamtor5 | 10 kDa | 3 | 4 |
| ETFR1_MOUSE | Etfrf1 | 10 kDa | 1 | 4 |
| RS11_MOUSE | Rps11 | 18 kDa | 5 | 4 |
| TMM11_MOUSE | Tmem11 | 21 kDa | 5 | 4 |
| CDC42_MOUSE | Cdc42 | 21 kDa | 12 | 4 |
| RM27_MOUSE | Mrpl27 | 16 kDa | 1 | 4 |
| PTPS_MOUSE | Pts | 16 kDa | 5 | 4 |
| EDF1_MOUSE | Edf1 | 16 kDa | 4 | 4 |
| DUS23_MOUSE | Dusp23 | 17 kDa | 3 | 4 |
| IF1AX_MOUSE | Eif1ax | 16 kDa | 5 | 4 |
| IF1A_MOUSE | Eif1a | 17 kDa | 5 | 0 |
| GMFG_MOUSE | Gmfg | 17 kDa | 0 | 4 |
| CD81_MOUSE | Cd81 | 26 kDa | 19 | 4 |
| PRAF3_MOUSE | Arl6ip5 | 22 kDa | 16 | 4 |
| TM35A_MOUSE | Tmem35a | 19 kDa | 5 | 4 |
| PDE6D_MOUSE | Pde6d | 17 kDa | 3 | 4 |
| SODM_MOUSE | Sod2 | 25 kDa | 2 | 4 |
| TCP4_MOUSE | Sub1 | 14 kDa | 13 | 4 |
| SAA4_MOUSE | Saa4 | 15 kDa | 0 | 4 |
| ERD21_MOUSE | Kdelr1 | 25 kDa | 4 | 4 |
| PRDX2_MOUSE | Prdx2 | 22 kDa | 5 | 4 |
| CYTC_MOUSE | Cst3 | 16 kDa | 16 | 4 |
| SH3L3_MOUSE | Sh3bgrl3 | 10 kDa | 12 | 4 |
| ADX_MOUSE | Fdx1 | 20 kDa | 1 | 4 |
| DHRS4_MOUSE | Dhrs4 | 30 kDa | 0 | 4 |
| SDHF1_MOUSE | Sdhaf1 | 13 kDa | 0 | 4 |
| RL35A_MOUSE | Rpl35a | 13 kDa | 7 | 4 |
| SMD1_MOUSE | Snrpd1 | 13 kDa | 5 | 4 |
| IFM3_MOUSE | Ifitm3 | 15 kDa | 1 | 4 |
| AP1S1_MOUSE | Ap1s1 | 19 kDa | 9 | 4 |
| COTL1_MOUSE | Cotl1 | 16 kDa | 24 | 4 |
| KCRS_MOUSE | Ckmt2 | 47 kDa | 0 | 4 |
| GBG12_MOUSE | Gng12 | 8 kDa | 10 | 4 |
| BRK1_MOUSE | Brk1 | 9 kDa | 6 | 4 |
| BOLA3_MOUSE | Bola3 | 12 kDa | 0 | 4 |
| COX17_MOUSE | Cox17 | 7 kDa | 0 | 4 |
| RM17_MOUSE | Mrpl17 | 20 kDa | 2 | 4 |
| APOC2_MOUSE | Apoc2 | 11 kDa | 0 | 4 |
| H31_MOUSE (+3) | Hist1h3a | 15 kDa | 2 | 4 |
| ASGL1_MOUSE | Asrgl1 | 34 kDa | 15 | 3 |
| ISCA1_MOUSE | Isca1 | 14 kDa | 7 | 3 |
| RS24_MOUSE | Rps24 | 15 kDa | 6 | 3 |
| UBE2B_MOUSE | Ube2b | 17 kDa | 5 | 2 |
| UBE2A_MOUSE | Ube2a | 17 kDa | 2 | 1 |
| FDX2_MOUSE | Fdx2 | 19 kDa | 6 | 3 |
| VDAC2_MOUSE | Vdac2 | 32 kDa | 5 | 3 |
| T126B_MOUSE | Tmem126b | 25 kDa | 2 | 3 |
| CDIPT_MOUSE | Cdipt | 24 kDa | 20 | 3 |
| CATD_MOUSE | Ctsd | 45 kDa | 11 | 3 |
| MGN_MOUSE | Magoh | 17 kDa | 8 | 3 |
| RM41_MOUSE | Mrpl41 | 15 kDa | 6 | 3 |
| DUS28_MOUSE | Dusp28 | 18 kDa | 4 | 3 |
| SNG1_MOUSE | Syngr1 | 26 kDa | 4 | 3 |
| CAV2_MOUSE | Cav2 | 18 kDa | 0 | 3 |
| T10B_MOUSE | Timm10b | 11 kDa | 6 | 3 |
| RL22_MOUSE | Rpl22 | 15 kDa | 5 | 3 |
| HOP_MOUSE | Hopx | 8 kDa | 1 | 3 |
| OPA1_MOUSE | Opa1 | 111 kDa | 3 | 3 |
| CEBOS_MOUSE | Cebpzos | 9 kDa | 1 | 3 |
| MUP20_MOUSE | Mup20 | 21 kDa | 0 | 3 |
| RM18_MOUSE | Mrpl18 | 21 kDa | 0 | 3 |
| CHAC2_MOUSE | Chac2 | 20 kDa | 2 | 3 |
| MYDGF_MOUSE | Mydgf | 18 kDa | 4 | 3 |
| TM223_MOUSE | Tmem223 | 22 kDa | 0 | 3 |
| ANXA1_MOUSE | Anxa1 | 39 kDa | 0 | 3 |
| TM230_MOUSE | Tmem230 | 13 kDa | 3 | 3 |
| PHS2_MOUSE | Pcbd2 | 15 kDa | 3 | 3 |
| TPIS_MOUSE | Tpi1 | 32 kDa | 2 | 3 |
| RT21_MOUSE | Mrps21 | 11 kDa | 1 | 3 |
| QCR1_MOUSE | Uqcrc1 | 53 kDa | 0 | 3 |
| TBA4A_MOUSE | Tuba4a | 50 kDa | 35 | 3 |
| TBA1A_MOUSE | Tuba1a | 50 kDa | 39 | 0 |
| AATC_MOUSE | Got1 | 46 kDa | 3 | 3 |
| CAVN1_MOUSE | Cavin1 | 44 kDa | 0 | 3 |
| TPPC1_MOUSE | Trappc1 | 17 kDa | 7 | 3 |
| CAR19_MOUSE | Card19 | 21 kDa | 0 | 3 |
| NUDC2_MOUSE | Nudcd2 | 18 kDa | 5 | 3 |
| S10A4_MOUSE | S100a4 | 12 kDa | 1 | 3 |
| ASPG_MOUSE | Aga | 37 kDa | 1 | 3 |
| PSN1_MOUSE | Psen1 | 53 kDa | 5 | 3 |
| RGCC_MOUSE | Rgcc | 15 kDa | 0 | 3 |
| CSN8_MOUSE | Cops8 | 23 kDa | 6 | 3 |
| RL37A_MOUSE | Rpl37a | 10 kDa | 4 | 3 |
| UBC9_MOUSE (+1) | Ube2i | 18 kDa | 9 | 3 |
| THIL_MOUSE | Acat1 | 45 kDa | 7 | 3 |
| IDH3A_MOUSE | Idh3a | 40 kDa | 0 | 3 |
| OPA3_MOUSE | Opa3 | 20 kDa | 3 | 3 |
| ATP6_MOUSE | Mtatp6 | 25 kDa | 1 | 3 |
| RBX1_MOUSE | Rbx1 | 12 kDa | 8 | 3 |
| SVIP_MOUSE | Svip | 8 kDa | 7 | 3 |
| SMD3_MOUSE | Snrpd3 | 14 kDa | 6 | 3 |
| LTOR3_MOUSE | Lamtor3 | 14 kDa | 5 | 3 |
| C10_MOUSE | Grcc10 | 13 kDa | 5 | 3 |
| PIN4_MOUSE | Pin4 | 14 kDa | 4 | 3 |
| RS26_MOUSE | Rps26 | 13 kDa | 3 | 3 |
| SC61B_MOUSE | Sec61b | 10 kDa | 2 | 3 |
| APOC4_MOUSE | Apoc4 | 14 kDa | 0 | 3 |
| SMIM4_MOUSE | Smim4 | 10 kDa | 0 | 3 |
| ARL6_MOUSE | Arl6 | 21 kDa | 11 | 3 |
| SUMO2_MOUSE | Sumo2 | 11 kDa | 4 | 3 |
| SUMO3_MOUSE | Sumo3 | 12 kDa | 3 | 0 |
| GPX7_MOUSE | Gpx7 | 21 kDa | 1 | 3 |
| FMC1_MOUSE | Fmc1 | 13 kDa | 2 | 3 |
| LDHA_MOUSE | Ldha | 36 kDa | 0 | 3 |
| SIM12_MOUSE | Smim12 | 11 kDa | 3 | 3 |
| S10AB_MOUSE | S100a11 | 11 kDa | 1 | 3 |
| CISD3_MOUSE | Cisd3 | 16 kDa | 3 | 3 |
| FHIT_MOUSE | Fhit | 17 kDa | 7 | 3 |
| IR3IP_MOUSE | Ier3ip1 | 9 kDa | 6 | 3 |
| RM54_MOUSE | Mrpl54 | 15 kDa | 0 | 3 |
| PRDX3_MOUSE | Prdx3 | 28 kDa | 4 | 3 |
| BOLA2_MOUSE | Bola2 | 10 kDa | 7 | 3 |
| SCOT1_MOUSE | Oxct1 | 56 kDa | 0 | 3 |
| SYUG_MOUSE | Sncg | 13 kDa | 15 | 3 |
| LYRM9_MOUSE | Lyrm9 | 9 kDa | 1 | 3 |
| UQCC3_MOUSE | Uqcc3 | 10 kDa | 0 | 3 |
| RT33_MOUSE | Mrps33 | 12 kDa | 3 | 3 |
| LAGE3_MOUSE | Lage3 | 16 kDa | 2 | 3 |
| UB2D2_MOUSE | Ube2d2 | 17 kDa | 8 | 2 |
| UB2D3_MOUSE | Ube2d3 | 17 kDa | 8 | 2 |
| ENOB_MOUSE | Eno3 | 47 kDa | 0 | 3 |
| UB2L6_MOUSE | Ube2l6 | 18 kDa | 0 | 3 |
| TOM7_MOUSE | Tomm7 | 6 kDa | 4 | 3 |
| SYUB_MOUSE | Sncb | 14 kDa | 54 | 3 |
| MBP_MOUSE | Mbp | 27 kDa | 142 | 3 |
| RTN3_MOUSE | Rtn3 | 104 kDa | 4 | 3 |
| HBBZ_MOUSE | Hbb-bh1 | 16 kDa | 1 | 3 |
| NEUG_MOUSE | Nrgn | 7 kDa | 30 | 3 |
| SC61G_MOUSE | Sec61g | 8 kDa | 3 | 3 |
| PLP2_MOUSE | Plp2 | 17 kDa | 2 | 3 |
| SMIM1_MOUSE | Smim1 | 9 kDa | 2 | 3 |
| ABCE1_MOUSE | Abce1 | 67 kDa | 0 | 3 |
| PRXD1_MOUSE | Prorsd1 | 19 kDa | 0 | 2 |
| NAA20_MOUSE | Naa20 | 20 kDa | 6 | 2 |
| B2MG_MOUSE | B2m | 14 kDa | 1 | 2 |
| PTH2_MOUSE | Ptrh2 | 20 kDa | 3 | 2 |
| MYOZ2_MOUSE | Myoz2 | 30 kDa | 0 | 2 |
| NENF_MOUSE | Nenf | 19 kDa | 6 | 2 |
| ENOA_MOUSE | Eno1 | 47 kDa | 9 | 2 |
| UB2G1_MOUSE | Ube2g1 | 20 kDa | 5 | 2 |
| AT1B1_MOUSE | Atp1b1 | 35 kDa | 6 | 2 |
| CHCH7_MOUSE | Chchd7 | 10 kDa | 1 | 2 |
| CYTB_MOUSE | Cstb | 11 kDa | 5 | 2 |
| KCC2A_MOUSE | Camk2a | 54 kDa | 6 | 2 |
| ARL2_MOUSE | Arl2 | 21 kDa | 5 | 2 |
| LTOR2_MOUSE | Lamtor2 | 13 kDa | 5 | 2 |
| TIM22_MOUSE | Timm22 | 20 kDa | 5 | 2 |
| EMC4_MOUSE | Emc4 | 20 kDa | 4 | 2 |
| MGST1_MOUSE | Mgst1 | 18 kDa | 4 | 2 |
| CIRBP_MOUSE | Cirbp | 19 kDa | 3 | 2 |
| THIOM_MOUSE | Txn2 | 18 kDa | 3 | 2 |
| SNAPN_MOUSE | Snapin | 15 kDa | 3 | 2 |
| COXM1_MOUSE | Cmc1 | 13 kDa | 2 | 2 |
| RT14_MOUSE | Mrps14 | 15 kDa | 1 | 2 |
| TX1B3_MOUSE | Tax1bp3 | 14 kDa | 1 | 2 |
| S10A9_MOUSE | S100a9 | 13 kDa | 1 | 2 |
| JOS2_MOUSE | Josd2 | 21 kDa | 1 | 2 |
| RL36A_MOUSE | Rpl36a | 12 kDa | 1 | 2 |
| APOA2_MOUSE | Apoa2 | 11 kDa | 0 | 2 |
| CNN1_MOUSE | Cnn1 | 33 kDa | 0 | 2 |
| FBX50_MOUSE | Nccrp1 | 30 kDa | 0 | 2 |
| HSDL2_MOUSE | Hsdl2 | 54 kDa | 0 | 2 |
| TPC2L_MOUSE | Trappc2l | 16 kDa | 6 | 2 |
| CO040_MOUSE |  | 13 kDa | 1 | 2 |
| FGF1_MOUSE | Fgf1 | 17 kDa | 5 | 2 |
| TOM6_MOUSE | Tomm6 | 8 kDa | 5 | 2 |
| COMD9_MOUSE | Commd9 | 22 kDa | 7 | 2 |
| VTNC_MOUSE | Vtn | 55 kDa | 0 | 2 |
| RM43_MOUSE | Mrpl43 | 20 kDa | 5 | 2 |
| KAP0_MOUSE | Prkar1a | 43 kDa | 0 | 2 |
| RT63_MOUSE | Mrpl57 | 12 kDa | 0 | 2 |
| SC11A_MOUSE | Sec11a | 21 kDa | 2 | 2 |
| CQ10B_MOUSE | Coq10b | 27 kDa | 0 | 2 |
| OARD1_MOUSE | Oard1 | 17 kDa | 2 | 2 |
| RT12_MOUSE | Mrps12 | 15 kDa | 2 | 2 |
| COA4_MOUSE | COA4 | 10 kDa | 0 | 2 |
| MASU1_MOUSE | Malsu1 | 26 kDa | 1 | 2 |
| S10AG_MOUSE | S100a16 | 14 kDa | 4 | 2 |
| LSM8_MOUSE | Lsm8 | 10 kDa | 4 | 2 |
| ACON_MOUSE | Aco2 | 85 kDa | 2 | 2 |
| PRAF1_MOUSE | Rabac1 | 21 kDa | 2 | 2 |
| HINT3_MOUSE | Hint3 | 19 kDa | 3 | 2 |
| SRCA_MOUSE | Srl | 99 kDa | 0 | 2 |
| EBP_MOUSE | Ebp | 26 kDa | 3 | 2 |
| ECI1_MOUSE | Eci1 | 32 kDa | 0 | 2 |
| TMM70_MOUSE | Tmem70 | 28 kDa | 0 | 2 |
| GBG10_MOUSE | Gng10 | 7 kDa | 2 | 2 |
| NTPCR_MOUSE | Ntpcr | 21 kDa | 2 | 2 |
| LSM6_MOUSE | Lsm6 | 9 kDa | 3 | 2 |
| PARK7_MOUSE | Park7 | 20 kDa | 3 | 2 |
| RM21_MOUSE | Mrpl21 | 23 kDa | 4 | 2 |
| AP3S2_MOUSE | Ap3s2 | 22 kDa | 4 | 2 |
| CHCH2_MOUSE | Chchd2 | 16 kDa | 1 | 2 |
| UB2D1_MOUSE | Ube2d1 | 17 kDa | 5 | 2 |
| NPM_MOUSE | Npm1 | 33 kDa | 3 | 2 |
| LYRM7_MOUSE | Lyrm7 | 12 kDa | 2 | 2 |
| PT100_MOUSE | Pet100 | 9 kDa | 0 | 2 |
| GBG2_MOUSE | Gng2 | 8 kDa | 17 | 2 |
| HS71A_MOUSE (+1) | Hspa1a | 70 kDa | 5 | 2 |
| ACBD5_MOUSE | Acbd5 | 57 kDa | 2 | 2 |
| VDAC3_MOUSE | Vdac3 | 31 kDa | 0 | 2 |
| TI17B_MOUSE | Timm17b | 18 kDa | 4 | 2 |
| COMD7_MOUSE | Commd7 | 23 kDa | 6 | 2 |
| KCNC1_MOUSE | Kcnc1 | 58 kDa | 0 | 2 |
| TM256_MOUSE | Tmem256 | 12 kDa | 6 | 2 |
| DDA1_MOUSE | Dda1 | 12 kDa | 3 | 2 |
| MYL3_MOUSE | Myl3 | 22 kDa | 0 | 2 |
| AP1S2_MOUSE | Ap1s2 | 19 kDa | 3 | 2 |
| MCFD2_MOUSE | Mcfd2 | 16 kDa | 2 | 2 |
| ERH_MOUSE | Erh | 12 kDa | 4 | 2 |
| VAPA_MOUSE | Vapa | 28 kDa | 10 | 2 |
| VAMP1_MOUSE | Vamp1 | 13 kDa | 50 | 2 |
| ENSA_MOUSE | Ensa | 13 kDa | 3 | 2 |
| DPOE3_MOUSE | Pole3 | 17 kDa | 0 | 2 |
| RM30_MOUSE | Mrpl30 | 18 kDa | 1 | 2 |
| TPRKB_MOUSE | Tprkb | 20 kDa | 4 | 2 |
| RUXE_MOUSE | Snrpe | 11 kDa | 6 | 2 |
| MYL4_MOUSE | Myl4 | 21 kDa | 0 | 2 |
| IFM2_MOUSE | Ifitm2 | 16 kDa | 0 | 2 |
| RUXG_MOUSE | Snrpg | 8 kDa | 5 | 2 |
| MIA40_MOUSE | Chchd4 | 16 kDa | 3 | 2 |
| RUXF_MOUSE | Snrpf | 10 kDa | 3 | 2 |
| RM34_MOUSE | Mrpl34 | 11 kDa | 2 | 2 |
| CD003_MOUSE |  | 7 kDa | 2 | 2 |
| IVD_MOUSE | Ivd | 46 kDa | 0 | 2 |
| SMIM5_MOUSE | Smim5 | 9 kDa | 0 | 2 |
| SEP15_MOUSE | Selenof | 18 kDa | 2 | 2 |
| NU3M_MOUSE | Mtnd3 | 13 kDa | 2 | 2 |
| RM32_MOUSE | Mrpl32 | 22 kDa | 3 | 2 |
| ODPA_MOUSE | Pdha1 | 43 kDa | 1 | 2 |
| CSMT1_MOUSE | Ccsmst1 | 15 kDa | 1 | 2 |
| GLPC_MOUSE | Gypc | 10 kDa | 0 | 2 |
| COX16_MOUSE | Cox16 | 12 kDa | 0 | 2 |
| SSRG_MOUSE | Ssr3 | 21 kDa | 1 | 2 |
| CO061_MOUSE |  | 18 kDa | 1 | 2 |
| COX3_MOUSE | mt-Co3 | 30 kDa | 0 | 2 |
| CD151_MOUSE | Cd151 | 28 kDa | 0 | 2 |
| EEA1_MOUSE | Eea1 | 161 kDa | 1 | 2 |
| TM242_MOUSE | Tmem242 | 15 kDa | 1 | 2 |
| SORCN_MOUSE | Sri | 22 kDa | 8 | 1 |
| SMPX_MOUSE | Smpx | 9 kDa | 0 | 1 |
| RFESD_MOUSE | Rfesd | 18 kDa | 1 | 1 |
| RL35_MOUSE | Rpl35 | 15 kDa | 2 | 1 |
| TITIN_MOUSE | Ttn | 3906 kDa | 0 | 1 |
| PA216_MOUSE | Pla2g16 | 18 kDa | 7 | 1 |
| CHSP1_MOUSE | Carhsp1 | 16 kDa | 4 | 1 |
| UFC1_MOUSE | Ufc1 | 19 kDa | 6 | 1 |
| 1433Z_MOUSE | Ywhaz | 28 kDa | 4 | 1 |
| 1433E_MOUSE | Ywhae | 29 kDa | 3 | 0 |
| 1433F_MOUSE | Ywhah | 28 kDa | 3 | 0 |
| 1433B_MOUSE | Ywhab | 28 kDa | 1 | 0 |
| 1433T_MOUSE | Ywhaq | 28 kDa | 1 | 0 |
| CMC1_MOUSE | Slc25a12 | 75 kDa | 0 | 1 |
| GGCT_MOUSE | Ggct | 21 kDa | 7 | 1 |
| SDHF4_MOUSE | Sdhaf4 | 12 kDa | 1 | 1 |
| COXM2_MOUSE | Cmc2 | 9 kDa | 1 | 1 |
| LY6C1_MOUSE | Ly6c1 | 14 kDa | 0 | 1 |
| NPC2_MOUSE | Npc2 | 16 kDa | 2 | 1 |
| UFM1_MOUSE | Ufm1 | 9 kDa | 4 | 1 |
| MIC27_MOUSE | Apool | 29 kDa | 0 | 1 |
| CANB1_MOUSE | Ppp3r1 | 19 kDa | 28 | 1 |
| IMP1L_MOUSE | Immp1l | 19 kDa | 0 | 1 |
| T2AG_MOUSE | Gtf2a2 | 12 kDa | 2 | 1 |
| RM33_MOUSE | Mrpl33 | 7 kDa | 0 | 1 |
| FKB1B_MOUSE | Fkbp1b | 12 kDa | 6 | 1 |
| POF1B_MOUSE | Pof1b | 68 kDa | 2 | 1 |
| PTRD1_MOUSE | Ptrhd1 | 16 kDa | 3 | 1 |
| HSPB3_MOUSE | Hspb3 | 17 kDa | 0 | 1 |
| CATC_MOUSE | Ctsc | 52 kDa | 0 | 1 |
| SNP23_MOUSE | Snap23 | 23 kDa | 0 | 1 |
| 4EBP1_MOUSE | Eif4ebp1 | 12 kDa | 0 | 1 |
| ANK1_MOUSE | Ank1 | 204 kDa | 0 | 1 |
| MARE1_MOUSE | Mapre1 | 30 kDa | 9 | 1 |
| COX2_MOUSE | Mtco2 | 26 kDa | 0 | 1 |
| SUCB1_MOUSE | Sucla2 | 50 kDa | 3 | 1 |
| RL36_MOUSE | Rpl36 | 12 kDa | 4 | 1 |
| PLRKT_MOUSE | Plgrkt | 17 kDa | 6 | 1 |
| FUND1_MOUSE | Fundc1 | 17 kDa | 6 | 1 |
| RARR2_MOUSE | Rarres2 | 18 kDa | 0 | 1 |
| CD9_MOUSE | Cd9 | 25 kDa | 5 | 1 |
| ENV1_MOUSE |  | 70 kDa | 2 | 1 |
| PFD6_MOUSE | Pfdn6 | 14 kDa | 2 | 1 |
| TM50B_MOUSE | Tmem50b | 18 kDa | 0 | 1 |
| SC22B_MOUSE | Sec22b | 25 kDa | 9 | 1 |
| RTN4_MOUSE | Rtn4 | 127 kDa | 19 | 1 |
| GBG11_MOUSE | Gng11 | 8 kDa | 0 | 1 |
| NUCKS_MOUSE | Nucks1 | 26 kDa | 1 | 1 |
| CDN2C_MOUSE | Cdkn2c | 18 kDa | 0 | 1 |
| UB2G2_MOUSE | Ube2g2 | 19 kDa | 3 | 1 |
| P5I11_MOUSE | Trp53i11 | 21 kDa | 6 | 1 |
| S10AA_MOUSE | S100a10 | 11 kDa | 1 | 1 |
| PHB_MOUSE | Phb | 30 kDa | 0 | 1 |
| DJC24_MOUSE | Dnajc24 | 17 kDa | 2 | 1 |
| LSM4_MOUSE | Lsm4 | 15 kDa | 3 | 1 |
| APT_MOUSE | Aprt | 20 kDa | 8 | 1 |
| VMA21_MOUSE | Vma21 | 11 kDa | 3 | 1 |
| CLD5_MOUSE | Cldn5 | 23 kDa | 2 | 1 |
| CT027_MOUSE |  | 19 kDa | 7 | 1 |
| LSM5_MOUSE | Lsm5 | 10 kDa | 2 | 1 |
| S10AE_MOUSE | S100a14 | 12 kDa | 2 | 1 |
| KPYM_MOUSE | Pkm | 58 kDa | 0 | 1 |
| NU4LM_MOUSE | Mtnd4l | 11 kDa | 0 | 1 |
| ARHL1_MOUSE | Adprhl1 | 40 kDa | 0 | 1 |
| CATB_MOUSE | Ctsb | 37 kDa | 0 | 1 |
| SF3B6_MOUSE | Sf3b6 | 15 kDa | 3 | 1 |
| MYH9_MOUSE | Myh9 | 226 kDa | 0 | 1 |
| LAP2B_MOUSE | Tmpo | 50 kDa | 0 | 1 |
| SDHF2_MOUSE | Sdhaf2 | 19 kDa | 1 | 1 |
| EMRE_MOUSE | Smdt1 | 12 kDa | 7 | 1 |
| ASAH1_MOUSE | Asah1 | 45 kDa | 6 | 1 |
| BET1L_MOUSE | Bet1l | 12 kDa | 4 | 1 |
| SMIM8_MOUSE | Smim8 | 11 kDa | 2 | 1 |
| EFTU_MOUSE | Tufm | 50 kDa | 2 | 1 |
| DPM3_MOUSE | Dpm3 | 10 kDa | 1 | 1 |
| PRRT2_MOUSE | Prrt2 | 36 kDa | 1 | 1 |
| F241B_MOUSE | Fam241b | 13 kDa | 1 | 1 |
| KRT82_MOUSE | Krt82 | 57 kDa | 0 | 1 |
| SERF2_MOUSE | Serf2 | 7 kDa | 0 | 1 |
| CRIP1_MOUSE | Crip1 | 9 kDa | 0 | 1 |
| ESYT2_MOUSE | Esyt2 | 94 kDa | 0 | 1 |
| ETFD_MOUSE | Etfdh | 68 kDa | 0 | 1 |
| PREY_MOUSE | Pyurf | 13 kDa | 0 | 1 |
| SI11A_MOUSE | Smim11a | 6 kDa | 0 | 1 |
| SIM15_MOUSE | Smim15 | 9 kDa | 0 | 1 |
| FKBP2_MOUSE | Fkbp2 | 15 kDa | 9 | 1 |
| MMGT1_MOUSE | Mmgt1 | 15 kDa | 6 | 1 |
| SELT_MOUSE | Selenot | 22 kDa | 3 | 1 |
| RM53_MOUSE | Mrpl53 | 13 kDa | 2 | 1 |
| TM160_MOUSE | Tmem160 | 20 kDa | 2 | 1 |
| NH2L1_MOUSE | Snu13 | 14 kDa | 2 | 1 |
| CNIH4_MOUSE | Cnih4 | 16 kDa | 1 | 1 |
| IPKG_MOUSE | Pkig | 8 kDa | 1 | 1 |
| KTAP2_MOUSE | Krtcap2 | 15 kDa | 1 | 1 |
| MTLN_MOUSE | Mtln | 7 kDa | 0 | 1 |
| AKIP_MOUSE | Aurkaip1 | 23 kDa | 0 | 1 |
| CS012_MOUSE |  | 15 kDa | 0 | 1 |
| SELH_MOUSE | Selenoh | 13 kDa | 0 | 1 |
| SH3K1_MOUSE | Sh3kbp1 | 78 kDa | 0 | 1 |
| FABPL_MOUSE | Fabp1 | 14 kDa | 0 | 1 |
| GLRX2_MOUSE | Glrx2 | 17 kDa | 0 | 1 |
| HNRPM_MOUSE | Hnrnpm | 78 kDa | 0 | 1 |
| LSME1_MOUSE | Lsmem1 | 14 kDa | 0 | 1 |
| OBRG_MOUSE | Leprot | 14 kDa | 0 | 1 |
| RT17_MOUSE | Mrps17 | 13 kDa | 0 | 1 |
| LSM3_MOUSE | Lsm3 | 12 kDa | 3 | 1 |
| FXYD6_MOUSE | Fxyd6 | 10 kDa | 8 | 1 |
| GLTP_MOUSE | Gltp | 24 kDa | 17 | 1 |
| VPS25_MOUSE | Vps25 | 21 kDa | 8 | 1 |
| DCTN5_MOUSE | Dctn5 | 20 kDa | 6 | 1 |
| HYPK_MOUSE | Hypk | 15 kDa | 5 | 1 |
| PTMS_MOUSE | Ptms | 11 kDa | 4 | 1 |
| RB11A_MOUSE (+1) | Rab11a | 24 kDa | 4 | 1 |
| VKORL_MOUSE | Vkorc1l1 | 20 kDa | 4 | 1 |
| PEBP1_MOUSE | Pebp1 | 21 kDa | 3 | 1 |
| RIFK_MOUSE | Rfk | 17 kDa | 3 | 1 |
| RNF11_MOUSE | Rnf11 | 17 kDa | 2 | 1 |
| SRP19_MOUSE | Srp19 | 16 kDa | 2 | 1 |
| IF4H_MOUSE | Eif4h | 27 kDa | 2 | 1 |
| CYBC1_MOUSE | Cybc1 | 21 kDa | 2 | 1 |
| ALG14_MOUSE | Alg14 | 24 kDa | 1 | 1 |
| CD59A_MOUSE | Cd59a | 14 kDa | 1 | 1 |
| CYTM1_MOUSE | Cystm1 | 11 kDa | 1 | 1 |
| CNN3_MOUSE | Cnn3 | 36 kDa | 1 | 1 |
| TIM21_MOUSE | Timm21 | 28 kDa | 1 | 1 |
| 2AAA_MOUSE | Ppp2r1a | 65 kDa | 1 | 1 |
| FUMH_MOUSE | Fh | 54 kDa | 0 | 1 |
| CL065_MOUSE |  | 21 kDa | 0 | 1 |
| CFA69_MOUSE | Cfap69 | 106 kDa | 0 | 1 |
| CKLF6_MOUSE | Cmtm6 | 20 kDa | 0 | 1 |
| CLM9_MOUSE | Cd300lg | 37 kDa | 0 | 1 |
| COFA1_MOUSE | Col15a1 | 140 kDa | 0 | 1 |
| DECR_MOUSE | Decr1 | 36 kDa | 0 | 1 |
| DNLZ_MOUSE | Dnlz | 19 kDa | 0 | 1 |
| HSPB1_MOUSE | Hspb1 | 23 kDa | 0 | 1 |
| LYPA1_MOUSE | Lypla1 | 25 kDa | 0 | 1 |
| MPPA_MOUSE | Pmpca | 58 kDa | 0 | 1 |
| NDUBA_MOUSE | Ndufb10 | 21 kDa | 0 | 1 |
| NIPS2_MOUSE | Nipsnap2 | 33 kDa | 0 | 1 |
| PDLI3_MOUSE | Pdlim3 | 34 kDa | 0 | 1 |
| TFIP8_MOUSE | Tnfaip8 | 23 kDa | 0 | 1 |
| UXT_MOUSE | Uxt | 18 kDa | 0 | 1 |
| TMA7_MOUSE | Tma7 | 7 kDa | 0 | 1 |
| ASAP2_MOUSE | Asap2 | 107 kDa | 0 | 1 |
| ASPH_MOUSE | Asph | 83 kDa | 0 | 1 |
| LYRM2_MOUSE | Lyrm2 | 10 kDa | 3 | 1 |
| MTAP2_MOUSE | Map2 | 199 kDa | 15 | 1 |
| PLF4_MOUSE | Pf4 | 11 kDa | 0 | 1 |
| LEG9_MOUSE | Lgals9 | 40 kDa | 0 | 1 |
| NDUV1_MOUSE | Ndufv1 | 51 kDa | 0 | 1 |
| BIP_MOUSE | Hspa5 | 72 kDa | 0 | 1 |
| T22D1_MOUSE | Tsc22d1 | 110 kDa | 2 | 1 |
| F210B_MOUSE | Fam210b | 20 kDa | 1 | 1 |
| GRIK2_MOUSE | Grik2 | 102 kDa | 0 | 1 |
| LSMD1_MOUSE | Naa38 | 13 kDa | 2 | 1 |
| AP3S1_MOUSE | Ap3s1 | 22 kDa | 12 | 1 |
| ECH1_MOUSE | Ech1 | 36 kDa | 0 | 1 |
| TNNT2_MOUSE | Tnnt2 | 36 kDa | 0 | 1 |
| CY1_MOUSE | Cyc1 | 35 kDa | 0 | 1 |
| VISL1_MOUSE | Vsnl1 | 22 kDa | 56 | 0 |
| TBB2A_MOUSE | Tubb2a | 50 kDa | 31 | 0 |
| TBB5_MOUSE | Tubb5 | 50 kDa | 28 | 0 |
| TBB4A_MOUSE | Tubb4a | 50 kDa | 19 | 0 |
| TBB2B_MOUSE | Tubb2b | 50 kDa | 7 | 0 |
| TBB4B_MOUSE | Tubb4b | 50 kDa | 4 | 0 |
| RTN1_MOUSE | Rtn1 | 84 kDa | 40 | 0 |
| SNP25_MOUSE | Snap25 | 23 kDa | 37 | 0 |
| VATG2_MOUSE | Atp6v1g2 | 14 kDa | 35 | 0 |
| HPCL4_MOUSE | Hpcal4 | 22 kDa | 31 | 0 |
| MYPR_MOUSE | Plp1 | 30 kDa | 29 | 0 |
| CPLX1_MOUSE | Cplx1 | 15 kDa | 25 | 0 |
| TBB3_MOUSE | Tubb3 | 50 kDa | 25 | 0 |
| RAP2B_MOUSE | Rap2b | 21 kDa | 13 | 0 |
| RAP2A_MOUSE | Rap2a | 21 kDa | 11 | 0 |
| RAP2C_MOUSE | Rap2c | 21 kDa | 8 | 0 |
| FABP7_MOUSE | Fabp7 | 15 kDa | 22 | 0 |
| TAU_MOUSE (+1) | Mapt | 76 kDa | 22 | 0 |
| S100B_MOUSE | S100b | 11 kDa | 20 | 0 |
| GBG3_MOUSE | Gng3 | 8 kDa | 17 | 0 |
| CPLX2_MOUSE | Cplx2 | 15 kDa | 17 | 0 |
| DNM1L_MOUSE | Dnm1l | 83 kDa | 14 | 0 |
| SOMA_MOUSE | Gh1 | 25 kDa | 13 | 0 |
| TAGL3_MOUSE | Tagln3 | 22 kDa | 13 | 0 |
| GHC2_MOUSE | Slc25a18 | 34 kDa | 13 | 0 |
| MARE3_MOUSE | Mapre3 | 32 kDa | 12 | 0 |
| MAP6_MOUSE | Map6 | 96 kDa | 11 | 0 |
| ARFG1_MOUSE | Arfgap1 | 45 kDa | 11 | 0 |
| GHC1_MOUSE | Slc25a22 | 35 kDa | 11 | 0 |
| CNRP1_MOUSE | Cnrip1 | 19 kDa | 10 | 0 |
| OMP_MOUSE | Omp | 19 kDa | 10 | 0 |
| RAB31_MOUSE | Rab31 | 21 kDa | 10 | 0 |
| GBG7_MOUSE | Gng7 | 7 kDa | 9 | 0 |
| GPM6A_MOUSE | Gpm6a | 31 kDa | 9 | 0 |
| CKLF5_MOUSE | Cmtm5 | 17 kDa | 8 | 0 |
| RAB3A_MOUSE | Rab3a | 25 kDa | 4 | 0 |
| RAB14_MOUSE | Rab14 | 24 kDa | 3 | 0 |
| RAB4B_MOUSE | Rab4b | 24 kDa | 2 | 0 |
| RAB1B_MOUSE | Rab1b | 22 kDa | 2 | 0 |
| GBRL2_MOUSE | Gabarapl2 | 14 kDa | 8 | 0 |
| REEP1_MOUSE | Reep1 | 22 kDa | 8 | 0 |
| PCBP1_MOUSE | Pcbp1 | 37 kDa | 5 | 0 |
| PCBP2_MOUSE | Pcbp2 | 38 kDa | 2 | 0 |
| PCBP3_MOUSE | Pcbp3 | 39 kDa | 1 | 0 |
| GBRL1_MOUSE | Gabarapl1 | 14 kDa | 7 | 0 |
| GBRAP_MOUSE | Gabarap | 14 kDa | 3 | 0 |
| LY6H_MOUSE | Ly6h | 15 kDa | 8 | 0 |
| TM1L2_MOUSE | Tom1l2 | 56 kDa | 8 | 0 |
| OCAD2_MOUSE | Ociad2 | 17 kDa | 8 | 0 |
| HPCA_MOUSE | Hpca | 22 kDa | 8 | 0 |
| MLP3A_MOUSE | Map1lc3a | 14 kDa | 8 | 0 |
| LASP1_MOUSE | Lasp1 | 30 kDa | 7 | 0 |
| E41L1_MOUSE | Epb41l1 | 98 kDa | 7 | 0 |
| PCSK1_MOUSE | Pcsk1n | 27 kDa | 7 | 0 |
| PRAF2_MOUSE | Praf2 | 19 kDa | 7 | 0 |
| RP3A_MOUSE | Rph3a | 75 kDa | 7 | 0 |
| MZT2_MOUSE | Mzt2 | 17 kDa | 7 | 0 |
| SNG3_MOUSE | Syngr3 | 25 kDa | 7 | 0 |
| SRC8_MOUSE | Cttn | 61 kDa | 7 | 0 |
| SYN1_MOUSE | Syn1 | 74 kDa | 5 | 0 |
| SYN2_MOUSE | Syn2 | 63 kDa | 2 | 0 |
| NCALD_MOUSE | Ncald | 22 kDa | 7 | 0 |
| NPTXR_MOUSE | Nptxr | 52 kDa | 7 | 0 |
| MLP3B_MOUSE | Map1lc3b | 15 kDa | 7 | 0 |
| STUM_MOUSE | Stum | 15 kDa | 6 | 0 |
| TP4A2_MOUSE | Ptp4a2 | 19 kDa | 6 | 0 |
| S12A5_MOUSE | Slc12a5 | 126 kDa | 6 | 0 |
| DCLK1_MOUSE | Dclk1 | 84 kDa | 6 | 0 |
| NSF_MOUSE | Nsf | 83 kDa | 6 | 0 |
| VAMP4_MOUSE | Vamp4 | 16 kDa | 6 | 0 |
| AHSA1_MOUSE | Ahsa1 | 38 kDa | 6 | 0 |
| SC11C_MOUSE | Sec11c | 22 kDa | 6 | 0 |
| ARL3_MOUSE | Arl3 | 20 kDa | 6 | 0 |
| CDD_MOUSE | Cda | 16 kDa | 6 | 0 |
| GAL3A_MOUSE | Gatd3a | 28 kDa | 6 | 0 |
| SARNP_MOUSE | Sarnp | 24 kDa | 6 | 0 |
| CK2N2_MOUSE | Camk2n2 | 9 kDa | 4 | 0 |
| CK2N1_MOUSE | Camk2n1 | 9 kDa | 2 | 0 |
| HXK1_MOUSE | Hk1 | 108 kDa | 6 | 0 |
| DPYL2_MOUSE | Dpysl2 | 62 kDa | 6 | 0 |
| MAL2_MOUSE | Mal2 | 19 kDa | 6 | 0 |
| KCRB_MOUSE | Ckb | 43 kDa | 6 | 0 |
| PPR1B_MOUSE | Ppp1r1b | 22 kDa | 5 | 0 |
| PTN_MOUSE | Ptn | 19 kDa | 5 | 0 |
| SH3L2_MOUSE | Sh3bgrl2 | 12 kDa | 5 | 0 |
| CLD10_MOUSE | Cldn10 | 25 kDa | 5 | 0 |
| GOGA7_MOUSE | Golga7 | 16 kDa | 5 | 0 |
| RL22L_MOUSE | Rpl22l1 | 14 kDa | 5 | 0 |
| COMD3_MOUSE | Commd3 | 22 kDa | 5 | 0 |
| DNAL1_MOUSE | Dnal1 | 21 kDa | 5 | 0 |
| DYLT3_MOUSE | Dynlt3 | 13 kDa | 5 | 0 |
| RAB7A_MOUSE | Rab7a | 23 kDa | 5 | 0 |
| RER1_MOUSE | Rer1 | 23 kDa | 5 | 0 |
| SAT2_MOUSE | Sat2 | 19 kDa | 5 | 0 |
| CLCA_MOUSE | Clta | 26 kDa | 5 | 0 |
| E41L3_MOUSE | Epb41l3 | 103 kDa | 5 | 0 |
| MORN4_MOUSE | Morn4 | 16 kDa | 5 | 0 |
| PLD3A_MOUSE | Prelid3a | 19 kDa | 5 | 0 |
| TUSC2_MOUSE | Tusc2 | 12 kDa | 5 | 0 |
| GBG4_MOUSE | Gng4 | 8 kDa | 5 | 0 |
| PCP2_MOUSE | Pcp2 | 13 kDa | 5 | 0 |
| BCAS1_MOUSE | Bcas1 | 67 kDa | 5 | 0 |
| ATG3_MOUSE | Atg3 | 36 kDa | 5 | 0 |
| EAA2_MOUSE | Slc1a2 | 62 kDa | 5 | 0 |
| TPPC2_MOUSE | Trappc2 | 16 kDa | 5 | 0 |
| AAK1_MOUSE | Aak1 | 103 kDa | 5 | 0 |
| MA6D1_MOUSE | Map6d1 | 20 kDa | 5 | 0 |
| STXB1_MOUSE | Stxbp1 | 68 kDa | 5 | 0 |
| MFF_MOUSE | Mff | 33 kDa | 5 | 0 |
| AT1A3_MOUSE | Atp1a3 | 112 kDa | 5 | 0 |
| TPPP_MOUSE | Tppp | 24 kDa | 5 | 0 |
| PTBP2_MOUSE | Ptbp2 | 57 kDa | 4 | 0 |
| GOG7B_MOUSE | GOLGA7B | 18 kDa | 4 | 0 |
| PSB2_MOUSE | Psmb2 | 23 kDa | 4 | 0 |
| CETN2_MOUSE | Cetn2 | 20 kDa | 4 | 0 |
| LIGO1_MOUSE | Lingo1 | 69 kDa | 4 | 0 |
| LYRIC_MOUSE | Mtdh | 64 kDa | 4 | 0 |
| ORML3_MOUSE | Ormdl3 | 17 kDa | 4 | 0 |
| TMCC1_MOUSE | Tmcc1 | 72 kDa | 4 | 0 |
| B2CL2_MOUSE | Bcl2l2 | 21 kDa | 4 | 0 |
| NAT14_MOUSE | Nat14 | 22 kDa | 4 | 0 |
| PHS_MOUSE | Pcbd1 | 12 kDa | 4 | 0 |
| SELM_MOUSE | Selenom | 16 kDa | 4 | 0 |
| TM199_MOUSE | Tmem199 | 23 kDa | 4 | 0 |
| TTC9C_MOUSE | Ttc9c | 20 kDa | 4 | 0 |
| FUCM_MOUSE | Fuom | 17 kDa | 4 | 0 |
| TMEDA_MOUSE | Tmed10 | 25 kDa | 4 | 0 |
| AT1A2_MOUSE | Atp1a2 | 112 kDa | 4 | 0 |
| DNJC5_MOUSE | Dnajc5 | 22 kDa | 4 | 0 |
| NCAM1_MOUSE | Ncam1 | 119 kDa | 4 | 0 |
| 7B2_MOUSE | Scg5 | 24 kDa | 4 | 0 |
| EAA1_MOUSE | Slc1a3 | 60 kDa | 4 | 0 |
| GRIN1_MOUSE | Gprin1 | 95 kDa | 4 | 0 |
| AR6P1_MOUSE | Arl6ip1 | 23 kDa | 4 | 0 |
| MAP1B_MOUSE | Map1b | 270 kDa | 4 | 0 |
| CH60_MOUSE | Hspd1 | 61 kDa | 4 | 0 |
| GBG13_MOUSE | Gng13 | 8 kDa | 4 | 0 |
| EPN1_MOUSE | Epn1 | 60 kDa | 4 | 0 |
| CLAP2_MOUSE | Clasp2 | 141 kDa | 4 | 0 |
| CSRP1_MOUSE | Csrp1 | 21 kDa | 4 | 0 |
| CBLN1_MOUSE | Cbln1 | 21 kDa | 4 | 0 |
| NSF1C_MOUSE | Nsfl1c | 41 kDa | 4 | 0 |
| NRN1_MOUSE | Nrn1 | 15 kDa | 4 | 0 |
| CUTA_MOUSE | Cuta | 19 kDa | 3 | 0 |
| MCRI1_MOUSE | Mcrip1 | 11 kDa | 3 | 0 |
| MPU1_MOUSE | Mpdu1 | 26 kDa | 3 | 0 |
| PHF24_MOUSE | Phf24 | 45 kDa | 3 | 0 |
| DREB_MOUSE | Dbn1 | 77 kDa | 3 | 0 |
| PSB6_MOUSE | Psmb6 | 25 kDa | 3 | 0 |
| RPB9_MOUSE | Polr2i | 15 kDa | 3 | 0 |
| RGS8_MOUSE | Rgs8 | 21 kDa | 3 | 0 |
| DESI1_MOUSE | Desi1 | 18 kDa | 3 | 0 |
| VPP1_MOUSE | Atp6v0a1 | 96 kDa | 3 | 0 |
| TWF2_MOUSE | Twf2 | 39 kDa | 3 | 0 |
| DTD2_MOUSE | Dtd2 | 18 kDa | 3 | 0 |
| THY1_MOUSE | Thy1 | 18 kDa | 3 | 0 |
| DBNL_MOUSE | Dbnl | 49 kDa | 3 | 0 |
| AKA7A_MOUSE (+1) | Akap7 | 9 kDa | 3 | 0 |
| GGACT_MOUSE | Ggact | 17 kDa | 3 | 0 |
| NGB_MOUSE | Ngb | 17 kDa | 3 | 0 |
| PP14A_MOUSE | Ppp1r14a | 17 kDa | 3 | 0 |
| IFT27_MOUSE | Ift27 | 21 kDa | 3 | 0 |
| WDR37_MOUSE | Wdr37 | 55 kDa | 3 | 0 |
| TSTD3_MOUSE | Tstd3 | 17 kDa | 3 | 0 |
| SUMO1_MOUSE (+1) | Sumo1 | 12 kDa | 3 | 0 |
| RABP1_MOUSE | Crabp1 | 16 kDa | 3 | 0 |
| SMAP1_MOUSE | Smap1 | 48 kDa | 3 | 0 |
| NDUF2_MOUSE | Ndufaf2 | 20 kDa | 3 | 0 |
| ERD22_MOUSE | Kdelr2 | 24 kDa | 3 | 0 |
| PLBL2_MOUSE | Plbd2 | 66 kDa | 3 | 0 |
| THEM6_MOUSE | Them6 | 24 kDa | 3 | 0 |
| LYAG_MOUSE | Gaa | 106 kDa | 3 | 0 |
| RHOQ_MOUSE | Rhoq | 23 kDa | 3 | 0 |
| SAR1A_MOUSE | Sar1a | 22 kDa | 3 | 0 |
| PLLP_MOUSE | Pllp | 20 kDa | 3 | 0 |
| ROA0_MOUSE | Hnrnpa0 | 31 kDa | 3 | 0 |
| CLD11_MOUSE | Cldn11 | 22 kDa | 3 | 0 |
| DPY30_MOUSE | Dpy30 | 11 kDa | 3 | 0 |
| TM147_MOUSE | Tmem147 | 25 kDa | 3 | 0 |
| CZIB_MOUSE | Czib | 18 kDa | 3 | 0 |
| GDIR1_MOUSE | Arhgdia | 23 kDa | 3 | 0 |
| MAP1A_MOUSE | Map1a | 300 kDa | 3 | 0 |
| HIKES_MOUSE | Hikeshi | 22 kDa | 3 | 0 |
| RPB4_MOUSE | Polr2d | 16 kDa | 3 | 0 |
| RS30_MOUSE | Fau | 7 kDa | 3 | 0 |
| VATB2_MOUSE | Atp6v1b2 | 57 kDa | 3 | 0 |
| EFHD2_MOUSE | Efhd2 | 27 kDa | 3 | 0 |
| TIM44_MOUSE | Timm44 | 51 kDa | 3 | 0 |
| E41L2_MOUSE | Epb41l2 | 110 kDa | 3 | 0 |
| TPPP3_MOUSE | Tppp3 | 19 kDa | 3 | 0 |
| HNRPK_MOUSE | Hnrnpk | 51 kDa | 3 | 0 |
| SMAKA_MOUSE |  | 12 kDa | 3 | 0 |
| NECA2_MOUSE | Necab2 | 43 kDa | 3 | 0 |
| LSM7_MOUSE | Lsm7 | 12 kDa | 3 | 0 |
| PENK_MOUSE | Penk | 31 kDa | 3 | 0 |
| ADDG_MOUSE | Add3 | 79 kDa | 3 | 0 |
| PPIH_MOUSE | Ppih | 20 kDa | 3 | 0 |
| PALM_MOUSE | Palm | 42 kDa | 3 | 0 |
| NCBP2_MOUSE | Ncbp2 | 18 kDa | 3 | 0 |
| LIMD2_MOUSE | Limd2 | 14 kDa | 3 | 0 |
| LYPD1_MOUSE | Lypd1 | 15 kDa | 3 | 0 |
| KTNB1_MOUSE | Katnb1 | 73 kDa | 2 | 0 |
| COX42_MOUSE | Cox4i2 | 20 kDa | 2 | 0 |
| TM263_MOUSE | Tmem263 | 12 kDa | 2 | 0 |
| AGFG1_MOUSE | Agfg1 | 58 kDa | 2 | 0 |
| TM222_MOUSE | Tmem222 | 23 kDa | 2 | 0 |
| UCHL1_MOUSE | Uchl1 | 25 kDa | 2 | 0 |
| ARC1A_MOUSE | Arpc1a | 42 kDa | 2 | 0 |
| UBL5_MOUSE | Ubl5 | 9 kDa | 2 | 0 |
| GAPR1_MOUSE | Glipr2 | 17 kDa | 2 | 0 |
| EMC6_MOUSE | Emc6 | 12 kDa | 2 | 0 |
| SCAM5_MOUSE | Scamp5 | 26 kDa | 2 | 0 |
| SF3B5_MOUSE | Sf3b5 | 10 kDa | 2 | 0 |
| ABI2_MOUSE | Abi2 | 49 kDa | 2 | 0 |
| PDZ11_MOUSE | Pdzd11 | 16 kDa | 2 | 0 |
| PC4L1_MOUSE | Pcp4l1 | 8 kDa | 2 | 0 |
| STAM1_MOUSE | Stam | 60 kDa | 2 | 0 |
| GEMI7_MOUSE | Gemin7 | 14 kDa | 2 | 0 |
| LTOR4_MOUSE | Lamtor4 | 11 kDa | 2 | 0 |
| PLPR4_MOUSE | Plppr4 | 83 kDa | 2 | 0 |
| ARL5A_MOUSE (+1) | Arl5a | 21 kDa | 2 | 0 |
| CAMP_MOUSE | Camp | 19 kDa | 2 | 0 |
| COMD6_MOUSE | Commd6 | 10 kDa | 2 | 0 |
| DEMA_MOUSE | Dmtn | 45 kDa | 2 | 0 |
| EF1G_MOUSE | Eef1g | 50 kDa | 2 | 0 |
| EMD_MOUSE | Emd | 29 kDa | 2 | 0 |
| ERG28_MOUSE | Erg28 | 16 kDa | 2 | 0 |
| GPM6B_MOUSE | Gpm6b | 36 kDa | 2 | 0 |
| KAZRN_MOUSE | Kazn | 87 kDa | 2 | 0 |
| LYNX1_MOUSE | Lynx1 | 13 kDa | 2 | 0 |
| MAG_MOUSE | Mag | 69 kDa | 2 | 0 |
| NEUM_MOUSE | Gap43 | 24 kDa | 2 | 0 |
| SE6L2_MOUSE | Sez6l2 | 98 kDa | 2 | 0 |
| STX7_MOUSE | Stx7 | 30 kDa | 2 | 0 |
| T22D4_MOUSE | Tsc22d4 | 40 kDa | 2 | 0 |
| TSN2_MOUSE | Tspan2 | 24 kDa | 2 | 0 |
| WDR13_MOUSE | Wdr13 | 54 kDa | 2 | 0 |
| ABI1_MOUSE | Abi1 | 52 kDa | 2 | 0 |
| CLCB_MOUSE | Cltb | 25 kDa | 2 | 0 |
| DOPP1_MOUSE | Dolpp1 | 27 kDa | 2 | 0 |
| CAMLG_MOUSE | Camlg | 33 kDa | 2 | 0 |
| LEGL_MOUSE | Lgalsl | 19 kDa | 2 | 0 |
| BL1S2_MOUSE | Bloc1s2 | 16 kDa | 2 | 0 |
| TM14A_MOUSE | Tmem14a | 11 kDa | 2 | 0 |
| AR2BP_MOUSE | Arl2bp | 19 kDa | 2 | 0 |
| SC31A_MOUSE | Sec31a | 134 kDa | 2 | 0 |
| LBH_MOUSE | Lbh | 12 kDa | 2 | 0 |
| CYTSB_MOUSE | Specc1 | 118 kDa | 2 | 0 |
| SERC1_MOUSE | Serinc1 | 51 kDa | 2 | 0 |
| FCERG_MOUSE | Fcer1g | 10 kDa | 2 | 0 |
| ARHG7_MOUSE | Arhgef7 | 97 kDa | 2 | 0 |
| DJC15_MOUSE | Dnajc15 | 16 kDa | 2 | 0 |
| IFT25_MOUSE | Hspb11 | 16 kDa | 2 | 0 |
| PGRC2_MOUSE | Pgrmc2 | 23 kDa | 2 | 0 |
| LHPL4_MOUSE | Lhfpl4 | 27 kDa | 2 | 0 |
| GLNA_MOUSE | Glul | 42 kDa | 2 | 0 |
| NECP1_MOUSE | Necap1 | 30 kDa | 2 | 0 |
| UBL3_MOUSE | Ubl3 | 13 kDa | 2 | 0 |
| NC2B_MOUSE | Dr1 | 19 kDa | 2 | 0 |
| SPRE1_MOUSE | Spred1 | 51 kDa | 2 | 0 |
| PHF5A_MOUSE | Phf5a | 12 kDa | 2 | 0 |
| BORC8_MOUSE | Borcs8 | 14 kDa | 2 | 0 |
| PCP4_MOUSE | Pcp4 | 7 kDa | 2 | 0 |
| RT16_MOUSE | Mrps16 | 15 kDa | 2 | 0 |
| CTTB2_MOUSE | Cttnbp2 | 179 kDa | 2 | 0 |
| NINJ1_MOUSE | Ninj1 | 17 kDa | 2 | 0 |
| CA2D3_MOUSE | Cacna2d3 | 123 kDa | 2 | 0 |
| SPAST_MOUSE | Spast | 66 kDa | 2 | 0 |
| PSB9_MOUSE (+1) | Psmb9 | 23 kDa | 2 | 0 |
| BAALC_MOUSE | Baalc | 16 kDa | 2 | 0 |
| VKOR1_MOUSE | Vkorc1 | 18 kDa | 2 | 0 |
| TWF1_MOUSE | Twf1 | 40 kDa | 2 | 0 |
| HEXB_MOUSE | Hexb | 61 kDa | 2 | 0 |
| DUS26_MOUSE | Dusp26 | 24 kDa | 2 | 0 |
| RISC_MOUSE | Scpep1 | 51 kDa | 2 | 0 |
| RNF5_MOUSE | Rnf5 | 20 kDa | 2 | 0 |
| WRB_MOUSE | Wrb | 20 kDa | 2 | 0 |
| MOC2A_MOUSE | Mocs2 | 10 kDa | 2 | 0 |
| GRDN_MOUSE | Ccdc88a | 216 kDa | 2 | 0 |
| PSB8_MOUSE | Psmb8 | 30 kDa | 2 | 0 |
| PKHB1_MOUSE | Plekhb1 | 27 kDa | 2 | 0 |
| CC167_MOUSE | Ccdc167 | 11 kDa | 2 | 0 |
| CHCH1_MOUSE | Chchd1 | 14 kDa | 2 | 0 |
| PHLA3_MOUSE | Phlda3 | 14 kDa | 2 | 0 |
| MTND_MOUSE | Adi1 | 22 kDa | 2 | 0 |
| PLD3_MOUSE | Pld3 | 54 kDa | 2 | 0 |
| REEP6_MOUSE | Reep6 | 22 kDa | 2 | 0 |
| PCNP_MOUSE | Pcnp | 19 kDa | 2 | 0 |
| C2C4C_MOUSE | C2cd4cC2CD4 | 45 kDa | 2 | 0 |
| C42S2_MOUSE | Cdc42se2 | 9 kDa | 2 | 0 |
| BIN1_MOUSE | Bin1 | 64 kDa | 2 | 0 |
| HS90A_MOUSE | Hsp90aa1 | 85 kDa | 2 | 0 |
| FA49B_MOUSE | Fam49b | 37 kDa | 2 | 0 |
| URM1_MOUSE | Urm1 | 11 kDa | 2 | 0 |
| C2C2L_MOUSE | C2cd2l | 76 kDa | 2 | 0 |
| FAT3_MOUSE | Fat3 | 502 kDa | 2 | 0 |
| sp\|Q6ZPJ3\|UBE2O_MOUSE | Ube2o | 141 kDa | 2 | 0 |
| MIC60_MOUSE | Immt | 84 kDa | 2 | 0 |
| RL39_MOUSE | Rpl39 | 6 kDa | 2 | 0 |
| AMPH_MOUSE | Amph | 75 kDa | 2 | 0 |
| SYT12_MOUSE | Syt12 | 47 kDa | 2 | 0 |
| SYT7_MOUSE | Syt7 | 45 kDa | 2 | 0 |
| UBP5_MOUSE | Usp5 | 96 kDa | 2 | 0 |
| MTURN_MOUSE | Mturn | 15 kDa | 2 | 0 |
| PRIO_MOUSE | Prnp | 28 kDa | 2 | 0 |
| RFA3_MOUSE | Rpa3 | 14 kDa | 2 | 0 |
| TR112_MOUSE | Trmt112 | 14 kDa | 2 | 0 |
| PSB5_MOUSE | Psmb5 | 29 kDa | 1 | 0 |
| CE170_MOUSE | Cep170 | 175 kDa | 1 | 0 |
| TBCB_MOUSE | Tbcb | 27 kDa | 1 | 0 |
| NUD16_MOUSE | Nudt16 | 22 kDa | 1 | 0 |
| RBBP9_MOUSE | Rbbp9 | 21 kDa | 1 | 0 |
| PMVK_MOUSE | Pmvk | 22 kDa | 1 | 0 |
| KISHA_MOUSE | Tmem167a | 8 kDa | 1 | 0 |
| TOM34_MOUSE | Tomm34 | 34 kDa | 1 | 0 |
| EFR3B_MOUSE | Efr3b | 92 kDa | 1 | 0 |
| DYN1_MOUSE | Dnm1 | 98 kDa | 1 | 0 |
| CN37_MOUSE | Cnp | 47 kDa | 1 | 0 |
| DUS14_MOUSE | Dusp14 | 22 kDa | 1 | 0 |
| OCAD1_MOUSE | Ociad1 | 28 kDa | 1 | 0 |
| SGSM1_MOUSE | Sgsm1 | 123 kDa | 1 | 0 |
| ARGI1_MOUSE | Arg1 | 35 kDa | 1 | 0 |
| OCC1_MOUSE |  | 6 kDa | 1 | 0 |
| CCKN_MOUSE | Cck | 13 kDa | 1 | 0 |
| SGIP1_MOUSE | Sgip1 | 86 kDa | 1 | 0 |
| CEND_MOUSE | Cend1 | 15 kDa | 1 | 0 |
| IFT20_MOUSE | Ift20 | 15 kDa | 1 | 0 |
| AMPL_MOUSE | Lap3 | 56 kDa | 1 | 0 |
| NRX1A_MOUSE (+1) | Nrxn1 | 166 kDa | 1 | 0 |
| AMPD2_MOUSE | Ampd2 | 92 kDa | 1 | 0 |
| AP4S1_MOUSE | Ap4s1 | 17 kDa | 1 | 0 |
| GP158_MOUSE | Gpr158 | 134 kDa | 1 | 0 |
| TSR2_MOUSE | Tsr2 | 21 kDa | 1 | 0 |
| A7L3B_MOUSE | Atxn7l3b | 11 kDa | 1 | 0 |
| RBMX_MOUSE (+1) | Rbmx | 42 kDa | 1 | 0 |
| BUD31_MOUSE | Bud31 | 17 kDa | 1 | 0 |
| STK39_MOUSE | Stk39 | 60 kDa | 1 | 0 |
| CAP2_MOUSE | Cap2 | 53 kDa | 1 | 0 |
| CBLN3_MOUSE | Cbln3 | 21 kDa | 1 | 0 |
| GEMI6_MOUSE | Gemin6 | 19 kDa | 1 | 0 |
| AGRV1_MOUSE | Adgrv1 | 687 kDa | 1 | 0 |
| PLB1_MOUSE | Plb1 | 165 kDa | 1 | 0 |
| RAMAC_MOUSE | Ramac | 15 kDa | 1 | 0 |
| MSS4_MOUSE | Rabif | 14 kDa | 1 | 0 |
| ARI1A_MOUSE | Arid1a | 242 kDa | 1 | 0 |
| CAPS1_MOUSE | Cadps | 153 kDa | 1 | 0 |
| RENR_MOUSE | Atp6ap2 | 39 kDa | 1 | 0 |
| CADM4_MOUSE | Cadm4 | 43 kDa | 1 | 0 |
| SRP09_MOUSE | Srp9 | 10 kDa | 1 | 0 |
| WASF1_MOUSE | Wasf1 | 62 kDa | 1 | 0 |
| ARSB_MOUSE | Arsb | 60 kDa | 1 | 0 |
| CUED2_MOUSE | Cuedc2 | 32 kDa | 1 | 0 |
| GBG8_MOUSE | Gng8 | 8 kDa | 1 | 0 |
| GFRA1_MOUSE | Gfra1 | 52 kDa | 1 | 0 |
| LNEBL_MOUSE | Nebl | 31 kDa | 1 | 0 |
| PPM1E_MOUSE | Ppm1e | 83 kDa | 1 | 0 |
| PT117_MOUSE | Pet117 | 9 kDa | 1 | 0 |
| PTPRN_MOUSE | Ptprn | 106 kDa | 1 | 0 |
| SCOC_MOUSE | Scoc | 14 kDa | 1 | 0 |
| SNP47_MOUSE | Snap47 | 47 kDa | 1 | 0 |
| UFD1_MOUSE | Ufd1 | 34 kDa | 1 | 0 |
| VA0D1_MOUSE | Atp6v0d1 | 40 kDa | 1 | 0 |
| YD286_MOUSE |  | 13 kDa | 1 | 0 |
| HDGR3_MOUSE | Hdgfl3 | 22 kDa | 1 | 0 |
| SEPT9_MOUSE | 09-sept | 66 kDa | 1 | 0 |
| SYT17_MOUSE | Syt17 | 53 kDa | 1 | 0 |
| APLP2_MOUSE | Aplp2 | 80 kDa | 1 | 0 |
| ATRAP_MOUSE | Agtrap | 18 kDa | 1 | 0 |
| BET1_MOUSE | Bet1 | 13 kDa | 1 | 0 |
| CADM3_MOUSE | Cadm3 | 43 kDa | 1 | 0 |
| CD5R1_MOUSE | Cdk5r1 | 34 kDa | 1 | 0 |
| CHCH5_MOUSE | Chchd5 | 12 kDa | 1 | 0 |
| CHUR_MOUSE | Churc1 | 13 kDa | 1 | 0 |
| DD19A_MOUSE | Ddx19a | 54 kDa | 1 | 0 |
| ES8L2_MOUSE | Eps8l2 | 82 kDa | 1 | 0 |
| GOT1B_MOUSE | Golt1b | 15 kDa | 1 | 0 |
| IFG15_MOUSE | Tor1aip2 | 15 kDa | 1 | 0 |
| MAP1S_MOUSE | Map1s | 103 kDa | 1 | 0 |
| MY18A_MOUSE | Myo18a | 233 kDa | 1 | 0 |
| NEPR1_MOUSE | Cnep1r1 | 14 kDa | 1 | 0 |
| NMT1_MOUSE | Nmt1 | 57 kDa | 1 | 0 |
| OSTC_MOUSE | Ostc | 17 kDa | 1 | 0 |
| PRRT1_MOUSE | Prrt1 | 31 kDa | 1 | 0 |
| PSMG3_MOUSE | Psmg3 | 13 kDa | 1 | 0 |
| RPB11_MOUSE | Polr2j | 13 kDa | 1 | 0 |
| SELS_MOUSE | Selenos | 22 kDa | 1 | 0 |
| SIM13_MOUSE | Smim13 | 10 kDa | 1 | 0 |
| SPT4A_MOUSE (+1) | Supt4h1a | 13 kDa | 1 | 0 |
| SRXN1_MOUSE | Srxn1 | 14 kDa | 1 | 0 |
| SWI5_MOUSE | Swi5 | 10 kDa | 1 | 0 |
| SYS1_MOUSE | Sys1 | 18 kDa | 1 | 0 |
| TACC1_MOUSE | Tacc1 | 84 kDa | 1 | 0 |
| TMM42_MOUSE | Tmem42 | 17 kDa | 1 | 0 |
| TMM47_MOUSE | Tmem47 | 20 kDa | 1 | 0 |
| UBP14_MOUSE | Usp14 | 56 kDa | 1 | 0 |
| DHE3_MOUSE | Glud1 | 61 kDa | 1 | 0 |
| MAP4_MOUSE | Map4 | 117 kDa | 1 | 0 |
| WDR7_MOUSE | Wdr7 | 163 kDa | 1 | 0 |
| CTND1_MOUSE | Ctnnd1 | 105 kDa | 1 | 0 |
| LRAD4_MOUSE | Ldlrad4 | 34 kDa | 1 | 0 |
| AIF1_MOUSE | Aif1 | 17 kDa | 1 | 0 |
| BTF3_MOUSE | Btf3 | 22 kDa | 1 | 0 |
| CF203_MOUSE |  | 28 kDa | 1 | 0 |
| KCC2B_MOUSE (+1) | Camk2b | 60 kDa | 1 | 0 |
| LZIC_MOUSE | Lzic | 22 kDa | 1 | 0 |
| PACN1_MOUSE | Pacsin1 | 51 kDa | 1 | 0 |
| TOM1_MOUSE | Tom1 | 54 kDa | 1 | 0 |
| ARAF_MOUSE | Araf | 68 kDa | 1 | 0 |
| ARPC3_MOUSE | Arpc3 | 21 kDa | 1 | 0 |
| ATPF1_MOUSE | Atpaf1 | 36 kDa | 1 | 0 |
| B9D2_MOUSE | B9d2 | 19 kDa | 1 | 0 |
| BORG4_MOUSE | Cdc42ep4 | 38 kDa | 1 | 0 |
| C1QBP_MOUSE | C1qbp | 31 kDa | 1 | 0 |
| C56D2_MOUSE | Cyb561d2 | 24 kDa | 1 | 0 |
| CKLF4_MOUSE | Cmtm4 | 23 kDa | 1 | 0 |
| CLD1_MOUSE | Cldn1 | 23 kDa | 1 | 0 |
| CNTN1_MOUSE | Cntn1 | 113 kDa | 1 | 0 |
| COMD4_MOUSE | Commd4 | 22 kDa | 1 | 0 |
| CPLX3_MOUSE | Cplx3 | 18 kDa | 1 | 0 |
| DCTN2_MOUSE | Dctn2 | 44 kDa | 1 | 0 |
| EVA1A_MOUSE | Eva1a | 18 kDa | 1 | 0 |
| FA49A_MOUSE | Fam49a | 37 kDa | 1 | 0 |
| GSKIP_MOUSE | Gskip | 16 kDa | 1 | 0 |
| ICEF1_MOUSE | Ipcef1 | 46 kDa | 1 | 0 |
| ITM2B_MOUSE | Itm2b | 30 kDa | 1 | 0 |
| MEF2A_MOUSE (+2) | Mef2a | 54 kDa | 1 | 0 |
| MK_MOUSE | Mdk | 15 kDa | 1 | 0 |
| NKAI4_MOUSE | Nkain4 | 23 kDa | 1 | 0 |
| PP2BA_MOUSE | Ppp3ca | 59 kDa | 1 | 0 |
| PSMF1_MOUSE | Psmf1 | 30 kDa | 1 | 0 |
| PTH_MOUSE | Ptrh1 | 22 kDa | 1 | 0 |
| PTPR2_MOUSE | Ptprn2 | 111 kDa | 1 | 0 |
| RASH_MOUSE (+1) | Hras | 21 kDa | 1 | 0 |
| RGS10_MOUSE | Rgs10 | 21 kDa | 1 | 0 |
| RPC9_MOUSE | Crcp | 17 kDa | 1 | 0 |
| SHSA4_MOUSE | Shisa4 | 21 kDa | 1 | 0 |
| SNRPA_MOUSE | Snrpa | 32 kDa | 1 | 0 |
| SPRN_MOUSE | Sprn | 15 kDa | 1 | 0 |
| SYT1_MOUSE | Syt1 | 47 kDa | 1 | 0 |
| TBPL1_MOUSE | Tbpl1 | 21 kDa | 1 | 0 |
| TMX1_MOUSE | Tmx1 | 31 kDa | 1 | 0 |
| TPD52_MOUSE | Tpd52 | 24 kDa | 1 | 0 |
| TS101_MOUSE | Tsg101 | 44 kDa | 1 | 0 |
| VHL_MOUSE | Vhl | 21 kDa | 1 | 0 |
| YIF1B_MOUSE | Yif1b | 34 kDa | 1 | 0 |
| ZNF2_MOUSE | Znf2 | 49 kDa | 1 | 0 |
| CIA2A_MOUSE | Ciao2a | 18 kDa | 1 | 0 |
| STXB6_MOUSE | Stxbp6 | 24 kDa | 1 | 0 |
| YPEL5_MOUSE | Ypel5 | 14 kDa | 1 | 0 |
| PP14B_MOUSE | Ppp1r14b | 16 kDa | 1 | 0 |
| ABH15_MOUSE | Abhd15 | 51 kDa | 1 | 0 |
| EIF2A_MOUSE | Eif2a | 64 kDa | 1 | 0 |
| LNP_MOUSE | Lnpk | 48 kDa | 1 | 0 |
| TRIQK_MOUSE | Triqk | 10 kDa | 1 | 0 |
| TP8L1_MOUSE | Tnfaip8l1 | 21 kDa | 1 | 0 |
| SFT2B_MOUSE | Sft2d2 | 18 kDa | 1 | 0 |
| STMN3_MOUSE | Stmn3 | 21 kDa | 1 | 0 |
| NPTX1_MOUSE | Nptx1 | 47 kDa | 1 | 0 |
| ACINU_MOUSE | Acin1 | 151 kDa | 1 | 0 |
| RNK_MOUSE | Rnasek | 11 kDa | 1 | 0 |
| APOE_MOUSE | Apoe | 36 kDa | 1 | 0 |
| PDYN_MOUSE | Pdyn | 28 kDa | 1 | 0 |
| CDN2D_MOUSE | Cdkn2d | 18 kDa | 1 | 0 |
