# Supplementary material for: Loss of Ecrg4 improves calcium oxalate nephropathy
Source: PLoS One. 2022 Oct 13;17(10):e0275972. doi: 10.1371/journal.pone.0275972 (PMC9560046; doi:10.1371/journal.pone.0275972)
Supplement: S2 Table — (DOCX) [file pone.0275972.s017.docx]

**Supplemental Table 2**. **Identified proteins by mass spectrometry in transfected HEK cells.**

| **Identified proteins** | **Alternate ID** | **Molecular Weight** | **Total spectrum count**  **(±20kDA section)** | **Total spectrum count**  **(±15kDA section)** |
| --- | --- | --- | --- | --- |
| Q32Q12_HUMAN | NME1-NME2 | 33 kDa | 98 | 109 |
| sp\|P62937\|PPIA_HUMAN Peptidyl-prolyl cis-trans isomerase A OS=Homo sapiens GN=PPIA PE=1 SV=2 | | 18 kDa | 158 | 9 |
| P00761 |  | 24 kDa | 19 | 35 |
| MIF_HUMAN | MIF | 12 kDa | 0 | 14 |
| PROF1_HUMAN | PFN1 | 15 kDa | 8 | 136 |
| IF5A1_HUMAN | EIF5A | 17 kDa | 119 | 3 |
| sp\|PRDX1_HUMAN\| (+1) | | 22 kDa | 102 | 0 |
| RS18_HUMAN | RPS18 | 18 kDa | 34 | 32 |
| COX6C_HUMAN | COX6C | 9 kDa | 0 | 1 |
| RS15A_HUMAN | RPS15A | 15 kDa | 5 | 72 |
| sp\|P62979\|RS27A_HUMANUbiquitin-40S ribosomal protein S27a | | ? | 6 | 0 |
| CH10_HUMAN | HSPE1 | 11 kDa | 0 | 3 |
| RL23_HUMAN | RPL23 | 15 kDa | 16 | 57 |
| RS14_HUMAN (+1) | RPS14 | 16 kDa | 42 | 39 |
| ARF1_HUMAN | ARF1 | 21 kDa | 76 | 4 |
| HINT1_HUMAN | HINT1 | 14 kDa | 0 | 41 |
| Q8WVC2_HUMAN (+1) | RPS21 | 9 kDa | 0 | 0 |
| RL23A_HUMAN | RPL23A | 18 kDa | 43 | 10 |
| RL38_HUMAN | RPL38 | 8 kDa | 0 | 1 |
| COX41_HUMAN | COX4I1 | 20 kDa | 0 | 61 |
| COF1_HUMAN | CFL1 | 19 kDa | 68 | 3 |
| RS16_HUMAN | RPS16 | 16 kDa | 33 | 28 |
| TAGL2_HUMAN | TAGLN2 | 22 kDa | 65 | 0 |
| C9JFR7_HUMAN (+2) | CYCS | 11 kDa | 2 | 29 |
| PEBP1_HUMAN | PEBP1 | 21 kDa | 51 | 2 |
| sp\|P09211\|GSTP1_HUMANGlutathione S-transferase P OS=Homo sapiens GN=GSTP1 PE=1 SV=2 | | ? | 66 | 0 |
| TCP4_HUMAN | SUB1 | 14 kDa | 13 | 32 |
| RS7_HUMAN | RPS7 | 22 kDa | 43 | 3 |
| RS19_HUMAN | RPS19 | 16 kDa | 28 | 22 |
| RL11_HUMAN | RPL11 | 20 kDa | 56 | 1 |
| E5RI99_HUMAN (+1) | RPL30 | 13 kDa | 5 | 44 |
| RS13_HUMAN | RPS13 | 17 kDa | 36 | 11 |
| RS11_HUMAN | RPS11 | 18 kDa | 51 | 2 |
| RL35A_HUMAN | RPL35A | 13 kDa | 5 | 26 |
| UBE2N_HUMAN | UBE2N | 17 kDa | 11 | 42 |
| E5RJD8_HUMAN (+1) | TBCA | 14 kDa | 0 | 34 |
| RLA2_HUMAN | RPLP2 | 12 kDa | 3 | 37 |
| H4_HUMAN | HIST1H4A | 11 kDa | 7 | 13 |
| sp\|P10599\|THIO_HUMAN Thioredoxin OS=Homo sapiens GN=TXN PE=1 SV=3 (+1) | | 12 kDa | 0 | 5 |
| PSB5_HUMAN | PSMB5 | 28 kDa | 46 | 1 |
| PDCD5_HUMAN | PDCD5 | 14 kDa | 0 | 19 |
| PPIB_HUMAN | PPIB | 24 kDa | 41 | 0 |
| K7ELC2_HUMAN (+1) | RPS15 | 18 kDa | 44 | 0 |
| RS27_HUMAN | RPS27 | 9 kDa | 1 | 8 |
| PRDX3_HUMAN | PRDX3 | 28 kDa | 46 | 1 |
| SSBP_HUMAN | SSBP1 | 17 kDa | 3 | 40 |
| ACBP_HUMAN | DBI | 10 kDa | 0 | 0 |
| F8W1R7_HUMAN (+2) | MYL6 | 16 kDa | 2 | 40 |
| RS12_HUMAN | RPS12 | 15 kDa | 2 | 29 |
| PRDX2_HUMAN | PRDX2 | 22 kDa | 48 | 1 |
| CALM1_HUMAN (+2) | CALM1 | 17 kDa | 35 | 4 |
| A0A087WUS0_HUMAN (+4) | RPS24 | 15 kDa | 44 | 0 |
| TBA1B_HUMAN | TUBA1B | 50 kDa | 19 | 8 |
| RL37A_HUMAN | RPL37A | 10 kDa | 10 | 8 |
| A0A1W2PQS6_HUMAN | RPS10-NUDT3 | 33 kDa | 28 | 6 |
| ML12B_HUMAN | MYL12B | 20 kDa | 33 | 5 |
| RUXG_HUMAN | SNRPG | 8 kDa | 0 | 0 |
| PSB3_HUMAN | PSMB3 | 23 kDa | 37 | 0 |
| RHOA_HUMAN | RHOA | 22 kDa | 39 | 0 |
| RUXE_HUMAN | SNRPE | 11 kDa | 0 | 6 |
| RL26_HUMAN | RPL26 | 17 kDa | 31 | 1 |
| RL27_HUMAN | RPL27 | 16 kDa | 28 | 7 |
| A0A024R4M0_HUMAN (+1) | RPS9 | 23 kDa | 35 | 0 |
| RS20_HUMAN | RPS20 | 13 kDa | 5 | 29 |
| NLTP_HUMAN | SCP2 | 59 kDa | 0 | 17 |
| PRDX5_HUMAN | PRDX5 | 22 kDa | 19 | 19 |
| SMD2_HUMAN | SNRPD2 | 14 kDa | 1 | 39 |
| ATP5L_HUMAN | ATP5L | 11 kDa | 0 | 7 |
| RL12_HUMAN | RPL12 | 18 kDa | 37 | 0 |
| MGN2_HUMAN | MAGOHB | 17 kDa | 23 | 16 |
| DYL1_HUMAN | DYNLL1 | 10 kDa | 0 | 0 |
| G5E9Q6_HUMAN | PFN2 | 21 kDa | 0 | 16 |
| FKB1A_HUMAN | FKBP1A | 12 kDa | 0 | 3 |
| D3YTB1_HUMAN (+2) | RPL32 | 16 kDa | 27 | 0 |
| NTF2_HUMAN | NUTF2 | 14 kDa | 0 | 2 |
| RAB7A_HUMAN | RAB7A | 23 kDa | 38 | 0 |
| DOPD_HUMAN | DDT | 13 kDa | 0 | 6 |
| M0R0F0_HUMAN (+1) | RPS5 | 22 kDa | 36 | 1 |
| CDN2A_HUMAN (+1) | CDKN2A | 17 kDa | 17 | 22 |
| STMN1_HUMAN | STMN1 | 17 kDa | 25 | 4 |
| RL36_HUMAN | RPL36 | 12 kDa | 3 | 26 |
| COX2_HUMAN | MT-CO2 | 26 kDa | 31 | 3 |
| PIN1_HUMAN | PIN1 | 18 kDa | 27 | 0 |
| IF1AX_HUMAN | EIF1AX | 16 kDa | 12 | 21 |
| PARK7_HUMAN | PARK7 | 20 kDa | 36 | 0 |
| RS29_HUMAN | RPS29 | 7 kDa | 1 | 3 |
| RL18A_HUMAN | RPL18A | 21 kDa | 28 | 0 |
| SMD3_HUMAN | SNRPD3 | 14 kDa | 2 | 27 |
| PIN4_HUMAN | PIN4 | 14 kDa | 2 | 25 |
| RFA3_HUMAN | RPA3 | 14 kDa | 0 | 2 |
| RUXF_HUMAN | SNRPF | 10 kDa | 0 | 0 |
| CX6B1_HUMAN | COX6B1 | 10 kDa | 0 | 0 |
| ELOB_HUMAN | ELOB | 13 kDa | 5 | 25 |
| SRP09_HUMAN | SRP9 | 10 kDa | 0 | 0 |
| RS28_HUMAN | RPS28 | 8 kDa | 0 | 0 |
| PSB2_HUMAN | PSMB2 | 23 kDa | 19 | 2 |
| H3BNC9_HUMAN (+1) | | 33 kDa | 26 | 6 |
| RL31_HUMAN | RPL31 | 14 kDa | 5 | 25 |
| TOM22_HUMAN | TOMM22 | 16 kDa | 33 | 3 |
| LAP2B_HUMAN | TMPO | 51 kDa | 5 | 5 |
| RBX1_HUMAN | RBX1 | 12 kDa | 0 | 18 |
| UB2V2_HUMAN | UBE2V2 | 16 kDa | 28 | 4 |
| COX5A_HUMAN | COX5A | 17 kDa | 0 | 1 |
| sp\|P00441\|SODC_HUMAN Superoxide dismutase [Cu-Zn] OS=Homo sapiens GN=SOD1 PE=1 SV=2 (+1) | | 16 kDa | 28 | 1 |
| A0A0B4J2C3_HUMAN (+2) | TPT1 | 23 kDa | 29 | 1 |
| BAF_HUMAN | BANF1 | 10 kDa | 0 | 0 |
| SKP1_HUMAN | SKP1 | 19 kDa | 30 | 0 |
| BOLA2_HUMAN | BOLA2 | 10 kDa | 0 | 0 |
| MTND_HUMAN | ADI1 | 21 kDa | 21 | 6 |
| PSB1_HUMAN | PSMB1 | 26 kDa | 32 | 1 |
| BAX_HUMAN | BAX | 21 kDa | 32 | 0 |
| APT_HUMAN | APRT | 20 kDa | 33 | 0 |
| TBB5_HUMAN | TUBB | 50 kDa | 12 | 8 |
| TM14C_HUMAN | TMEM14C | 12 kDa | 1 | 5 |
| MIC13_HUMAN | MIC13 | 13 kDa | 1 | 3 |
| RS23_HUMAN | RPS23 | 16 kDa | 14 | 12 |
| EIF1_HUMAN (+1) | EIF1 | 13 kDa | 0 | 12 |
| ABRAL_HUMAN | ABRACL | 9 kDa | 0 | 0 |
| SF3B5_HUMAN | SF3B5 | 10 kDa | 0 | 0 |
| ATPO_HUMAN | ATP5O | 23 kDa | 28 | 0 |
| TIM13_HUMAN | TIMM13 | 11 kDa | 0 | 0 |
| RAB14_HUMAN | RAB14 | 24 kDa | 29 | 0 |
| TMEDA_HUMAN | TMED10 | 25 kDa | 31 | 0 |
| B8ZZQ6_HUMAN (+1) | PTMA | 12 kDa | 16 | 8 |
| TEBP_HUMAN | PTGES3 | 19 kDa | 22 | 5 |
| SAR1A_HUMAN | SAR1A | 22 kDa | 25 | 0 |
| SRP14_HUMAN | SRP14 | 15 kDa | 20 | 5 |
| PHP14_HUMAN | PHPT1 | 14 kDa | 0 | 17 |
| RL22_HUMAN | RPL22 | 15 kDa | 2 | 14 |
| NDUC2_HUMAN | NDUFC2 | 14 kDa | 0 | 3 |
| RBM8A_HUMAN | RBM8A | 20 kDa | 28 | 1 |
| PSB6_HUMAN | PSMB6 | 25 kDa | 26 | 0 |
| RAB2A_HUMAN | RAB2A | 24 kDa | 28 | 0 |
| DUT_HUMAN (+2) | DUT | 27 kDa | 18 | 6 |
| ELOC_HUMAN | ELOC | 12 kDa | 0 | 0 |
| SH3L1_HUMAN | SH3BGRL | 13 kDa | 0 | 15 |
| PSB4_HUMAN | PSMB4 | 29 kDa | 28 | 0 |
| RAB1A_HUMAN | RAB1A | 23 kDa | 32 | 0 |
| ACTB_HUMAN (+1) | ACTB | 42 kDa | 3 | 5 |
| EF1A1_HUMAN | EEF1A1 | 50 kDa | 8 | 6 |
| H2A2A_HUMAN (+1) | HIST2H2AA3 | 14 kDa | 5 | 14 |
| BTF3_HUMAN | BTF3 | 22 kDa | 23 | 2 |
| LDHB_HUMAN | LDHB | 37 kDa | 7 | 8 |
| COX5B_HUMAN | COX5B | 14 kDa | 0 | 6 |
| LSM2_HUMAN | LSM2 | 11 kDa | 0 | 0 |
| PHS_HUMAN | PCBD1 | 12 kDa | 0 | 0 |
| ATP5H_HUMAN | ATP5H | 18 kDa | 22 | 0 |
| DYR_HUMAN | DHFR | 21 kDa | 25 | 0 |
| NDUA8_HUMAN | NDUFA8 | 20 kDa | 18 | 0 |
| RS26_HUMAN | RPS26 | 13 kDa | 5 | 15 |
| H2B1C_HUMAN (+8) | HIST1H2BC | 14 kDa | 11 | 5 |
| RL28_HUMAN | RPL28 | 16 kDa | 15 | 2 |
| RL35_HUMAN | RPL35 | 15 kDa | 10 | 7 |
| RABP2_HUMAN | CRABP2 | 16 kDa | 0 | 16 |
| EIF3K_HUMAN | EIF3K | 25 kDa | 19 | 0 |
| B0QYN7_HUMAN (+3) | UBE2I | 20 kDa | 21 | 0 |
| E7EQ69_HUMAN (+1) | NAA50 | 19 kDa | 25 | 0 |
| F5GWH5_HUMAN (+1) | TMEM258 | 6 kDa | 0 | 0 |
| F8VZX2_HUMAN | PCBP2 | 34 kDa | 4 | 6 |
| TIM14_HUMAN | DNAJC19 | 12 kDa | 1 | 5 |
| RS25_HUMAN | RPS25 | 14 kDa | 13 | 5 |
| LTOR1_HUMAN | LAMTOR1 | 18 kDa | 19 | 2 |
| NDUA4_HUMAN | NDUFA4 | 9 kDa | 0 | 0 |
| SLIRP_HUMAN | SLIRP | 12 kDa | 0 | 4 |
| PFD5_HUMAN | PFDN5 | 17 kDa | 14 | 4 |
| NTPCR_HUMAN | NTPCR | 21 kDa | 25 | 0 |
| TMED2_HUMAN | TMED2 | 23 kDa | 21 | 0 |
| EDF1_HUMAN | EDF1 | 16 kDa | 13 | 2 |
| NDUF3_HUMAN | NDUFAF3 | 20 kDa | 4 | 16 |
| FIS1_HUMAN | FIS1 | 17 kDa | 7 | 13 |
| CNPY2_HUMAN | CNPY2 | 21 kDa | 21 | 2 |
| ARF4_HUMAN | ARF4 | 21 kDa | 60 | 4 |
| ARL1_HUMAN | ARL1 | 20 kDa | 23 | 0 |
| AT5F1_HUMAN | ATP5F1 | 29 kDa | 20 | 0 |
| MCTS1_HUMAN | MCTS1 | 21 kDa | 22 | 0 |
| NDUAD_HUMAN | NDUFA13 | 17 kDa | 0 | 23 |
| RT25_HUMAN | MRPS25 | 20 kDa | 21 | 0 |
| QCR8_HUMAN | UQCRQ | 10 kDa | 0 | 3 |
| A0A087WUM0_HUMAN | SYNJ2BP-COX16 | 21 kDa | 0 | 7 |
| S35U4_HUMAN | SLC35A4 | 11 kDa | 0 | 0 |
| SMD1_HUMAN | SNRPD1 | 13 kDa | 5 | 14 |
| LSM4_HUMAN (+1) | LSM4 | 15 kDa | 3 | 17 |
| NDKA_HUMAN | NME1 | 17 kDa | 76 | 114 |
| ARL8A_HUMAN | ARL8A | 21 kDa | 22 | 0 |
| DNPH1_HUMAN (+1) | DNPH1 | 19 kDa | 18 | 0 |
| MD2L1_HUMAN | MAD2L1 | 24 kDa | 20 | 0 |
| ATP8_HUMAN | MT-ATP8 | 8 kDa | 0 | 0 |
| J3QRU4_HUMAN (+2) | VAMP2 | 12 kDa | 2 | 9 |
| DAD1_HUMAN (+1) | DAD1 | 12 kDa | 1 | 1 |
| ATPD_HUMAN | ATP5D | 17 kDa | 5 | 10 |
| SAP_HUMAN | PSAP | 58 kDa | 0 | 2 |
| RFA1_HUMAN | RPA1 | 68 kDa | 14 | 3 |
| A6NLH6_HUMAN (+1) | CNIH4 | 16 kDa | 1 | 2 |
| C9JNW5_HUMAN (+2) | RPL24 | 18 kDa | 15 | 0 |
| CISD2_HUMAN | CISD2 | 15 kDa | 0 | 12 |
| PFD1_HUMAN | PFDN1 | 14 kDa | 0 | 11 |
| RHOG_HUMAN | RHOG | 21 kDa | 12 | 7 |
| SC61B_HUMAN | SEC61B | 10 kDa | 0 | 0 |
| TPPC3_HUMAN | TRAPPC3 | 20 kDa | 14 | 4 |
| C9JLU1_HUMAN (+1) | POLR2H | 17 kDa | 17 | 3 |
| RT21_HUMAN | MRPS21 | 11 kDa | 0 | 3 |
| RAP1B_HUMAN | RAP1B | 21 kDa | 20 | 0 |
| NDUAC_HUMAN | NDUFA12 | 17 kDa | 19 | 1 |
| DCTP1_HUMAN | DCTPP1 | 19 kDa | 17 | 1 |
| NDUS7_HUMAN | NDUFS7 | 24 kDa | 17 | 0 |
| C9J0K6_HUMAN (+1) | SRI | 18 kDa | 18 | 0 |
| J3KNF8_HUMAN | CYB5B | 17 kDa | 17 | 0 |
| PPIH_HUMAN | PPIH | 19 kDa | 19 | 0 |
| RET1_HUMAN | RBP1 | 16 kDa | 0 | 18 |
| TXN4A_HUMAN | TXNL4A | 17 kDa | 0 | 19 |
| C9JXK0_HUMAN (+1) | LBR | 24 kDa | 6 | 4 |
| ATPK_HUMAN | ATP5J2 | 11 kDa | 1 | 4 |
| J3KQN4_HUMAN (+1) | RPL36A | 16 kDa | 11 | 5 |
| AP2S1_HUMAN (+2) | AP2S1 | 17 kDa | 0 | 9 |
| MYDGF_HUMAN | MYDGF | 19 kDa | 7 | 10 |
| NDK3_HUMAN | NME3 | 19 kDa | 13 | 7 |
| SC11A_HUMAN | SEC11A | 21 kDa | 7 | 14 |
| E9PL57_HUMAN | NEDD8-MDP1 | 20 kDa | 5 | 0 |
| RL27A_HUMAN | RPL27A | 17 kDa | 15 | 3 |
| ACO13_HUMAN | ACOT13 | 15 kDa | 1 | 15 |
| ARPC3_HUMAN | ARPC3 | 21 kDa | 18 | 0 |
| ARPC4_HUMAN (+1) | ARPC4 | 20 kDa | 16 | 0 |
| ATP5I_HUMAN | ATP5I | 8 kDa | 0 | 0 |
| BT3L4_HUMAN | BTF3L4 | 17 kDa | 21 | 0 |
| SF3B6_HUMAN | SF3B6 | 15 kDa | 0 | 17 |
| SNX3_HUMAN | SNX3 | 19 kDa | 21 | 0 |
| PDLI1_HUMAN | PDLIM1 | 36 kDa | 5 | 7 |
| A0A087X0X3_HUMAN (+1) | HNRNPM | 78 kDa | 3 | 13 |
| RLA1_HUMAN | RPLP1 | 12 kDa | 3 | 13 |
| H0YAT2_HUMAN (+1) | MRPS28 | 16 kDa | 12 | 4 |
| TXD17_HUMAN | TXNDC17 | 14 kDa | 0 | 4 |
| RM17_HUMAN | MRPL17 | 20 kDa | 13 | 2 |
| QCR7_HUMAN | UQCRB | 14 kDa | 0 | 2 |
| DEST_HUMAN | DSTN | 19 kDa | 23 | 1 |
| DBLOH_HUMAN | DIABLO | 27 kDa | 20 | 0 |
| PFD2_HUMAN | PFDN2 | 17 kDa | 20 | 0 |
| RAB1B_HUMAN | RAB1B | 22 kDa | 34 | 0 |
| B4DLN1_HUMAN (+1) | | 48 kDa | 10 | 0 |
| NDUB4_HUMAN | NDUFB4 | 15 kDa | 0 | 9 |
| TIM10_HUMAN | TIMM10 | 10 kDa | 0 | 0 |
| MTPN_HUMAN | MTPN | 13 kDa | 0 | 1 |
| Q5VST9\|OBSCN_HUMAN-DECOY | | ? | 0 | 0 |
| CSN8_HUMAN | COPS8 | 23 kDa | 17 | 0 |
| GGCT_HUMAN | GGCT | 21 kDa | 18 | 0 |
| KAD1_HUMAN (+1) | AK1 | 22 kDa | 19 | 0 |
| PDCD6_HUMAN | PDCD6 | 22 kDa | 13 | 0 |
| PEA15_HUMAN | PEA15 | 15 kDa | 0 | 15 |
| RAC1_HUMAN | RAC1 | 21 kDa | 16 | 0 |
| RL21_HUMAN | RPL21 | 19 kDa | 14 | 0 |
| YKT6_HUMAN | YKT6 | 22 kDa | 17 | 0 |
| ENOA_HUMAN | ENO1 | 47 kDa | 5 | 4 |
| E9PK01_HUMAN (+1) | EEF1D | 29 kDa | 3 | 10 |
| CATC_HUMAN | CTSC | 52 kDa | 10 | 1 |
| AT5G1_HUMAN (+5) | ATP5G1 | 14 kDa | 1 | 5 |
| CGBP1_HUMAN | CGGBP1 | 19 kDa | 12 | 0 |
| NDUF4_HUMAN | NDUFAF4 | 20 kDa | 14 | 1 |
| AIF1L_HUMAN | AIF1L | 17 kDa | 7 | 5 |
| AP1S2_HUMAN | AP1S2 | 19 kDa | 9 | 5 |
| NDUA2_HUMAN | NDUFA2 | 11 kDa | 0 | 0 |
| RBM3_HUMAN | RBM3 | 17 kDa | 11 | 6 |
| RIDA_HUMAN | RIDA | 14 kDa | 0 | 7 |
| RT06_HUMAN | MRPS6 | 14 kDa | 0 | 8 |
| SSRD_HUMAN | SSR4 | 19 kDa | 8 | 7 |
| GBRL2_HUMAN | GABARAPL2 | 14 kDa | 0 | 8 |
| RL34_HUMAN | RPL34 | 13 kDa | 3 | 13 |
| ARPC5_HUMAN | ARPC5 | 16 kDa | 6 | 6 |
| VATL_HUMAN | ATP6V0C | 16 kDa | 0 | 4 |
| GMFB_HUMAN | GMFB | 17 kDa | 12 | 2 |
| NDUB6_HUMAN | NDUFB6 | 15 kDa | 3 | 9 |
| C560_HUMAN | SDHC | 19 kDa | 0 | 0 |
| IFT25_HUMAN | HSPB11 | 16 kDa | 16 | 0 |
| NDUA7_HUMAN | NDUFA7 | 13 kDa | 0 | 16 |
| PMVK_HUMAN | PMVK | 22 kDa | 15 | 0 |
| PPAC_HUMAN | ACP1 | 18 kDa | 18 | 0 |
| PSMG4_HUMAN | PSMG4 | 14 kDa | 0 | 0 |
| RM47_HUMAN | MRPL47 | 29 kDa | 15 | 0 |
| VPS29_HUMAN | VPS29 | 21 kDa | 16 | 0 |
| X6RAL5_HUMAN | SAP18 | 20 kDa | 16 | 0 |
| A0A0G2JIW1_HUMAN (+2) | HSPA1B | 70 kDa | 6 | 2 |
| H0YIV9_HUMAN | | 19 kDa | 0 | 2 |
| sp\|BID_HUMAN\| (+1) | | 22 kDa | 14 | 0 |
| CCD58_HUMAN | CCDC58 | 17 kDa | 4 | 9 |
| OCAD2_HUMAN | OCIAD2 | 17 kDa | 7 | 6 |
| PSMG3_HUMAN | PSMG3 | 13 kDa | 0 | 4 |
| ARF5_HUMAN | ARF5 | 21 kDa | 56 | 5 |
| NDUA3_HUMAN (+1) | NDUFA3 | 9 kDa | 0 | 0 |
| ARL3_HUMAN | ARL3 | 20 kDa | 15 | 0 |
| FKBP2_HUMAN | FKBP2 | 16 kDa | 0 | 13 |
| NDUS8_HUMAN | NDUFS8 | 24 kDa | 13 | 0 |
| RM11_HUMAN | MRPL11 | 21 kDa | 15 | 0 |
| RM13_HUMAN | MRPL13 | 21 kDa | 17 | 0 |
| RM50_HUMAN | MRPL50 | 18 kDa | 0 | 13 |
| PAIRB_HUMAN | SERBP1 | 45 kDa | 4 | 2 |
| MARE1_HUMAN | MAPRE1 | 30 kDa | 8 | 2 |
| K7ENI6_HUMAN (+2) | TMEM256-PLSCR3 | 4 kDa | 1 | 4 |
| ERH_HUMAN | ERH | 12 kDa | 0 | 1 |
| E7EPV7_HUMAN (+2) | SNCA | 12 kDa | 1 | 13 |
| A0A075B6Z2_HUMAN | TRAJ56 | 2 kDa | 0 | 1 |
| LEG1_HUMAN | LGALS1 | 15 kDa | 0 | 6 |
| PHF5A_HUMAN | PHF5A | 12 kDa | 0 | 4 |
| S10AB_HUMAN | S100A11 | 12 kDa | 0 | 0 |
| PTRD1_HUMAN | PTRHD1 | 16 kDa | 0 | 3 |
| TR112_HUMAN | TRMT112 | 14 kDa | 0 | 2 |
| AP3S1_HUMAN | AP3S1 | 22 kDa | 9 | 3 |
| VMA21_HUMAN | VMA21 | 11 kDa | 0 | 0 |
| F162A_HUMAN (+1) | FAM162A | 17 kDa | 0 | 10 |
| F136A_HUMAN | FAM136A | 16 kDa | 2 | 12 |
| TXD12_HUMAN | TXNDC12 | 19 kDa | 13 | 1 |
| A8K878_HUMAN (+1) | MANF | 21 kDa | 13 | 0 |
| CISD1_HUMAN | CISD1 | 12 kDa | 0 | 0 |
| FAIM1_HUMAN | FAIM | 20 kDa | 16 | 0 |
| GNA1_HUMAN | GNPNAT1 | 21 kDa | 16 | 0 |
| LZIC_HUMAN | LZIC | 21 kDa | 15 | 0 |
| NUD16_HUMAN | NUDT16 | 21 kDa | 16 | 0 |
| PFD6_HUMAN | PFDN6 | 15 kDa | 0 | 11 |
| RHEB_HUMAN | RHEB | 20 kDa | 12 | 0 |
| USMG5_HUMAN | USMG5 | 6 kDa | 0 | 0 |
| URM1_HUMAN | URM1 | 11 kDa | 0 | 2 |
| MGST3_HUMAN (+1) | MGST3 | 17 kDa | 2 | 11 |
| AAMDC_HUMAN | AAMDC | 13 kDa | 0 | 5 |
| DPOE3_HUMAN | POLE3 | 17 kDa | 7 | 5 |
| FUND2_HUMAN | FUNDC2 | 21 kDa | 6 | 8 |
| GLRX5_HUMAN | GLRX5 | 17 kDa | 0 | 4 |
| HINT2_HUMAN | HINT2 | 17 kDa | 3 | 9 |
| MMGT1_HUMAN | MMGT1 | 15 kDa | 0 | 3 |
| NCBP2_HUMAN | NCBP2 | 18 kDa | 4 | 5 |
| NENF_HUMAN | NENF | 19 kDa | 7 | 8 |
| NH2L1_HUMAN | SNU13 | 14 kDa | 0 | 5 |
| RM23_HUMAN | MRPL23 | 18 kDa | 11 | 3 |
| DLRB1_HUMAN | DYNLRB1 | 11 kDa | 0 | 0 |
| RT14_HUMAN | MRPS14 | 15 kDa | 0 | 4 |
| CHSP1_HUMAN | CARHSP1 | 16 kDa | 10 | 4 |
| MPC2_HUMAN (+1) | MPC2 | 14 kDa | 0 | 9 |
| COPZ1_HUMAN (+1) | COPZ1 | 20 kDa | 13 | 1 |
| MCA3_HUMAN | EEF1E1 | 20 kDa | 14 | 1 |
| ARHGH_HUMAN | ARHGEF17 | 222 kDa | 0 | 0 |
| COMD2_HUMAN | COMMD2 | 23 kDa | 13 | 0 |
| CT027_HUMAN | C20orf27 | 19 kDa | 13 | 0 |
| E7ESL0_HUMAN (+2) | MRPL22 | 24 kDa | 15 | 0 |
| HYPK_HUMAN | HYPK | 15 kDa | 0 | 13 |
| ISOC2_HUMAN | ISOC2 | 22 kDa | 13 | 0 |
| KCY_HUMAN | CMPK1 | 22 kDa | 15 | 0 |
| NUD11_HUMAN | NUDT11 | 19 kDa | 12 | 0 |
| RT10_HUMAN | MRPS10 | 23 kDa | 12 | 0 |
| SPRY4_HUMAN | SPRYD4 | 23 kDa | 14 | 0 |
| UB2L3_HUMAN | UBE2L3 | 18 kDa | 15 | 0 |
| UBC12_HUMAN | UBE2M | 21 kDa | 13 | 0 |
| H33_HUMAN (+1) | H3F3A | 15 kDa | 5 | 3 |
| G3P_HUMAN | GAPDH | 36 kDa | 7 | 3 |
| EMD_HUMAN | EMD | 29 kDa | 2 | 8 |
| H7C1U8_HUMAN (+1) | APOO | 20 kDa | 10 | 3 |
| A6NMQ3_HUMAN (+2) | ENSA | 16 kDa | 7 | 6 |
| AP4A_HUMAN | NUDT2 | 17 kDa | 5 | 5 |
| NDUAB_HUMAN | NDUFA11 | 15 kDa | 0 | 6 |
| Q9Y3D0\|CIA2B_HUMAN | | ? | 6 | 8 |
| SGMR2_HUMAN | TMEM97 | 21 kDa | 4 | 8 |
| BUD31_HUMAN | BUD31 | 17 kDa | 9 | 3 |
| LTOR4_HUMAN | LAMTOR4 | 11 kDa | 0 | 0 |
| OARD1_HUMAN | OARD1 | 17 kDa | 8 | 3 |
| VAPA_HUMAN | VAPA | 28 kDa | 3 | 0 |
| ENY2_HUMAN | ENY2 | 12 kDa | 0 | 0 |
| RM20_HUMAN | MRPL20 | 17 kDa | 1 | 7 |
| 68MP_HUMAN (+2) | MP68 | 7 kDa | 0 | 1 |
| HIKES_HUMAN | HIKESHI | 22 kDa | 11 | 0 |
| SH3L3_HUMAN | SH3BGRL3 | 10 kDa | 0 | 0 |
| LTOR2_HUMAN | LAMTOR2 | 14 kDa | 0 | 0 |
| A0A087X0R6_HUMAN (+1) | SNX12 | 20 kDa | 13 | 0 |
| COA3_HUMAN | COA3 | 12 kDa | 0 | 0 |
| COTL1_HUMAN | COTL1 | 16 kDa | 0 | 12 |
| J3KQ48_HUMAN (+1) | PTRH2 | 19 kDa | 11 | 0 |
| LSM7_HUMAN | LSM7 | 12 kDa | 0 | 0 |
| NAA20_HUMAN | NAA20 | 20 kDa | 13 | 0 |
| NDUS6_HUMAN | NDUFS6 | 14 kDa | 0 | 0 |
| RL6_HUMAN | RPL6 | 33 kDa | 11 | 0 |
| RPB7_HUMAN | POLR2G | 19 kDa | 13 | 0 |
| VPS25_HUMAN | VPS25 | 21 kDa | 13 | 0 |
| HNRPK_HUMAN | HNRNPK | 51 kDa | 5 | 1 |
| 2AAA_HUMAN | PPP2R1A | 65 kDa | 4 | 0 |
| RS10_HUMAN | RPS10 | 19 kDa | 31 | 7 |
| C10_HUMAN | C12orf57 | 13 kDa | 0 | 4 |
| ISCU_HUMAN | ISCU | 18 kDa | 6 | 3 |
| UBE2A_HUMAN | UBE2A | 17 kDa | 4 | 7 |
| RT24_HUMAN | MRPS24 | 19 kDa | 2 | 7 |
| E7EWP0_HUMAN (+2) | NDUFB5 | 20 kDa | 4 | 6 |
| NDUA5_HUMAN | NDUFA5 | 13 kDa | 0 | 2 |
| TPPC1_HUMAN | TRAPPC1 | 17 kDa | 1 | 5 |
| NOP10_HUMAN | NOP10 | 8 kDa | 0 | 0 |
| TPC6B_HUMAN | TRAPPC6B | 18 kDa | 2 | 10 |
| DYL2_HUMAN | DYNLL2 | 10 kDa | 0 | 0 |
| OSTC_HUMAN | OSTC | 17 kDa | 0 | 2 |
| RTL8C_HUMAN | RTL8C | 13 kDa | 0 | 0 |
| NDUB7_HUMAN | NDUFB7 | 16 kDa | 11 | 1 |
| E9PQY2_HUMAN (+1) | PFDN4 | 16 kDa | 1 | 12 |
| DCTD_HUMAN | DCTD | 20 kDa | 10 | 1 |
| CNBP_HUMAN | CNBP | 19 kDa | 10 | 1 |
| C9JYQ9_HUMAN (+2) | RPL22L1 | 14 kDa | 1 | 10 |
| 8ODP_HUMAN | NUDT1 | 23 kDa | 10 | 0 |
| ACPM_HUMAN | NDUFAB1 | 17 kDa | 0 | 0 |
| ARF6_HUMAN | ARF6 | 20 kDa | 11 | 0 |
| CAP1_HUMAN | CAP1 | 52 kDa | 11 | 0 |
| COF2_HUMAN | CFL2 | 19 kDa | 30 | 0 |
| CRADD_HUMAN | CRADD | 23 kDa | 11 | 0 |
| NDKM_HUMAN (+1) | NME4 | 21 kDa | 10 | 0 |
| NDUB3_HUMAN | NDUFB3 | 11 kDa | 0 | 0 |
| NUDC2_HUMAN | NUDCD2 | 18 kDa | 12 | 0 |
| PP14B_HUMAN | PPP1R14B | 16 kDa | 13 | 0 |
| PPIL1_HUMAN | PPIL1 | 18 kDa | 11 | 0 |
| Q5QPA5_HUMAN (+1) | MRPS18A | 30 kDa | 11 | 0 |
| SODM_HUMAN | SOD2 | 25 kDa | 12 | 0 |
| TI17B_HUMAN | TIMM17B | 18 kDa | 11 | 0 |
| TI23B_HUMAN (+1) | TIMM23B | 28 kDa | 13 | 0 |
| TP4A2_HUMAN | PTP4A2 | 19 kDa | 12 | 0 |
| UB2G1_HUMAN | UBE2G1 | 20 kDa | 10 | 0 |
| UFC1_HUMAN | UFC1 | 19 kDa | 10 | 0 |
| F8W6I7_HUMAN (+1) | HNRNPA1 | 33 kDa | 3 | 3 |
| NPM_HUMAN | NPM1 | 33 kDa | 3 | 2 |
| CDKA1_HUMAN | CDK2AP1 | 12 kDa | 1 | 1 |
| PCNP_HUMAN | PCNP | 19 kDa | 8 | 1 |
| ATPB_HUMAN | ATP5B | 57 kDa | 2 | 0 |
| A0A087WYS0_HUMAN (+3) | IMMT | 41 kDa | 0 | 0 |
| DPOE4_HUMAN | POLE4 | 12 kDa | 5 | 7 |
| JAGN1_HUMAN | JAGN1 | 21 kDa | 4 | 7 |
| LYRM7_HUMAN | LYRM7 | 12 kDa | 0 | 3 |
| RM49_HUMAN | MRPL49 | 19 kDa | 9 | 3 |
| TM160_HUMAN | TMEM160 | 20 kDa | 3 | 8 |
| C2AIL_HUMAN | CDKN2AIPNL | 13 kDa | 0 | 0 |
| RT33_HUMAN | MRPS33 | 13 kDa | 0 | 4 |
| GLRX1_HUMAN | GLRX | 12 kDa | 0 | 0 |
| J3QQ67_HUMAN (+1) | RPL18 | 22 kDa | 8 | 2 |
| H7C5U8_HUMAN (+1) | MRPL27 | 15 kDa | 1 | 6 |
| CKS1_HUMAN | CKS1B | 10 kDa | 0 | 0 |
| COX17_HUMAN | COX17 | 7 kDa | 0 | 0 |
| BOLA1_HUMAN | BOLA1 | 14 kDa | 0 | 1 |
| UBL4A_HUMAN | UBL4A | 18 kDa | 7 | 0 |
| RM55_HUMAN | MRPL55 | 15 kDa | 0 | 0 |
| ARL6_HUMAN | ARL6 | 21 kDa | 10 | 0 |
| CDC42_HUMAN | CDC42 | 21 kDa | 11 | 0 |
| CDIPT_HUMAN | CDIPT | 24 kDa | 11 | 0 |
| COMD1_HUMAN | COMMD1 | 21 kDa | 10 | 0 |
| COMD3_HUMAN | COMMD3 | 22 kDa | 11 | 0 |
| COMD4_HUMAN | COMMD4 | 22 kDa | 11 | 0 |
| CUTA_HUMAN | CUTA | 19 kDa | 0 | 9 |
| DCTN3_HUMAN | DCTN3 | 21 kDa | 12 | 0 |
| IFT27_HUMAN | IFT27 | 20 kDa | 9 | 0 |
| LTOR5_HUMAN | LAMTOR5 | 10 kDa | 0 | 0 |
| NAT14_HUMAN | NAT14 | 22 kDa | 9 | 0 |
| NDUB9_HUMAN | NDUFB9 | 22 kDa | 10 | 0 |
| NDUF2_HUMAN | NDUFAF2 | 20 kDa | 10 | 0 |
| PGRC2_HUMAN | PGRMC2 | 24 kDa | 10 | 0 |
| PLRKT_HUMAN | PLGRKT | 17 kDa | 0 | 9 |
| PPIF_HUMAN | PPIF | 22 kDa | 11 | 0 |
| RAB10_HUMAN | RAB10 | 23 kDa | 18 | 0 |
| RM18_HUMAN | MRPL18 | 21 kDa | 12 | 0 |
| RM21_HUMAN | MRPL21 | 23 kDa | 11 | 0 |
| SAR1B_HUMAN | SAR1B | 22 kDa | 20 | 0 |
| SC22B_HUMAN | SEC22B | 25 kDa | 11 | 0 |
| VAMP7_HUMAN | VAMP7 | 25 kDa | 10 | 0 |
| sp\|UBE2C_HUMAN\| (+1) | | 20 kDa | 12 | 0 |
| TBB4B_HUMAN | TUBB4B | 50 kDa | 11 | 8 |
| CH60_HUMAN | HSPD1 | 61 kDa | 3 | 1 |
| EIF3G_HUMAN | EIF3G | 36 kDa | 2 | 0 |
| A0A0C4DGB6_HUMAN (+3) | ALB | 69 kDa | 0 | 3 |
| VAMP3_HUMAN | VAMP3 | 11 kDa | 0 | 4 |
| I3L0E3_HUMAN | hCG_1984214 | 26 kDa | 3 | 3 |
| ACYP1_HUMAN | ACYP1 | 11 kDa | 0 | 0 |
| C9JAX1_HUMAN (+1) | FXN | 15 kDa | 0 | 5 |
| SNAPN_HUMAN | SNAPIN | 15 kDa | 5 | 3 |
| TM109_HUMAN | TMEM109 | 26 kDa | 4 | 5 |
| A0A0U1RQV1_HUMAN (+1) | | 12 kDa | 0 | 6 |
| UB2D3_HUMAN | UBE2D3 | 17 kDa | 3 | 7 |
| ASPG_HUMAN | AGA | 37 kDa | 7 | 3 |
| BRI3B_HUMAN | BRI3BP | 28 kDa | 6 | 4 |
| E7EN96_HUMAN (+4) | MED22 | 16 kDa | 2 | 8 |
| RPAC2_HUMAN | POLR1D | 15 kDa | 0 | 9 |
| NDUS5_HUMAN | NDUFS5 | 13 kDa | 0 | 2 |
| MED31_HUMAN | MED31 | 16 kDa | 2 | 7 |
| LAGE3_HUMAN | LAGE3 | 15 kDa | 8 | 0 |
| SUMO2_HUMAN | SUMO2 | 11 kDa | 1 | 7 |
| RPP14_HUMAN | RPP14 | 14 kDa | 0 | 1 |
| MOC2B_HUMAN | MOCS2 | 21 kDa | 10 | 1 |
| RM14_HUMAN | MRPL14 | 16 kDa | 0 | 9 |
| ORML3_HUMAN | ORMDL3 | 17 kDa | 0 | 9 |
| A0A1B0GTM3_HUMAN (+14) | ASAH1 | 42 kDa | 0 | 0 |
| CFA20_HUMAN | CFAP20 | 23 kDa | 9 | 0 |
| CRGD_HUMAN | CRYGD | 21 kDa | 10 | 0 |
| ERD21_HUMAN | KDELR1 | 25 kDa | 14 | 0 |
| ERD22_HUMAN | KDELR2 | 24 kDa | 10 | 0 |
| EXOS1_HUMAN | EXOSC1 | 21 kDa | 10 | 0 |
| F5H702_HUMAN (+1) | MRPL48 | 13 kDa | 10 | 0 |
| HPCL1_HUMAN | HPCAL1 | 22 kDa | 10 | 0 |
| KGUA_HUMAN | GUK1 | 22 kDa | 11 | 0 |
| PSF1_HUMAN | GINS1 | 23 kDa | 9 | 0 |
| PTPM1_HUMAN | PTPMT1 | 23 kDa | 9 | 0 |
| RAB31_HUMAN | RAB31 | 22 kDa | 10 | 0 |
| RAB4A_HUMAN | RAB4A | 24 kDa | 13 | 0 |
| TPRKB_HUMAN | TPRKB | 20 kDa | 11 | 0 |
| A0A1W2PNR9_HUMAN (+1) | IER3IP1 | 8 kDa | 0 | 0 |
| KPYM_HUMAN (+1) | PKM | 58 kDa | 1 | 0 |
| A0A087WXM6_HUMAN (+3) | RPL17 | 20 kDa | 3 | 2 |
| A0A0J9YVP6_HUMAN (+4) | PUF60 | 57 kDa | 3 | 4 |
| THOC4_HUMAN | ALYREF | 27 kDa | 4 | 0 |
| RU2B_HUMAN | SNRPB2 | 25 kDa | 7 | 2 |
| HIG1A_HUMAN | HIGD1A | 10 kDa | 0 | 1 |
| ATPG_HUMAN | ATP5C1 | 33 kDa | 6 | 0 |
| 12345 |  | 24 kDa | 10 | 24 |
| BL1S1_HUMAN | BLOC1S1 | 17 kDa | 0 | 4 |
| F8W914_HUMAN | RTN4 | 37 kDa | 5 | 5 |
| RM54_HUMAN | MRPL54 | 16 kDa | 0 | 3 |
| RM41_HUMAN | MRPL41 | 15 kDa | 0 | 6 |
| sp\|CYB5_HUMAN\| (+1) | | 15 kDa | 2 | 7 |
| F241A_HUMAN | FAM241A | 15 kDa | 3 | 4 |
| GOGA7_HUMAN | GOLGA7 | 16 kDa | 0 | 7 |
| ISCA2_HUMAN | ISCA2 | 16 kDa | 0 | 4 |
| A0A0B4J294_HUMAN (+2) | TRAPPC2L | 17 kDa | 0 | 6 |
| B8ZZ87_HUMAN (+1) | MZT2B | 22 kDa | 8 | 2 |
| LTOR3_HUMAN | LAMTOR3 | 14 kDa | 0 | 1 |
| I3L0A0_HUMAN (+1) | TMEM189-UBE2V1 | 42 kDa | 27 | 6 |
| A0A0A6YYL4_HUMAN (+3) | CORO7-PAM16 | 114 kDa | 0 | 6 |
| CANB1_HUMAN (+2) | PPP3R1 | 19 kDa | 7 | 1 |
| DYLT1_HUMAN | DYNLT1 | 12 kDa | 0 | 1 |
| NR2CA_HUMAN | NR2C2AP | 16 kDa | 1 | 7 |
| LYRM2_HUMAN | LYRM2 | 10 kDa | 0 | 0 |
| LIN7C_HUMAN | LIN7C | 22 kDa | 8 | 0 |
| NDUA1_HUMAN | NDUFA1 | 8 kDa | 0 | 0 |
| REEP5_HUMAN | REEP5 | 21 kDa | 8 | 0 |
| B1AJY5_HUMAN (+2) | PSMD10 | 20 kDa | 8 | 0 |
| BET1_HUMAN | BET1 | 13 kDa | 0 | 0 |
| SIM12_HUMAN | SMIM12 | 11 kDa | 0 | 0 |
| LSM1_HUMAN | LSM1 | 15 kDa | 0 | 9 |
| TM263_HUMAN | TMEM263 | 12 kDa | 0 | 6 |
| A0A087WT12_HUMAN (+2) | GPX4 | 27 kDa | 9 | 0 |
| A0A087WUQ6_HUMAN (+1) | GPX1 | 22 kDa | 10 | 0 |
| A0A0C4DGS0_HUMAN (+2) | NDUFA6 | 15 kDa | 0 | 9 |
| AF1Q_HUMAN | MLLT11 | 10 kDa | 0 | 0 |
| APC16_HUMAN | ANAPC16 | 12 kDa | 0 | 0 |
| B1AL05_HUMAN (+1) | MRPL43 | 21 kDa | 5 | 0 |
| COQ8A_HUMAN | COQ8A | 72 kDa | 0 | 10 |
| DCAKD_HUMAN (+1) | DCAKD | 27 kDa | 10 | 0 |
| DUS3_HUMAN | DUSP3 | 20 kDa | 10 | 0 |
| E9PN81_HUMAN (+1) | RNASEH2C | 26 kDa | 9 | 0 |
| ERI3_HUMAN (+1) | ERI3 | 37 kDa | 7 | 0 |
| F210A_HUMAN (+1) | FAM210A | 31 kDa | 9 | 0 |
| F8W8H5_HUMAN (+1) | RAB24 | 20 kDa | 8 | 0 |
| FRIH_HUMAN | FTH1 | 21 kDa | 10 | 0 |
| H3BNT4_HUMAN (+1) | MPHOSPH6 | 17 kDa | 7 | 0 |
| LSM6_HUMAN | LSM6 | 9 kDa | 0 | 0 |
| LSM8_HUMAN | LSM8 | 10 kDa | 0 | 0 |
| MED20_HUMAN | MED20 | 23 kDa | 9 | 0 |
| NDUB8_HUMAN | NDUFB8 | 22 kDa | 9 | 0 |
| RAB8A_HUMAN | RAB8A | 24 kDa | 16 | 0 |
| RT36_HUMAN | MRPS36 | 11 kDa | 0 | 0 |
| SH3L2_HUMAN | SH3BGRL2 | 12 kDa | 0 | 0 |
| SRP19_HUMAN | SRP19 | 16 kDa | 10 | 0 |
| T126A_HUMAN | TMEM126A | 22 kDa | 9 | 0 |
| TIRR_HUMAN | NUDT16L1 | 23 kDa | 10 | 0 |
| ADX_HUMAN | FDX1 | 19 kDa | 0 | 0 |
| GBG5_HUMAN | GNG5 | 7 kDa | 0 | 0 |
| BOLA3_HUMAN | BOLA3 | 12 kDa | 0 | 0 |
| ATPA_HUMAN | ATP5A1 | 60 kDa | 1 | 2 |
| RL8_HUMAN | RPL8 | 28 kDa | 2 | 2 |
| CBX5_HUMAN | CBX5 | 22 kDa | 1 | 4 |
| PCBP1_HUMAN | PCBP1 | 37 kDa | 3 | 1 |
| PRDX6_HUMAN | PRDX6 | 25 kDa | 5 | 1 |
| EF1G_HUMAN | EEF1G | 50 kDa | 4 | 0 |
| J3QLE5_HUMAN (+2) | SNRPN | 18 kDa | 3 | 3 |
| B2R5W2_HUMAN (+3) | HNRNPC | 32 kDa | 5 | 2 |
| MIC10_HUMAN | MINOS1 | 9 kDa | 0 | 1 |
| HIG2A_HUMAN | HIGD2A | 12 kDa | 0 | 2 |
| RS30_HUMAN | FAU | 7 kDa | 1 | 0 |
| COQ7_HUMAN (+1) | COQ7 | 24 kDa | 4 | 4 |
| POP5_HUMAN | POP5 | 19 kDa | 4 | 3 |
| PDE6D_HUMAN | PDE6D | 17 kDa | 6 | 3 |
| FMC1_HUMAN | FMC1 | 13 kDa | 0 | 3 |
| A0A3B3ISV4\|A0A3B3ISV4_HUMAN | | ? | 7 | 2 |
| LSM5_HUMAN | LSM5 | 10 kDa | 0 | 0 |
| LSM3_HUMAN | LSM3 | 12 kDa | 0 | 2 |
| TPIS_HUMAN | TPI1 | 31 kDa | 5 | 0 |
| A6NMH8_HUMAN (+6) | CD81 | 30 kDa | 2 | 3 |
| B4DI03_HUMAN (+2) | SEC11C | 17 kDa | 3 | 4 |
| PRAF3_HUMAN | ARL6IP5 | 22 kDa | 4 | 2 |
| sp\|Q99LS0\|AUGN_MOUSE Augurin OS=Mus musculus OX=10090 GN=Ecrg4 PE=1 SV=2 | | 17 kDa | 7 | 1 |
| SSNA1_HUMAN | SSNA1 | 14 kDa | 0 | 6 |
| A0A0B4J220_HUMAN | C11orf98 | 14 kDa | 6 | 1 |
| AR2BP_HUMAN | ARL2BP | 19 kDa | 6 | 1 |
| ATP5J_HUMAN | ATP5J | 13 kDa | 0 | 0 |
| TIM9_HUMAN | TIMM9 | 10 kDa | 0 | 0 |
| LYAG_HUMAN | GAA | 105 kDa | 7 | 0 |
| GDIR1_HUMAN | ARHGDIA | 23 kDa | 8 | 0 |
| A0A0A0MR06_HUMAN (+3) | RER1 | 21 kDa | 8 | 0 |
| ABC3C_HUMAN | APOBEC3C | 23 kDa | 9 | 0 |
| ARL2_HUMAN | ARL2 | 21 kDa | 8 | 0 |
| ASURF_HUMAN | ASDURF | 11 kDa | 0 | 0 |
| B4E1G1_HUMAN (+1) | DERL1 | 17 kDa | 5 | 0 |
| BRK1_HUMAN | BRK1 | 9 kDa | 0 | 0 |
| CHP1_HUMAN | CHP1 | 22 kDa | 9 | 0 |
| COMD9_HUMAN | COMMD9 | 22 kDa | 9 | 0 |
| COX11_HUMAN | COX11 | 31 kDa | 6 | 0 |
| D6RAN4_HUMAN (+1) | RPL9 | 21 kDa | 8 | 0 |
| D6REQ6_HUMAN (+2) | RNASET2 | 25 kDa | 8 | 0 |
| J9JIE6_HUMAN (+1) | TMCO1 | 27 kDa | 8 | 0 |
| KAD6_HUMAN | AK6 | 20 kDa | 8 | 0 |
| LYRM4_HUMAN | LYRM4 | 11 kDa | 0 | 0 |
| MIA40_HUMAN | CHCHD4 | 16 kDa | 8 | 0 |
| NCS1_HUMAN | NCS1 | 22 kDa | 8 | 0 |
| NDUS4_HUMAN | NDUFS4 | 20 kDa | 8 | 0 |
| NUD15_HUMAN | NUDT15 | 19 kDa | 7 | 0 |
| PEBB_HUMAN | CBFB | 22 kDa | 7 | 0 |
| PPIL3_HUMAN | PPIL3 | 18 kDa | 7 | 0 |
| PXMP2_HUMAN | PXMP2 | 22 kDa | 7 | 0 |
| RAB13_HUMAN | RAB13 | 23 kDa | 13 | 0 |
| RAB18_HUMAN | RAB18 | 23 kDa | 8 | 0 |
| RAB9A_HUMAN | RAB9A | 23 kDa | 9 | 0 |
| RBBP9_HUMAN | RBBP9 | 21 kDa | 7 | 0 |
| RS8_HUMAN | RPS8 | 24 kDa | 9 | 0 |
| RT18C_HUMAN | MRPS18C | 16 kDa | 0 | 9 |
| THEM6_HUMAN | THEM6 | 24 kDa | 9 | 0 |
| TMED5_HUMAN | TMED5 | 26 kDa | 9 | 0 |
| TMM65_HUMAN | TMEM65 | 25 kDa | 9 | 0 |
| TPPC5_HUMAN | TRAPPC5 | 21 kDa | 7 | 0 |
| UFM1_HUMAN | UFM1 | 9 kDa | 0 | 0 |
| VATF_HUMAN | ATP6V1F | 13 kDa | 0 | 0 |
| VATG1_HUMAN | ATP6V1G1 | 14 kDa | 0 | 8 |
| DESI1_HUMAN | DESI1 | 18 kDa | 3 | 0 |
| 1433E_HUMAN | YWHAE | 29 kDa | 4 | 1 |
| MA2B1_HUMAN | MAN2B1 | 114 kDa | 2 | 2 |
| LDHA_HUMAN | LDHA | 37 kDa | 6 | 2 |
| I2BP2_HUMAN | IRF2BP2 | 61 kDa | 4 | 4 |
| DBNL_HUMAN | DBNL | 48 kDa | 2 | 6 |
| DPY30_HUMAN | DPY30 | 11 kDa | 0 | 5 |
| MGST2_HUMAN | MGST2 | 17 kDa | 0 | 3 |
| NAAA_HUMAN | NAAA | 40 kDa | 0 | 4 |
| MED10_HUMAN | MED10 | 16 kDa | 0 | 3 |
| COA6_HUMAN (+1) | COA6 | 14 kDa | 0 | 0 |
| EMC6_HUMAN | EMC6 | 12 kDa | 0 | 0 |
| CDN2C_HUMAN | CDKN2C | 18 kDa | 1 | 5 |
| ATIF1_HUMAN | ATPIF1 | 12 kDa | 0 | 0 |
| DPM3_HUMAN | DPM3 | 10 kDa | 0 | 0 |
| DDA1_HUMAN | DDA1 | 12 kDa | 0 | 5 |
| RABP1_HUMAN | CRABP1 | 16 kDa | 0 | 7 |
| E9PKT9_HUMAN (+1) | MOCS2 | 9 kDa | 0 | 0 |
| K7EMR7_HUMAN (+1) | RTN2 | 30 kDa | 1 | 6 |
| VAPB_HUMAN | VAPB | 27 kDa | 0 | 0 |
| UCRIL_HUMAN (+1) | UQCRFS1P1 | 31 kDa | 1 | 0 |
| PTPS_HUMAN | PTS | 16 kDa | 0 | 3 |
| ARL8B_HUMAN | ARL8B | 22 kDa | 21 | 0 |
| B8ZZN6_HUMAN (+2) | SUMO1 | 17 kDa | 0 | 6 |
| BLVRB_HUMAN | BLVRB | 22 kDa | 7 | 0 |
| CBX3_HUMAN | CBX3 | 21 kDa | 8 | 0 |
| CHAC2_HUMAN | CHAC2 | 21 kDa | 0 | 8 |
| COMD5_HUMAN | COMMD5 | 25 kDa | 7 | 0 |
| COMD8_HUMAN | COMMD8 | 21 kDa | 8 | 0 |
| EMC8_HUMAN | EMC8 | 24 kDa | 7 | 0 |
| GEMI6_HUMAN | GEMIN6 | 19 kDa | 8 | 0 |
| GEMI7_HUMAN | GEMIN7 | 15 kDa | 0 | 7 |
| H0YNP9_HUMAN (+1) | HDDC3 | 16 kDa | 8 | 0 |
| LEGL_HUMAN | LGALSL | 19 kDa | 6 | 0 |
| LRC20_HUMAN | LRRC20 | 21 kDa | 5 | 0 |
| MIEN1_HUMAN | MIEN1 | 12 kDa | 0 | 0 |
| NATD1_HUMAN | NATD1 | 13 kDa | 0 | 0 |
| NDUB1_HUMAN | NDUFB1 | 7 kDa | 0 | 0 |
| Q9BRX8\|PXL2A_HUMAN | | ? | 7 | 0 |
| Q9NWV4\|CZIB_HUMAN | | ? | 7 | 0 |
| RAP2B_HUMAN | RAP2B | 21 kDa | 8 | 0 |
| RASN_HUMAN | NRAS | 21 kDa | 8 | 0 |
| RM40_HUMAN | MRPL40 | 24 kDa | 5 | 0 |
| RT11_HUMAN | MRPS11 | 21 kDa | 8 | 0 |
| RT16_HUMAN | MRPS16 | 15 kDa | 0 | 5 |
| TBPL1_HUMAN | TBPL1 | 21 kDa | 8 | 0 |
| TFAM_HUMAN | TFAM | 29 kDa | 6 | 0 |
| UBE2H_HUMAN | UBE2H | 21 kDa | 6 | 0 |
| UBL5_HUMAN | UBL5 | 9 kDa | 0 | 0 |
| J3KPP0_HUMAN (+3) | MRPL42 | 16 kDa | 0 | 3 |
| SC61G_HUMAN | SEC61G | 8 kDa | 0 | 0 |
| CL073_HUMAN | C12orf73 | 8 kDa | 0 | 0 |
| D6RDG3_HUMAN | BTF3 | 12 kDa | 17 | 0 |
| B4DFG0_HUMAN (+1) | DEK | 40 kDa | 1 | 1 |
| E9PL09_HUMAN (+1) | RPS3 | 25 kDa | 1 | 2 |
| S10A8_HUMAN | S100A8 | 11 kDa | 1 | 1 |
| IF4H_HUMAN | EIF4H | 27 kDa | 4 | 1 |
| CC124_HUMAN | CCDC124 | 26 kDa | 0 | 1 |
| CHCH1_HUMAN | CHCHD1 | 13 kDa | 0 | 2 |
| T2FB_HUMAN | GTF2F2 | 28 kDa | 3 | 1 |
| sp\|HBA_HUMAN\| (+1) | | 15 kDa | 0 | 8 |
| RBX2_HUMAN | RNF7 | 13 kDa | 0 | 1 |
| R39L5_HUMAN (+1) | RPL39P5 | 6 kDa | 1 | 0 |
| SRSF3_HUMAN | SRSF3 | 19 kDa | 2 | 1 |
| sp\|LYSC_HUMAN\| (+1) | | 17 kDa | 0 | 3 |
| IFM1_HUMAN | IFITM1 | 14 kDa | 0 | 0 |
| A0A087WVI1_HUMAN (+2) | PDLIM2 | 35 kDa | 1 | 4 |
| M0R226_HUMAN (+1) | MRPL34 | 20 kDa | 0 | 0 |
| BET1L_HUMAN | BET1L | 12 kDa | 0 | 0 |
| CD59_HUMAN (+3) | CD59 | 14 kDa | 4 | 3 |
| FXYD6_HUMAN | FXYD6 | 11 kDa | 0 | 4 |
| RIFK_HUMAN | RFK | 18 kDa | 5 | 2 |
| ACBD5_HUMAN (+1) | ACBD5 | 60 kDa | 2 | 3 |
| GCSH_HUMAN | GCSH | 19 kDa | 0 | 2 |
| E7ETY2_HUMAN (+5) | TCOF1 | 152 kDa | 4 | 2 |
| CTF8_HUMAN | CHTF8 | 13 kDa | 0 | 2 |
| COX7R_HUMAN | COX7A2L | 13 kDa | 0 | 0 |
| WRB_HUMAN | WRB | 20 kDa | 2 | 4 |
| T2AG_HUMAN | GTF2A2 | 12 kDa | 0 | 0 |
| TOM20_HUMAN | TOMM20 | 16 kDa | 0 | 4 |
| AP3S2_HUMAN (+1) | AP3S2 | 22 kDa | 3 | 2 |
| TCPD_HUMAN | CCT4 | 58 kDa | 5 | 1 |
| TX1B3_HUMAN | TAX1BP3 | 14 kDa | 0 | 5 |
| Q9H5X1\|CIA2A_HUMAN | | ? | 5 | 1 |
| THIOM_HUMAN | TXN2 | 18 kDa | 0 | 1 |
| COXM1_HUMAN | CMC1 | 12 kDa | 0 | 1 |
| ATRAP_HUMAN | AGTRAP | 17 kDa | 5 | 1 |
| 4EBP1_HUMAN | EIF4EBP1 | 13 kDa | 2 | 0 |
| S10AD_HUMAN | S100A13 | 11 kDa | 0 | 0 |
| EMRE_HUMAN | SMDT1 | 11 kDa | 0 | 0 |
| TPC6A_HUMAN | TRAPPC6A | 18 kDa | 1 | 5 |
| OPA3_HUMAN | OPA3 | 20 kDa | 4 | 1 |
| RM33_HUMAN | MRPL33 | 8 kDa | 0 | 0 |
| AIMP1_HUMAN (+1) | AIMP1 | 34 kDa | 0 | 0 |
| F8W7C6_HUMAN | RPL10 | 19 kDa | 6 | 0 |
| STML2_HUMAN | STOML2 | 39 kDa | 5 | 0 |
| E9PN70_HUMAN (+1) | TRAPPC4 | 29 kDa | 5 | 0 |
| A0A0C4DFM0_HUMAN (+1) | GSTZ1 | 24 kDa | 5 | 0 |
| AP1S1_HUMAN (+2) | AP1S1 | 19 kDa | 9 | 0 |
| ARFRP_HUMAN | ARFRP1 | 23 kDa | 7 | 0 |
| BL1S2_HUMAN | BLOC1S2 | 16 kDa | 6 | 0 |
| CETN3_HUMAN (+2) | CETN3 | 20 kDa | 7 | 0 |
| D6RCM8_HUMAN (+1) | TNFAIP8 | 23 kDa | 5 | 0 |
| DOPP1_HUMAN | DOLPP1 | 27 kDa | 6 | 0 |
| E7EMS2_HUMAN (+4) | NPC2 | 22 kDa | 5 | 0 |
| EBP_HUMAN | EBP | 26 kDa | 7 | 0 |
| FHIT_HUMAN | FHIT | 17 kDa | 6 | 0 |
| ISCA1_HUMAN | ISCA1 | 14 kDa | 0 | 5 |
| ITPA_HUMAN | ITPA | 21 kDa | 6 | 0 |
| K1143_HUMAN | KIAA1143 | 17 kDa | 7 | 0 |
| MAIP1_HUMAN | MAIP1 | 33 kDa | 5 | 0 |
| MED11_HUMAN | MED11 | 13 kDa | 0 | 0 |
| MED18_HUMAN | MED18 | 24 kDa | 5 | 0 |
| MTFP1_HUMAN | MTFP1 | 18 kDa | 7 | 0 |
| MYCBP_HUMAN | MYCBP | 12 kDa | 0 | 0 |
| NCAS2_HUMAN | NCBP2-AS2 | 11 kDa | 0 | 0 |
| NXT1_HUMAN | NXT1 | 16 kDa | 0 | 7 |
| PKHJ1_HUMAN | PLEKHJ1 | 18 kDa | 0 | 5 |
| PSF2_HUMAN | GINS2 | 21 kDa | 6 | 0 |
| RAB7L_HUMAN | RAB29 | 23 kDa | 6 | 0 |
| RB22A_HUMAN | RAB22A | 22 kDa | 8 | 0 |
| RM53_HUMAN | MRPL53 | 12 kDa | 0 | 0 |
| RP25L_HUMAN | RPP25L | 18 kDa | 5 | 0 |
| RPB11_HUMAN | POLR2J | 13 kDa | 0 | 0 |
| RPB9_HUMAN | POLR2I | 15 kDa | 0 | 5 |
| RPE_HUMAN | RPE | 25 kDa | 7 | 0 |
| RS6_HUMAN | RPS6 | 29 kDa | 7 | 0 |
| TBCB_HUMAN | TBCB | 27 kDa | 0 | 5 |
| TMM33_HUMAN | TMEM33 | 28 kDa | 3 | 0 |
| UQCC2_HUMAN (+1) | UQCC2 | 15 kDa | 0 | 7 |
| PRDX4_HUMAN | PRDX4 | 31 kDa | 12 | 0 |
| K7ELQ9_HUMAN (+1) | TMEM205 | 19 kDa | 5 | 0 |
| Q9HB66_HUMAN | MKKS | 7 kDa | 0 | 0 |
| TP4A1_HUMAN | PTP4A1 | 20 kDa | 12 | 0 |
| RRP1B_HUMAN | RRP1B | 84 kDa | 1 | 2 |
| UBQL2_HUMAN | UBQLN2 | 66 kDa | 2 | 2 |
| H0YHG0_HUMAN | | 59 kDa | 0 | 2 |
| J3KTE4_HUMAN (+2) | RPL19 | 23 kDa | 0 | 1 |
| A0A087WYF8_HUMAN (+2) | PDLIM3 | 22 kDa | 1 | 3 |
| LYRIC_HUMAN | MTDH | 64 kDa | 3 | 2 |
| H2B2C_HUMAN (+1) | HIST2H2BC | 21 kDa | 4 | 1 |
| CSRP1_HUMAN | CSRP1 | 21 kDa | 4 | 0 |
| E9PNK6_HUMAN (+3) | TPD52L1 | 19 kDa | 3 | 2 |
| RLA0_HUMAN | RPLP0 | 34 kDa | 2 | 4 |
| ASGL1_HUMAN | ASRGL1 | 32 kDa | 2 | 2 |
| E7ES96_HUMAN | PSEN1 | 42 kDa | 2 | 3 |
| TIF1B_HUMAN | TRIM28 | 89 kDa | 0 | 4 |
| D6REX3_HUMAN (+1) | SEC31A | 136 kDa | 2 | 2 |
| A6NNI4_HUMAN (+3) | CD9 | 18 kDa | 3 | 2 |
| ACYP2_HUMAN | ACYP2 | 11 kDa | 0 | 0 |
| SFT2A_HUMAN | SFT2D1 | 18 kDa | 0 | 2 |
| RPB4_HUMAN | POLR2D | 16 kDa | 1 | 4 |
| TXN4B_HUMAN | TXNL4B | 17 kDa | 1 | 2 |
| CHERP_HUMAN (+1) | CHERP | 104 kDa | 0 | 0 |
| LGUL_HUMAN | GLO1 | 21 kDa | 3 | 1 |
| TOM7_HUMAN | TOMM7 | 6 kDa | 0 | 0 |
| QCR9_HUMAN | UQCR10 | 7 kDa | 0 | 1 |
| A0A075B6E5_HUMAN (+2) | ENAH | 25 kDa | 0 | 2 |
| ARL5A_HUMAN | ARL5A | 21 kDa | 1 | 3 |
| F5GZY7_HUMAN (+1) | GABARAPL1 | 9 kDa | 0 | 6 |
| E9PLI6_HUMAN (+2) | EIF1AD | 13 kDa | 5 | 1 |
| RM51_HUMAN | MRPL51 | 15 kDa | 0 | 1 |
| IPYR_HUMAN | PPA1 | 33 kDa | 4 | 1 |
| SGMR1_HUMAN | SIGMAR1 | 25 kDa | 4 | 0 |
| PRAF2_HUMAN | PRAF2 | 19 kDa | 1 | 5 |
| C9JYM0_HUMAN (+1) | POP7 | 15 kDa | 5 | 1 |
| MPC1_HUMAN | MPC1 | 12 kDa | 1 | 0 |
| MGN_HUMAN | MAGOH | 17 kDa | 24 | 18 |
| RPC9_HUMAN | CRCP | 17 kDa | 4 | 1 |
| XRCC5_HUMAN | XRCC5 | 83 kDa | 5 | 1 |
| A0A087X1A5_HUMAN (+2) | STAU1 | 55 kDa | 1 | 4 |
| PDZ11_HUMAN | PDZD11 | 16 kDa | 5 | 0 |
| NDUBB_HUMAN | NDUFB11 | 17 kDa | 0 | 4 |
| A0A087WUW9_HUMAN (+1) | ARL15 | 23 kDa | 6 | 0 |
| A0A0C4DG91_HUMAN (+4) | NME6 | 22 kDa | 4 | 0 |
| ACOX1_HUMAN | ACOX1 | 74 kDa | 5 | 0 |
| BORC7_HUMAN | BORCS7 | 12 kDa | 0 | 0 |
| C9J1C6_HUMAN (+1) | RNF181 | 16 kDa | 5 | 0 |
| CETN2_HUMAN | CETN2 | 20 kDa | 5 | 0 |
| CHCH2_HUMAN (+1) | CHCHD2 | 16 kDa | 6 | 0 |
| COX20_HUMAN | COX20 | 13 kDa | 0 | 5 |
| F5GYJ5_HUMAN (+1) | SDHAF2 | 18 kDa | 0 | 6 |
| GBG12_HUMAN | GNG12 | 8 kDa | 0 | 0 |
| GBG4_HUMAN | GNG4 | 8 kDa | 0 | 0 |
| GFRP_HUMAN | GCHFR | 10 kDa | 0 | 0 |
| I3L4Z9_HUMAN (+1) | SCPEP1 | 15 kDa | 5 | 0 |
| J3KSP0_HUMAN (+1) | SKA2 | 13 kDa | 0 | 4 |
| NAA10_HUMAN | NAA10 | 26 kDa | 6 | 0 |
| NIP7_HUMAN | NIP7 | 20 kDa | 6 | 0 |
| PPM1G_HUMAN | PPM1G | 59 kDa | 5 | 0 |
| RB11B_HUMAN | RAB11B | 24 kDa | 6 | 0 |
| SPCS1_HUMAN | SPCS1 | 12 kDa | 0 | 0 |
| TIM21_HUMAN | TIMM21 | 28 kDa | 5 | 0 |
| TMED3_HUMAN | TMED3 | 25 kDa | 6 | 0 |
| TOM34_HUMAN | TOMM34 | 35 kDa | 6 | 0 |
| VISL1_HUMAN | VSNL1 | 22 kDa | 6 | 0 |
| A0A096LP10_HUMAN | ALG13 | 18 kDa | 5 | 0 |
| COMD6_HUMAN | COMMD6 | 10 kDa | 0 | 0 |
| RT12_HUMAN | MRPS12 | 15 kDa | 0 | 5 |
| H3BN98_HUMAN | | 27 kDa | 8 | 0 |
| TPC2A_HUMAN (+1) | TRAPPC2 | 16 kDa | 0 | 5 |
| COX19_HUMAN | COX19 | 10 kDa | 0 | 0 |
| G3V5L1_HUMAN (+1) | MAX | 14 kDa | 5 | 0 |
| A6NGJ0_HUMAN (+1) | DYNLT3 | 17 kDa | 0 | 0 |
| FITM2_HUMAN | FITM2 | 30 kDa | 2 | 0 |
| ACBD7_HUMAN | ACBD7 | 10 kDa | 0 | 0 |
| COA1_HUMAN | COA1 | 17 kDa | 0 | 3 |
| MALD1_HUMAN | MARVELD1 | 19 kDa | 0 | 4 |
| DNJC5_HUMAN | DNAJC5 | 22 kDa | 4 | 0 |
| UQCC3_HUMAN | UQCC3 | 10 kDa | 0 | 0 |
| DHSD_HUMAN | SDHD | 17 kDa | 0 | 0 |
| RL13A_HUMAN | RPL13A | 24 kDa | 1 | 1 |
| PTMS_HUMAN | PTMS | 12 kDa | 3 | 0 |
| F6XHT0_HUMAN (+1) | SELENOK | 10 kDa | 0 | 2 |
| MPCP_HUMAN | SLC25A3 | 40 kDa | 1 | 1 |
| H12_HUMAN (+1) | HIST1H1C | 21 kDa | 3 | 1 |
| LASP1_HUMAN | LASP1 | 30 kDa | 0 | 1 |
| ADT2_HUMAN (+1) | SLC25A5 | 33 kDa | 1 | 0 |
| H0Y861_HUMAN (+4) | TMEM222 | 18 kDa | 3 | 2 |
| TM147_HUMAN | TMEM147 | 25 kDa | 2 | 2 |
| EIF1B_HUMAN | EIF1B | 13 kDa | 0 | 10 |
| A6NGP5_HUMAN (+5) | JPT2 | 19 kDa | 2 | 3 |
| A0A0A0MSQ0_HUMAN (+1) | PLS3 | 69 kDa | 3 | 2 |
| H0Y4R1_HUMAN (+1) | IMPDH2 | 51 kDa | 0 | 2 |
| A0A087X1G7_HUMAN (+1) | SELENOF | 18 kDa | 0 | 3 |
| STX7_HUMAN | STX7 | 30 kDa | 3 | 2 |
| RM30_HUMAN | MRPL30 | 19 kDa | 2 | 2 |
| S10AA_HUMAN | S100A10 | 11 kDa | 0 | 0 |
| CC167_HUMAN | CCDC167 | 11 kDa | 0 | 0 |
| A0A087WUI2_HUMAN (+1) | HNRNPA2B1 | 30 kDa | 2 | 0 |
| TOM6_HUMAN | TOMM6 | 8 kDa | 0 | 0 |
| A0A0U1RRM4_HUMAN (+2) | PTBP1 | 62 kDa | 1 | 2 |
| NDUV3_HUMAN | NDUFV3 | 12 kDa | 0 | 0 |
| NC2B_HUMAN | DR1 | 19 kDa | 4 | 1 |
| CIRBP_HUMAN | CIRBP | 19 kDa | 4 | 1 |
| SRXN1_HUMAN | SRXN1 | 14 kDa | 0 | 1 |
| G3XAN8_HUMAN (+1) | TIMM8B | 11 kDa | 0 | 0 |
| FKB1B_HUMAN | FKBP1B | 12 kDa | 0 | 1 |
| RBM14_HUMAN | RBM14 | 69 kDa | 0 | 1 |
| H0YMV8_HUMAN (+1) | RPS27L | 11 kDa | 0 | 6 |
| PROF2_HUMAN | PFN2 | 15 kDa | 0 | 12 |
| A0A0C4DFX9_HUMAN (+1) | NELFA | 59 kDa | 0 | 3 |
| AT5EL_HUMAN (+1) | ATP5EP2 | 6 kDa | 0 | 0 |
| KISHA_HUMAN | TMEM167A | 8 kDa | 0 | 0 |
| ICT1_HUMAN (+1) | MRPL58 | 24 kDa | 4 | 0 |
| COXM2_HUMAN | CMC2 | 9 kDa | 0 | 0 |
| TMM11_HUMAN | TMEM11 | 22 kDa | 2 | 0 |
| A0A087WUC6_HUMAN (+2) | SPCS2 | 25 kDa | 4 | 0 |
| A0A087WYU2_HUMAN (+1) | METTL26 | 20 kDa | 5 | 0 |
| ADAT2_HUMAN | ADAT2 | 21 kDa | 5 | 0 |
| ASF1B_HUMAN | ASF1B | 22 kDa | 5 | 0 |
| BAG2_HUMAN | BAG2 | 24 kDa | 4 | 0 |
| CB076_HUMAN | C2orf76 | 15 kDa | 0 | 4 |
| CHRC1_HUMAN | CHRAC1 | 15 kDa | 0 | 5 |
| CKS2_HUMAN | CKS2 | 10 kDa | 0 | 0 |
| DCNL5_HUMAN | DCUN1D5 | 28 kDa | 4 | 0 |
| E5RGR0_HUMAN (+1) | LYPLA1 | 21 kDa | 5 | 0 |
| EMC4_HUMAN (+2) | EMC4 | 20 kDa | 5 | 0 |
| F5H0U5_HUMAN (+1) | GLTP | 22 kDa | 5 | 0 |
| GALD1_HUMAN | GATD1 | 23 kDa | 5 | 0 |
| GBG2_HUMAN | GNG2 | 8 kDa | 0 | 0 |
| GSKIP_HUMAN | GSKIP | 16 kDa | 0 | 5 |
| HEBP1_HUMAN | HEBP1 | 21 kDa | 5 | 0 |
| MCRI2_HUMAN | MCRIP2 | 18 kDa | 3 | 0 |
| MSRB2_HUMAN | MSRB2 | 20 kDa | 5 | 0 |
| MSS4_HUMAN | RABIF | 14 kDa | 0 | 5 |
| NPM3_HUMAN | NPM3 | 19 kDa | 5 | 0 |
| NUCL_HUMAN | NCL | 77 kDa | 4 | 0 |
| PAIP2_HUMAN | PAIP2 | 15 kDa | 5 | 0 |
| PPGB_HUMAN (+2) | CTSA | 54 kDa | 3 | 0 |
| Q9Y5N5\|N6MT1_HUMAN | | ? | 5 | 0 |
| RAP2C_HUMAN | RAP2C | 21 kDa | 6 | 0 |
| RBM25_HUMAN | RBM25 | 100 kDa | 5 | 0 |
| SPCS3_HUMAN | SPCS3 | 20 kDa | 5 | 0 |
| TC1D2_HUMAN | TCTEX1D2 | 16 kDa | 0 | 5 |
| TTC9C_HUMAN | TTC9C | 20 kDa | 4 | 0 |
| UXT_HUMAN | UXT | 18 kDa | 5 | 0 |
| UB2G2_HUMAN | UBE2G2 | 19 kDa | 5 | 0 |
| DERL2_HUMAN | DERL2 | 28 kDa | 4 | 0 |
| LLPH_HUMAN | LLPH | 15 kDa | 4 | 0 |
| HSDL2_HUMAN | HSDL2 | 45 kDa | 0 | 4 |
| PDLI5_HUMAN | PDLIM5 | 64 kDa | 0 | 3 |
| RAB6B_HUMAN | RAB6B | 23 kDa | 8 | 0 |
| COMD7_HUMAN (+4) | COMMD7 | 23 kDa | 4 | 0 |
| Q9BTL3\|RAMAC_HUMAN | | ? | 0 | 4 |
| TMM70_HUMAN | TMEM70 | 29 kDa | 4 | 0 |
| TIFA_HUMAN | TIFA | 21 kDa | 3 | 0 |
| IFG15_HUMAN | TOR1AIP2 | 15 kDa | 0 | 4 |
| A0A0A0MSK2_HUMAN (+1) | PET100 | 6 kDa | 0 | 0 |
| RM32_HUMAN | MRPL32 | 21 kDa | 0 | 5 |
| I3L295_HUMAN (+5) | MPDU1 | 12 kDa | 4 | 0 |
| A0A087WYP2_HUMAN (+1) | SELENOH | 13 kDa | 0 | 3 |
| COX16_HUMAN | COX16 | 12 kDa | 0 | 0 |
| C9JY04_HUMAN (+1) | TMEM243 | 13 kDa | 0 | 0 |
| CD003_HUMAN | C4orf3 | 8 kDa | 0 | 0 |
| A0A0C4DG17_HUMAN (+3) | RPSA | 33 kDa | 1 | 1 |
| EF2_HUMAN | EEF2 | 95 kDa | 1 | 1 |
| KCRB_HUMAN | CKB | 43 kDa | 0 | 1 |
| A0A0A0MRM9_HUMAN (+1) | NOLC1 | 75 kDa | 1 | 1 |
| AROS_HUMAN | RPS19BP1 | 15 kDa | 2 | 0 |
| PSIP1_HUMAN | PSIP1 | 60 kDa | 2 | 1 |
| C9JA28_HUMAN (+1) | SSR3 | 20 kDa | 2 | 1 |
| ROMO1_HUMAN | ROMO1 | 8 kDa | 0 | 1 |
| IMA1_HUMAN (+2) | KPNA2 | 58 kDa | 0 | 1 |
| K7EJV0_HUMAN (+3) | sept-09 | 14 kDa | 0 | 1 |
| LS14A_HUMAN | LSM14A | 51 kDa | 1 | 0 |
| G3V438_HUMAN | AHSA1 | 23 kDa | 2 | 2 |
| Q5TIH2_HUMAN (+1) | SFT2D2 | 12 kDa | 0 | 2 |
| PSB7_HUMAN | PSMB7 | 30 kDa | 2 | 0 |
| QCR6_HUMAN | UQCRH | 11 kDa | 0 | 0 |
| SPT5H_HUMAN | SUPT5H | 121 kDa | 2 | 2 |
| AP1S3_HUMAN (+1) | AP1S3 | 18 kDa | 3 | 3 |
| A0A0A0MRX2_HUMAN (+1) | DDRGK1 | 34 kDa | 0 | 0 |
| P5CS_HUMAN | ALDH18A1 | 87 kDa | 2 | 0 |
| MCM6_HUMAN | MCM6 | 93 kDa | 2 | 1 |
| B5MCD7_HUMAN (+1) | SYNGR1 | 18 kDa | 2 | 1 |
| RBM12_HUMAN | RBM12 | 97 kDa | 0 | 1 |
| CENPS_HUMAN | CENPS | 16 kDa | 1 | 1 |
| DNMT1_HUMAN | DNMT1 | 183 kDa | 2 | 0 |
| IF2B1_HUMAN | IGF2BP1 | 63 kDa | 2 | 0 |
| LSM10_HUMAN | LSM10 | 14 kDa | 0 | 2 |
| RL15_HUMAN | RPL15 | 24 kDa | 2 | 1 |
| A7L3B_HUMAN | ATXN7L3B | 11 kDa | 0 | 0 |
| CRIPT_HUMAN | CRIPT | 11 kDa | 0 | 2 |
| A0A0D9SF63_HUMAN (+4) | TBL1XR1 | 52 kDa | 0 | 1 |
| 1433Z_HUMAN | YWHAZ | 28 kDa | 3 | 1 |
| VDAC1_HUMAN | VDAC1 | 31 kDa | 0 | 1 |
| RMI2_HUMAN | RMI2 | 16 kDa | 2 | 1 |
| CISD3_HUMAN | CISD3 | 14 kDa | 0 | 2 |
| Q5W0S5_HUMAN (+1) | RAD23B | 15 kDa | 0 | 0 |
| SI11A_HUMAN | SMIM11A | 7 kDa | 0 | 0 |
| sp\|NEDD8_HUMAN\| (+1) | | 9 kDa | 0 | 0 |
| 1433T_HUMAN (+1) | YWHAQ | 28 kDa | 1 | 3 |
| LAP2A_HUMAN | TMPO | 75 kDa | 4 | 3 |
| JTB_HUMAN | JTB | 16 kDa | 1 | 3 |
| B5MC53_HUMAN (+3) | MPV17 | 19 kDa | 1 | 3 |
| E7EVA0_HUMAN (+1) | MAP4 | 245 kDa | 2 | 0 |
| E9PLM6_HUMAN (+2) | MDK | 17 kDa | 1 | 2 |
| A0A0A0MR02_HUMAN (+1) | VDAC2 | 30 kDa | 0 | 2 |
| ALDOA_HUMAN (+3) | ALDOA | 39 kDa | 1 | 0 |
| NDK8_HUMAN | NME2P1 | 16 kDa | 49 | 39 |
| L10K_HUMAN | C19orf53 | 11 kDa | 1 | 2 |
| CSDE1_HUMAN | CSDE1 | 89 kDa | 1 | 2 |
| MOFA1_HUMAN | MRFAP1 | 15 kDa | 0 | 3 |
| MAP1B_HUMAN | MAP1B | 271 kDa | 2 | 1 |
| RRFM_HUMAN | MRRF | 29 kDa | 2 | 0 |
| RTN3_HUMAN | RTN3 | 113 kDa | 3 | 1 |
| DNS2A_HUMAN (+1) | DNASE2 | 40 kDa | 0 | 0 |
| F5H6E4_HUMAN (+1) | ANAPC15 | 14 kDa | 1 | 1 |
| H3BTL1_HUMAN (+2) | MAP1LC3B | 9 kDa | 0 | 3 |
| ERG28_HUMAN | ERG28 | 16 kDa | 0 | 1 |
| H3BQ52_HUMAN | ARPP19 | 8 kDa | 3 | 2 |
| F6VRR5_HUMAN (+1) | POLDIP3 | 48 kDa | 3 | 1 |
| IMB1_HUMAN (+1) | KPNB1 | 97 kDa | 2 | 0 |
| B7Z306_HUMAN (+4) | UBE2E1 | 19 kDa | 2 | 1 |
| RT63_HUMAN | MRPL57 | 12 kDa | 1 | 0 |
| DNLI3_HUMAN | LIG3 | 113 kDa | 1 | 2 |
| OXLD1_HUMAN | OXLD1 | 16 kDa | 0 | 0 |
| G3XAL0_HUMAN (+1) | MDH2 | 25 kDa | 3 | 0 |
| PAPOA_HUMAN | PAPOLA | 83 kDa | 3 | 0 |
| sp\|P62979\|RS27A_HUMAN Ubiquitin-40S ribosomal protein S27a | | 18 kDa | 0 | 18 |
| A0A0A0MRR3_HUMAN | TAF8 | 19 kDa | 3 | 0 |
| ARP5L_HUMAN | ARPC5L | 17 kDa | 4 | 0 |
| ASF1A_HUMAN | ASF1A | 23 kDa | 5 | 0 |
| ATP5S_HUMAN (+1) | ATP5S | 25 kDa | 3 | 0 |
| CENPM_HUMAN | CENPM | 20 kDa | 3 | 0 |
| CH082_HUMAN | C8orf82 | 24 kDa | 4 | 0 |
| CNN3_HUMAN | CNN3 | 36 kDa | 4 | 0 |
| CYBP_HUMAN | CACYBP | 26 kDa | 4 | 0 |
| D6RC52_HUMAN (+3) | NHP2 | 15 kDa | 4 | 0 |
| F8VZJ2_HUMAN (+4) | NACA | 15 kDa | 3 | 0 |
| JUPI1_HUMAN | JPT1 | 16 kDa | 4 | 0 |
| M0R3D4_HUMAN (+1) | RABAC1 | 17 kDa | 3 | 0 |
| MOG1_HUMAN | RANGRF | 20 kDa | 4 | 0 |
| ORML1_HUMAN | ORMDL1 | 17 kDa | 0 | 5 |
| Q5T8U5_HUMAN (+1) | SURF4 | 21 kDa | 4 | 0 |
| RAB4B_HUMAN | RAB4B | 24 kDa | 10 | 0 |
| RET7_HUMAN | RBP7 | 16 kDa | 0 | 4 |
| RT26_HUMAN | MRPS26 | 24 kDa | 4 | 0 |
| SEN15_HUMAN | TSEN15 | 19 kDa | 4 | 0 |
| SYYC_HUMAN | YARS | 59 kDa | 4 | 0 |
| TM41A_HUMAN | TMEM41A | 30 kDa | 3 | 0 |
| TRIA1_HUMAN | TRIAP1 | 9 kDa | 0 | 0 |
| VAMP4_HUMAN | VAMP4 | 16 kDa | 0 | 4 |
| YPEL5_HUMAN | YPEL5 | 14 kDa | 0 | 0 |
| CENPX_HUMAN (+1) | CENPX | 9 kDa | 0 | 0 |
| D6R9K7_HUMAN (+3) | RBM4 | 17 kDa | 0 | 4 |
| DCTN5_HUMAN (+1) | DCTN5 | 20 kDa | 3 | 0 |
| MTURN_HUMAN | MTURN | 15 kDa | 0 | 3 |
| SPRY7_HUMAN | SPRYD7 | 22 kDa | 3 | 0 |
| sp\|O76070\|SYUG_HUMAN Gamma-synuclein OS=Homo sapiens GN=SNCG PE=1 SV=2 | | 13 kDa | 0 | 4 |
| sp\|P08238\|HS90B_HUMAN Heat shock protein HSP 90-beta OS=Homo sapiens GN=HSP90AB1 PE=1 SV=4 | | 83 kDa | 0 | 4 |
| COAC_HUMAN (+1) | PPCDC | 22 kDa | 4 | 0 |
| COMT_HUMAN | COMT | 30 kDa | 3 | 0 |
| E9PC52_HUMAN (+1) | RBBP7 | 47 kDa | 4 | 0 |
| PLP2_HUMAN | PLP2 | 17 kDa | 0 | 4 |
| RHOF_HUMAN | RHOF | 24 kDa | 4 | 0 |
| SAT2_HUMAN | SAT2 | 19 kDa | 0 | 4 |
| SELS_HUMAN | SELENOS | 21 kDa | 3 | 0 |
| K4DI92_HUMAN (+1) | RWDD4 | 21 kDa | 3 | 0 |
| COX14_HUMAN | COX14 | 7 kDa | 0 | 0 |
| AP5S1_HUMAN | AP5S1 | 23 kDa | 3 | 0 |
| A0A087WVA1_HUMAN (+1) | SELENOT | 22 kDa | 0 | 3 |
| MBLC2_HUMAN | MBLAC2 | 31 kDa | 4 | 0 |
| YBEY_HUMAN | YBEY | 19 kDa | 3 | 0 |
| GL1AD_HUMAN | POLR2M | 15 kDa | 0 | 4 |
| F2Z3L6_HUMAN (+1) | GUSB | 17 kDa | 0 | 3 |
| CPTP_HUMAN | CPTP | 24 kDa | 3 | 0 |
| VHL_HUMAN | VHL | 24 kDa | 3 | 0 |
| USE1_HUMAN | USE1 | 29 kDa | 0 | 3 |
| ALG14_HUMAN | ALG14 | 24 kDa | 2 | 0 |
| MIDUO_HUMAN | MIEF1 | 8 kDa | 0 | 0 |
| A0A1W2PPS1_HUMAN (+2) | HNRNPU | 88 kDa | 0 | 0 |
| DUS23_HUMAN | DUSP23 | 17 kDa | 0 | 2 |
| CDC26_HUMAN | CDC26 | 10 kDa | 0 | 0 |
| CAR19_HUMAN | CARD19 | 26 kDa | 3 | 0 |
| CS025_HUMAN (+1) | C19orf25 | 13 kDa | 0 | 2 |
| A0A3B3IRV3\|A0A3B3IRV3_HUMAN | | ? | 16 | 0 |
| RAP1A_HUMAN | RAP1A | 21 kDa | 16 | 0 |
| SFT2C_HUMAN | SFT2D3 | 22 kDa | 3 | 0 |
| UBE2B_HUMAN | UBE2B | 17 kDa | 0 | 8 |
| TM223_HUMAN | TMEM223 | 22 kDa | 3 | 0 |
| GBG7_HUMAN | GNG7 | 8 kDa | 0 | 0 |
| J3KRR0_HUMAN (+2) | LIMD2 | 10 kDa | 3 | 0 |
| A0A0C4DGJ7_HUMAN (+1) | CHURC1 | 13 kDa | 0 | 0 |
| SOSSC_HUMAN | INIP | 11 kDa | 0 | 0 |
| RAC3_HUMAN | RAC3 | 21 kDa | 11 | 0 |
| RM35_HUMAN | MRPL35 | 22 kDa | 0 | 3 |
| GON7_HUMAN | GON7 | 11 kDa | 0 | 0 |
| C42S2_HUMAN | CDC42SE2 | 9 kDa | 0 | 0 |
| B9A057_HUMAN (+1) | COA5 | 8 kDa | 0 | 0 |
| RNF5_HUMAN | RNF5 | 20 kDa | 2 | 0 |
| A0A0A0MTS7_HUMAN | TTN | 3994 kDa | 0 | 3 |
| B0QYL8_HUMAN (+6) | POLR2F | 14 kDa | 0 | 2 |
| NU3M_HUMAN | MT-ND3 | 13 kDa | 0 | 0 |
| Q5VTR2\|BRE1A_HUMAN-DECOY | | ? | 0 | 1 |
| F5H6U7_HUMAN (+2) | GOLT1B | 14 kDa | 1 | 1 |
| RL10A_HUMAN | RPL10A | 25 kDa | 1 | 1 |
| IF2GL_HUMAN (+1) | EIF2S3L | 51 kDa | 0 | 1 |
| MOB1A_HUMAN | MOB1A | 25 kDa | 2 | 0 |
| HEXB_HUMAN | HEXB | 63 kDa | 0 | 0 |
| A0A087WTV2_HUMAN (+4) | PSME3 | 20 kDa | 2 | 0 |
| A6NJA2_HUMAN (+1) | USP14 | 51 kDa | 0 | 1 |
| RACK1_HUMAN | RACK1 | 35 kDa | 2 | 1 |
| B7Z7F3_HUMAN (+1) | RANBP3 | 53 kDa | 2 | 1 |
| CARF_HUMAN | CDKN2AIP | 61 kDa | 2 | 1 |
| TBA4A_HUMAN | TUBA4A | 50 kDa | 0 | 0 |
| B5MDF5_HUMAN (+2) | RAN | 26 kDa | 1 | 1 |
| GGACT_HUMAN | GGACT | 17 kDa | 1 | 2 |
| CLCA_HUMAN (+1) | CLTA | 27 kDa | 1 | 2 |
| Q5SXM7_HUMAN | DNLZ | 13 kDa | 1 | 2 |
| ACTA_HUMAN (+3) | ACTA2 | 42 kDa | 3 | 0 |
| MPPA_HUMAN | PMPCA | 58 kDa | 2 | 1 |
| PEN2_HUMAN | PSENEN | 12 kDa | 0 | 0 |
| EIF3F_HUMAN (+1) | EIF3F | 38 kDa | 1 | 0 |
| RL13_HUMAN | RPL13 | 24 kDa | 1 | 1 |
| H2A1A_HUMAN (+1) | HIST1H2AA | 14 kDa | 3 | 12 |
| GGH_HUMAN | GGH | 36 kDa | 0 | 0 |
| C9JJT5_HUMAN (+1) | ATP5J2-PTCD1 | 6 kDa | 0 | 0 |
| A0A286YF22_HUMAN (+3) | PHGDH | 56 kDa | 0 | 0 |
| E7EPN9_HUMAN (+1) | PRRC2C | 309 kDa | 0 | 0 |
| H2B1B_HUMAN (+3) | HIST1H2BB | 14 kDa | 10 | 0 |
| H0YA52_HUMAN (+1) | PCBD2 | 13 kDa | 0 | 0 |
| LMNB2_HUMAN | LMNB2 | 70 kDa | 2 | 0 |
| TAF12_HUMAN | TAF12 | 18 kDa | 1 | 2 |
| CCAR2_HUMAN (+4) | CCAR2 | 103 kDa | 2 | 1 |
| PCYOX_HUMAN | PCYOX1 | 57 kDa | 1 | 0 |
| C9JGV6_HUMAN (+3) | RANBP1 | 23 kDa | 2 | 1 |
| H1X_HUMAN | H1FX | 22 kDa | 2 | 1 |
| G3BP1_HUMAN | G3BP1 | 52 kDa | 2 | 1 |
| SVIP_HUMAN | SVIP | 8 kDa | 0 | 0 |
| CHTOP_HUMAN (+1) | CHTOP | 26 kDa | 0 | 2 |
| TM35B_HUMAN | TMEM35B | 17 kDa | 0 | 1 |
| AR6P6_HUMAN | ARL6IP6 | 25 kDa | 1 | 1 |
| SVBP_HUMAN | SVBP | 8 kDa | 0 | 0 |
| PLBL2_HUMAN | PLBD2 | 65 kDa | 0 | 1 |
| J3KP15_HUMAN (+3) | SRSF2 | 15 kDa | 0 | 1 |
| IF2B_HUMAN | EIF2S2 | 38 kDa | 0 | 1 |
| ARFG1_HUMAN (+4) | ARFGAP1 | 45 kDa | 1 | 2 |
| C9JCW7_HUMAN (+2) | TMUB1 | 18 kDa | 2 | 1 |
| E9PQD7_HUMAN (+2) | RPS2 | 25 kDa | 0 | 1 |
| B0YJC4_HUMAN (+1) | VIM | 50 kDa | 1 | 0 |
| B4DJK0_HUMAN (+2) | SRSF5 | 14 kDa | 1 | 0 |
| A0A1B0GV23_HUMAN (+6) | CTSD | 44 kDa | 0 | 1 |
| CPIN1_HUMAN (+1) | CIAPIN1 | 34 kDa | 2 | 0 |
| ATP6_HUMAN | MT-ATP6 | 25 kDa | 0 | 1 |
| KIF2C_HUMAN (+1) | KIF2C | 81 kDa | 1 | 0 |
| A0A0B4J297_HUMAN (+2) | NAA38 | 8 kDa | 0 | 2 |
| A0A0C4DFP4_HUMAN (+1) | NGLY1 | 72 kDa | 0 | 0 |
| BAP18_HUMAN (+1) | BAP18 | 18 kDa | 3 | 0 |
| CC115_HUMAN (+1) | CCDC115 | 20 kDa | 3 | 0 |
| F210B_HUMAN | FAM210B | 20 kDa | 0 | 3 |
| F5H872_HUMAN (+1) | MED21 | 17 kDa | 0 | 3 |
| PP1RB_HUMAN | PPP1R11 | 14 kDa | 3 | 0 |
| RAB38_HUMAN | RAB38 | 24 kDa | 4 | 0 |
| RFESD_HUMAN | RFESD | 18 kDa | 3 | 0 |
| A0A087WYV6_HUMAN (+2) | TSPAN6 | 15 kDa | 3 | 0 |
| CO061_HUMAN | C15orf61 | 18 kDa | 0 | 3 |
| M0R221_HUMAN (+2) | SNRPA | 16 kDa | 4 | 0 |
| DJC30_HUMAN | DNAJC30 | 26 kDa | 3 | 0 |
| K7ERI8_HUMAN (+2) | | 18 kDa | 3 | 0 |
| CAV1_HUMAN | CAV1 | 20 kDa | 3 | 0 |
| SYUB_HUMAN | SNCB | 14 kDa | 0 | 5 |
| YIPF6_HUMAN | YIPF6 | 26 kDa | 3 | 0 |
| NRM_HUMAN | NRM | 29 kDa | 3 | 0 |
| B1AH87_HUMAN (+1) | TSPO | 12 kDa | 0 | 3 |
| F206A_HUMAN | FAM206A | 20 kDa | 3 | 0 |
| IF4A1_HUMAN (+9) | EIF4A1 | 46 kDa | 0 | 3 |
| F6SKB8_HUMAN (+1) | NECAP2 | 22 kDa | 3 | 0 |
| AES_HUMAN | AES | 22 kDa | 3 | 0 |
| GAPR1_HUMAN | GLIPR2 | 17 kDa | 2 | 0 |
| RAB2B_HUMAN | RAB2B | 24 kDa | 16 | 0 |
| CX7A2_HUMAN (+1) | COX7A2 | 9 kDa | 0 | 0 |
| C1D_HUMAN (+1) | C1D | 16 kDa | 0 | 3 |
| IFT22_HUMAN | IFT22 | 21 kDa | 3 | 0 |
| H7C330_HUMAN (+1) | PRKAR2A | 10 kDa | 0 | 0 |
| CNRP1_HUMAN | CNRIP1 | 19 kDa | 2 | 0 |
| G3V445_HUMAN (+1) | OSGEP | 16 kDa | 0 | 0 |
| B2CL2_HUMAN (+2) | BCL2L2 | 21 kDa | 2 | 0 |
| PT117_HUMAN | PET117 | 9 kDa | 0 | 0 |
| DMAC1_HUMAN | DMAC1 | 12 kDa | 0 | 0 |
| CO040_HUMAN | C15orf40 | 16 kDa | 0 | 2 |
| NUCKS_HUMAN (+1) | NUCKS1 | 27 kDa | 0 | 0 |
| H0YC42_HUMAN | | 31 kDa | 10 | 0 |
| RAB8B_HUMAN | RAB8B | 24 kDa | 12 | 0 |
| POMP_HUMAN | POMP | 16 kDa | 0 | 2 |
| B4DR61_HUMAN (+1) | SEC61A1 | 53 kDa | 0 | 2 |
| S18L2_HUMAN | SS18L2 | 9 kDa | 0 | 0 |
| SPT4H_HUMAN | SUPT4H1 | 13 kDa | 0 | 0 |
| A0A0A0MRR7_HUMAN (+1) | SNRPC | 20 kDa | 3 | 0 |
| ARCH_HUMAN | ZBTB8OS | 19 kDa | 3 | 0 |
| ARL5B_HUMAN | ARL5B | 20 kDa | 4 | 0 |
| COA4_HUMAN | COA4 | 10 kDa | 0 | 0 |
| RPC10_HUMAN | POLR3K | 12 kDa | 0 | 0 |
| E7ETM6_HUMAN (+1) | SYS1 | 15 kDa | 0 | 3 |
| J3KSJ5_HUMAN (+3) | C17orf62 | 21 kDa | 3 | 0 |
| J3KRW7_HUMAN (+2) | TMEM199 | 10 kDa | 3 | 0 |
| A0A087X027_HUMAN (+3) | SETSIP | 34 kDa | 3 | 0 |
| C9IZU3_HUMAN (+3) | sept-02 | 15 kDa | 0 | 2 |
| CA109_HUMAN (+1) | C1orf109 | 23 kDa | 3 | 0 |
| GOGA5_HUMAN | GOLGA5 | 83 kDa | 0 | 0 |
| DNAL1_HUMAN (+1) | DNAL1 | 22 kDa | 3 | 0 |
| CKLF6_HUMAN | CMTM6 | 20 kDa | 3 | 0 |
| CHCH7_HUMAN (+5) | CHCHD7 | 10 kDa | 0 | 0 |
| RTL8A_HUMAN | RTL8A | 13 kDa | 0 | 0 |
| TRIR_HUMAN | TRIR | 18 kDa | 0 | 0 |
| SDCB1_HUMAN | SDCBP | 32 kDa | 0 | 0 |
| UB2D1_HUMAN | UBE2D1 | 17 kDa | 0 | 3 |
| PDRG1_HUMAN | PDRG1 | 16 kDa | 0 | 2 |
| D6RDI2_HUMAN (+2) | LUC7L3 | 21 kDa | 0 | 0 |
| A8MUD9_HUMAN (+1) | RPL7 | 24 kDa | 2 | 0 |
| KTHY_HUMAN | DTYMK | 24 kDa | 3 | 0 |
| J3KNQ4_HUMAN (+1) | PARVA | 47 kDa | 0 | 2 |
| SAP3_HUMAN | GM2A | 21 kDa | 2 | 0 |
| T126B_HUMAN | TMEM126B | 26 kDa | 2 | 0 |
| KLHL7_HUMAN | KLHL7 | 66 kDa | 2 | 0 |
| PXMP4_HUMAN | PXMP4 | 24 kDa | 3 | 0 |
| A0A087WX09_HUMAN (+11) | DDX3X | 10 kDa | 0 | 2 |
| OXSR1_HUMAN | OXSR1 | 58 kDa | 0 | 1 |
| BORC5_HUMAN (+1) | BORCS5 | 22 kDa | 1 | 0 |
| A8MXB7_HUMAN (+1) | SNX24 | 24 kDa | 1 | 0 |
| Q8WV07\|LTO1_HUMAN | | ? | 0 | 1 |
| HTD2_HUMAN (+1) | HTD2 | 19 kDa | 0 | 0 |
| PELO_HUMAN | PELO | 43 kDa | 2 | 0 |
| HACD2_HUMAN | HACD2 | 28 kDa | 1 | 0 |
| 4EBP2_HUMAN | EIF4EBP2 | 13 kDa | 0 | 2 |
| COX3_HUMAN | MT-CO3 | 30 kDa | 2 | 0 |
| T10B_HUMAN | TIMM10B | 12 kDa | 0 | 1 |
| ALKB5_HUMAN | ALKBH5 | 44 kDa | 0 | 0 |
| C9J0D1_HUMAN (+2) | H2AFV | 13 kDa | 0 | 12 |
| MGST1_HUMAN | MGST1 | 18 kDa | 0 | 1 |
| MOV10_HUMAN (+1) | MOV10 | 114 kDa | 1 | 0 |
| A0A087X0H9_HUMAN (+1) | RBM26 | 114 kDa | 0 | 1 |
| C56D2_HUMAN (+1) | CYB561D2 | 24 kDa | 3 | 0 |
| CYB5B_HUMAN (+1) | CYB5B | 16 kDa | 11 | 0 |
| TCTA_HUMAN | TCTA | 11 kDa | 0 | 0 |
| E41L2_HUMAN (+6) | EPB41L2 | 113 kDa | 0 | 1 |
| HS90A_HUMAN | HSP90AA1 | 85 kDa | 0 | 3 |
| E7EPB3_HUMAN (+1) | RPL14 | 15 kDa | 0 | 1 |
| NEPR1_HUMAN | CNEP1R1 | 14 kDa | 0 | 1 |
| SDHF3_HUMAN | SDHAF3 | 15 kDa | 0 | 1 |
| A2A2E0_HUMAN (+1) | MANBAL | 6 kDa | 0 | 1 |
| B1AHC9_HUMAN (+1) | XRCC6 | 64 kDa | 1 | 0 |
| HDDC2_HUMAN (+2) | HDDC2 | 23 kDa | 1 | 0 |
| PIP_HUMAN | PIP | 17 kDa | 1 | 0 |
| A0A087WTT1_HUMAN (+3) | PABPC1 | 59 kDa | 1 | 1 |
| A0A0A0MT33_HUMAN (+1) | SCAF8 | 149 kDa | 1 | 1 |
| DNM1L_HUMAN (+1) | DNM1L | 82 kDa | 1 | 1 |
| E7EMN6_HUMAN (+3) | PPP1R2 | 19 kDa | 1 | 1 |
| NEDD1_HUMAN | NEDD1 | 72 kDa | 0 | 0 |
| CA174_HUMAN | C1orf174 | 26 kDa | 1 | 0 |
| DNJC2_HUMAN | DNAJC2 | 72 kDa | 0 | 0 |
| RL29_HUMAN (+1) | RPL29 | 18 kDa | 1 | 1 |
| RRBP1_HUMAN | RRBP1 | 152 kDa | 1 | 1 |
| TYSD1_HUMAN | TYSND1 | 59 kDa | 0 | 0 |
| CALR_HUMAN | CALR | 48 kDa | 0 | 1 |
| SND1_HUMAN | SND1 | 102 kDa | 0 | 1 |
| HDGF_HUMAN | HDGF | 27 kDa | 1 | 1 |
| CKAP2_HUMAN | CKAP2 | 77 kDa | 1 | 0 |
| 1433B_HUMAN | YWHAB | 28 kDa | 1 | 1 |
| HGB1A_HUMAN (+2) | HMGB1P1 | 24 kDa | 1 | 0 |
| SH3G1_HUMAN | SH3GL1 | 41 kDa | 0 | 0 |
| RFA2_HUMAN | RPA2 | 29 kDa | 1 | 0 |
| H3BMW4_HUMAN (+1) | TMEM208 | 16 kDa | 1 | 1 |
| A0A096LP69_HUMAN (+1) | CD99 | 19 kDa | 1 | 1 |
| BCAS3_HUMAN | BCAS3 | 101 kDa | 0 | 1 |
| UT14A_HUMAN (+2) | UTP14A | 88 kDa | 0 | 1 |
| CWC27_HUMAN (+1) | CWC27 | 54 kDa | 0 | 1 |
| E7EQL1_HUMAN (+1) | | 15 kDa | 1 | 1 |
| CSRP2_HUMAN (+2) | CSRP2 | 21 kDa | 1 | 0 |
| H3BM38_HUMAN (+1) | SNAP23 | 14 kDa | 0 | 1 |
| ATPF1_HUMAN (+1) | ATPAF1 | 36 kDa | 1 | 0 |
| ETFB_HUMAN | ETFB | 28 kDa | 0 | 1 |
| H3BLW9_HUMAN | SWI5 | 17 kDa | 0 | 1 |
| A0A024R4E5_HUMAN (+14) | HDLBP | 141 kDa | 0 | 0 |
| A0A075B7C0_HUMAN (+5) | CAPNS1 | 22 kDa | 2 | 0 |
| A0A087WW59_HUMAN (+1) | C11orf96 | 14 kDa | 0 | 2 |
| AFAD_HUMAN (+2) | AFDN | 207 kDa | 0 | 2 |
| B4DWR3_HUMAN (+1) | VBP1 | 22 kDa | 2 | 0 |
| B7WNH4_HUMAN (+3) | SMIM7 | 12 kDa | 0 | 0 |
| B7ZBC8_HUMAN (+2) | KATNA1 | 32 kDa | 2 | 0 |
| CA122_HUMAN | C1orf122 | 11 kDa | 0 | 0 |
| COMDA_HUMAN (+1) | COMMD10 | 23 kDa | 2 | 0 |
| GLRX3_HUMAN | GLRX3 | 37 kDa | 0 | 2 |
| HSBP1_HUMAN | HSBP1 | 9 kDa | 0 | 0 |
| J3QL97_HUMAN (+1) | ZSWIM7 | 8 kDa | 0 | 2 |
| MCFD2_HUMAN | MCFD2 | 16 kDa | 0 | 2 |
| NCALD_HUMAN | NCALD | 22 kDa | 6 | 0 |
| Q9BUV8\|RAB5I_HUMAN | | ? | 0 | 0 |
| RAGP1_HUMAN | RANGAP1 | 64 kDa | 0 | 2 |
| RAP2A_HUMAN | RAP2A | 21 kDa | 6 | 0 |
| RASK_HUMAN | KRAS | 22 kDa | 6 | 0 |
| SIM13_HUMAN | SMIM13 | 10 kDa | 0 | 0 |
| TIM22_HUMAN | TIMM22 | 20 kDa | 2 | 0 |
| TIM8A_HUMAN | TIMM8A | 11 kDa | 0 | 0 |
| TPPP3_HUMAN | TPPP3 | 19 kDa | 2 | 0 |
| MINT_HUMAN | SPEN | 402 kDa | 0 | 2 |
| A0A087WWY3_HUMAN (+2) | FLNA | 246 kDa | 0 | 0 |
| G3V3U6_HUMAN (+1) | MRPL52 | 6 kDa | 0 | 0 |
| K7EJE8_HUMAN (+1) | LONP1 | 93 kDa | 2 | 0 |
| PP4R2_HUMAN | PPP4R2 | 47 kDa | 2 | 0 |
| PARP1_HUMAN | PARP1 | 113 kDa | 0 | 2 |
| JOS2_HUMAN (+1) | JOSD2 | 21 kDa | 2 | 0 |
| H10_HUMAN | H1F0 | 21 kDa | 2 | 0 |
| MCUR1_HUMAN | MCUR1 | 40 kDa | 2 | 0 |
| PPIC_HUMAN | PPIC | 23 kDa | 2 | 0 |
| PGRC1_HUMAN | PGRMC1 | 22 kDa | 2 | 0 |
| I3L1L3_HUMAN (+2) | MYBBP1A | 140 kDa | 0 | 0 |
| PAWR_HUMAN | PAWR | 37 kDa | 0 | 2 |
| RB39B_HUMAN | RAB39B | 25 kDa | 6 | 0 |
| NDUF8_HUMAN | NDUFAF8 | 8 kDa | 0 | 0 |
| KTBL1_HUMAN | KATNBL1 | 35 kDa | 2 | 0 |
| MCEE_HUMAN | MCEE | 19 kDa | 2 | 0 |
| NMES1_HUMAN | NMES1 | 10 kDa | 0 | 0 |
| MED28_HUMAN | MED28 | 20 kDa | 2 | 0 |
| SRRM2_HUMAN | SRRM2 | 300 kDa | 2 | 0 |
| RPAB5_HUMAN | POLR2L | 8 kDa | 0 | 0 |
| DHX9_HUMAN | DHX9 | 141 kDa | 0 | 2 |
| HLPDA_HUMAN | HILPDA | 7 kDa | 0 | 0 |
| REEP6_HUMAN | REEP6 | 23 kDa | 2 | 0 |
| SYFB_HUMAN | FARSB | 66 kDa | 2 | 0 |
| A0A024QZX5_HUMAN (+3) | SERPINB6 | 43 kDa | 0 | 2 |
| G3V448_HUMAN (+1) | TMX1 | 12 kDa | 2 | 0 |
| LHPL2_HUMAN | LHFPL2 | 24 kDa | 2 | 0 |
| GRN_HUMAN (+1) | GRN | 64 kDa | 0 | 0 |
| A0A0C4DG89_HUMAN (+1) | DDX46 | 117 kDa | 0 | 0 |
| WDR13_HUMAN | WDR13 | 54 kDa | 0 | 2 |
| RHOB_HUMAN | RHOB | 22 kDa | 10 | 0 |
| LMO4_HUMAN | LMO4 | 18 kDa | 2 | 0 |
| MZT2A_HUMAN | MZT2A | 16 kDa | 8 | 0 |
| DSRAD_HUMAN (+4) | ADAR | 136 kDa | 0 | 0 |
| F8WDH1_HUMAN (+1) | TMEM50B | 17 kDa | 0 | 0 |
| PURA2_HUMAN | ADSS | 50 kDa | 2 | 0 |
| CDO1_HUMAN | CDO1 | 23 kDa | 2 | 0 |
| FRIL_HUMAN | FTL | 20 kDa | 2 | 0 |
| MED30_HUMAN | MED30 | 20 kDa | 2 | 0 |
| SPART_HUMAN | SPART | 73 kDa | 0 | 2 |
| sp\|P62987\|RL40_HUMAN Ubiquitin-60S ribosomal protein L40 OS=Homo sapiens GN=UBA52 PE=1 SV=2 | | 15 kDa | 0 | 0 |
| IMP3_HUMAN | IMP3 | 22 kDa | 2 | 0 |
| B0YIW6_HUMAN (+2) | ARCN1 | 62 kDa | 0 | 2 |
| K7ELX5_HUMAN (+1) | TLCD1 | 17 kDa | 2 | 0 |
| CSMT1_HUMAN | CCSMST1 | 15 kDa | 0 | 2 |
| A0A0U1RQM8_HUMAN (+4) | QARS | 19 kDa | 2 | 0 |
| MPLKI_HUMAN | MPLKIP | 19 kDa | 2 | 0 |
| I3L2L5_HUMAN (+3) | MCRIP1 | 10 kDa | 0 | 0 |
| RTRAF_HUMAN | RTRAF | 28 kDa | 0 | 2 |
| ANR39_HUMAN | ANKRD39 | 20 kDa | 1 | 0 |
| E9PIK4_HUMAN (+10) | TP53I11 | 14 kDa | 0 | 1 |
| RIPK1_HUMAN | RIPK1 | 76 kDa | 0 | 2 |
| TIGAR_HUMAN | TIGAR | 30 kDa | 1 | 0 |
| CYTM1_HUMAN | CYSTM1 | 11 kDa | 0 | 1 |
| TOM5_HUMAN | TOMM5 | 6 kDa | 0 | 0 |
| A0A087WWF1_HUMAN (+1) | SELENOM | 16 kDa | 0 | 1 |
| E9PE46_HUMAN | ZNF738 | 14 kDa | 0 | 0 |
| ATOX1_HUMAN (+2) | ATOX1 | 7 kDa | 0 | 0 |
| A0A087X1Z3_HUMAN (+2) | PSME2 | 29 kDa | 0 | 2 |
| EMC9_HUMAN (+1) | EMC9 | 23 kDa | 1 | 0 |
| RENT1_HUMAN | UPF1 | 124 kDa | 1 | 0 |
| A0A0A0MRX9_HUMAN (+2) | PITRM1 | 67 kDa | 0 | 1 |
| Q5JR08_HUMAN (+1) | RHOC | 22 kDa | 27 | 0 |
| HNRPF_HUMAN | HNRNPF | 46 kDa | 1 | 0 |
| F8WBE5_HUMAN (+1) | TFRC | 9 kDa | 0 | 0 |
| A0A1B0GUC2_HUMAN (+2) | UMAD1 | 9 kDa | 1 | 0 |
| PP4C_HUMAN | PPP4C | 35 kDa | 0 | 0 |
| C9IZ93_HUMAN (+4) | UBE2F | 19 kDa | 1 | 0 |
| IFT20_HUMAN | IFT20 | 15 kDa | 0 | 1 |
| H0Y449_HUMAN | YBX1 | 42 kDa | 1 | 0 |
| E9PIE4_HUMAN (+1) | MTCH2 | 29 kDa | 0 | 0 |
| TACO1_HUMAN | TACO1 | 32 kDa | 1 | 0 |
| C1QBP_HUMAN (+2) | C1QBP | 31 kDa | 0 | 1 |
| GLRX2_HUMAN | GLRX2 | 18 kDa | 0 | 1 |
| A0A0A0MT77_HUMAN (+2) | DNAJC24 | 17 kDa | 1 | 0 |
| C9JW96_HUMAN (+4) | PHB | 27 kDa | 1 | 0 |
| CKLF8_HUMAN | CMTM8 | 20 kDa | 0 | 1 |
| BCCIP_HUMAN | BCCIP | 36 kDa | 0 | 0 |
| EDC4_HUMAN | EDC4 | 152 kDa | 0 | 0 |
| DHX15_HUMAN | DHX15 | 91 kDa | 0 | 0 |
| B2L11_HUMAN | BCL2L11 | 22 kDa | 0 | 0 |
| AP1G1_HUMAN (+1) | AP1G1 | 91 kDa | 0 | 2 |
| B1AKV2_HUMAN (+3) | UQCC1 | 25 kDa | 1 | 0 |
| CDV3_HUMAN (+1) | CDV3 | 27 kDa | 0 | 0 |
| D6R9W4_HUMAN (+2) | DBN1 | 36 kDa | 0 | 1 |
| M0QXU7_HUMAN (+2) | TIMM44 | 31 kDa | 0 | 0 |
| E7EQY4_HUMAN (+2) | MTA3 | 59 kDa | 0 | 0 |
| AP3M1_HUMAN | AP3M1 | 47 kDa | 0 | 1 |
| HPRT_HUMAN | HPRT1 | 25 kDa | 0 | 0 |
| AIG1_HUMAN (+3) | AIG1 | 28 kDa | 1 | 0 |
| SMIM8_HUMAN | SMIM8 | 11 kDa | 0 | 0 |
| RT31_HUMAN | MRPS31 | 45 kDa | 1 | 0 |
| A0A0A0MRV5_HUMAN (+5) | ZNF573 | 17 kDa | 0 | 0 |
| A0A0D9SF70_HUMAN (+2) | ARFGAP2 | 42 kDa | 0 | 0 |
| H3BUC8\|H3BUC8_HUMAN-DECOY (+1) | | ? | 0 | 1 |
| KISHB_HUMAN | TMEM167B | 8 kDa | 0 | 0 |
| A0A087WT42_HUMAN (+2) | SLC2A8 | 34 kDa | 0 | 0 |
| APOC3_HUMAN (+1) | APOC3 | 11 kDa | 0 | 0 |
| PREY_HUMAN | PYURF | 13 kDa | 0 | 0 |
| SIM20_HUMAN | SMIM20 | 8 kDa | 0 | 0 |
| A0A0B4J1T5\|A0A0B4J1T5_HUMAN-DECOY (+1) | | ? | 1 | 0 |
| GABPA_HUMAN | GABPA | 51 kDa | 0 | 1 |
| CFDP1_HUMAN | CFDP1 | 34 kDa | 0 | 0 |
| P11021\|BIP_HUMAN-DECOY | | ? | 0 | 1 |
| CRKL_HUMAN | CRKL | 34 kDa | 0 | 0 |
| MAGD2_HUMAN | MAGED2 | 65 kDa | 0 | 0 |
| I3L0U0_HUMAN | WSCD1 | 15 kDa | 0 | 0 |
| GBRAP_HUMAN (+1) | GABARAP | 14 kDa | 0 | 4 |
| K7ENW4_HUMAN (+1) | NACC1 | 18 kDa | 0 | 2 |
| H0YB73_HUMAN (+3) | UBE2W | 18 kDa | 0 | 1 |
| ABCF3_HUMAN | ABCF3 | 80 kDa | 0 | 0 |
| BORC8_HUMAN | BORCS8 | 13 kDa | 0 | 1 |
| H3BS71_HUMAN (+3) | SENP8 | 8 kDa | 2 | 0 |
| J3QRS3_HUMAN (+1) | MYL12A | 20 kDa | 32 | 0 |
| sp\|P01112\|RASH_HUMAN GTPase HRas OS=Homo sapiens GN=HRAS PE=1 SV=1 (+1) | | 21 kDa | 8 | 0 |
| RTCB_HUMAN | RTCB | 55 kDa | 0 | 0 |
| AKIP_HUMAN | AURKAIP1 | 22 kDa | 0 | 2 |
| UBR4_HUMAN | UBR4 | 574 kDa | 0 | 0 |
| CKLF4_HUMAN (+1) | CMTM4 | 26 kDa | 2 | 0 |
| J3KQ69_HUMAN (+1) | MCM3 | 92 kDa | 0 | 1 |
| A0A087WX02_HUMAN (+2) | SELENOW | 9 kDa | 0 | 0 |
| A8MU39_HUMAN (+1) | PPP5C | 42 kDa | 0 | 0 |
| APR_HUMAN | PMAIP1 | 6 kDa | 0 | 0 |
| B1AN99_HUMAN (+1) | PRSS3 | 19 kDa | 0 | 0 |
| B4DFL6_HUMAN (+3) | TMEM14B | 15 kDa | 0 | 0 |
| BYST_HUMAN (+1) | BYSL | 50 kDa | 0 | 0 |
| C42S1_HUMAN | CDC42SE1 | 9 kDa | 0 | 0 |
| CD048_HUMAN | C4orf48 | 10 kDa | 0 | 0 |
| CMC4_HUMAN | CMC4 | 8 kDa | 0 | 0 |
| E5RHF4_HUMAN (+1) | MRPL15 | 22 kDa | 0 | 0 |
| E5RID5_HUMAN (+3) | CA2 | 11 kDa | 0 | 0 |
| ENL_HUMAN | MLLT1 | 62 kDa | 0 | 0 |
| F8WAZ6_HUMAN (+4) | PMPCB | 22 kDa | 0 | 0 |
| GBG10_HUMAN | GNG10 | 7 kDa | 0 | 0 |
| H0Y679_HUMAN (+2) | PRCC | 29 kDa | 0 | 0 |
| H3BM93_HUMAN (+4) | NUP93 | 20 kDa | 0 | 0 |
| I2BPL_HUMAN | IRF2BPL | 83 kDa | 0 | 0 |
| KCD15_HUMAN | KCTD15 | 32 kDa | 0 | 0 |
| LXN_HUMAN | LXN | 26 kDa | 0 | 0 |
| NSF1C_HUMAN | NSFL1C | 41 kDa | 0 | 0 |
| TAB2_HUMAN (+1) | TAB2 | 76 kDa | 0 | 0 |
| ZA2G_HUMAN | AZGP1 | 34 kDa | 0 | 0 |
| TBB2B_HUMAN | TUBB2B | 50 kDa | 0 | 0 |
| A0A087X1C1_HUMAN (+3) | TMSB15B | 9 kDa | 0 | 0 |
| F6T1Q0_HUMAN (+1) | PDE12 | 52 kDa | 0 | 0 |
| A0A087WZM8_HUMAN (+1) | TMEM254 | 11 kDa | 0 | 0 |
| A0A0C4DGB0_HUMAN (+1) | SCOC | 9 kDa | 0 | 0 |
| A0A0G2JJT9_HUMAN (+3) | VARS | 18 kDa | 0 | 0 |
| B2WTI3_HUMAN (+3) | JMJD6 | 39 kDa | 0 | 0 |
| B7Z982_HUMAN (+2) | PLRG1 | 15 kDa | 0 | 0 |
| CA052_HUMAN | C1orf52 | 21 kDa | 0 | 0 |
| CH033_HUMAN (+4) | C8orf33 | 25 kDa | 0 | 0 |
| CNIH1_HUMAN (+3) | CNIH1 | 17 kDa | 0 | 0 |
| E9PMG1_HUMAN (+3) | REPS1 | 80 kDa | 0 | 0 |
| F241B_HUMAN | FAM241B | 13 kDa | 0 | 0 |
| G3V2X0_HUMAN (+3) | SUPT16H | 10 kDa | 0 | 0 |
| NU6M_HUMAN | MT-ND6 | 19 kDa | 0 | 0 |
| P66A_HUMAN | GATAD2A | 68 kDa | 0 | 0 |
| RSBNL_HUMAN | RSBN1L | 95 kDa | 0 | 0 |
| TEN1L_HUMAN | TEN1 | 14 kDa | 0 | 0 |
| sp\|B2MG_HUMAN\| (+1) | | 14 kDa | 0 | 0 |
| Q5T8D1_HUMAN (+3) | YME1L1 | 28 kDa | 0 | 0 |
| Ab_H10_light-chain (+8) | | 26 kDa | 0 | 0 |
| A0A087WTZ5_HUMAN (+3) | UBXN1 | 27 kDa | 0 | 1 |
| A0A0A0MTE2_HUMAN (+4) | LMO7 | 158 kDa | 0 | 1 |
| A0A0B4J202_HUMAN (+2) | NMU | 18 kDa | 0 | 1 |
| A0A0J9YYC8_HUMAN (+8) | PRSS2 | 26 kDa | 0 | 1 |
| A8MU27_HUMAN (+2) | SUMO3 | 17 kDa | 0 | 4 |
| B4DHE8_HUMAN (+2) | MSI2 | 35 kDa | 0 | 1 |
| BBC3_HUMAN | BBC3 | 21 kDa | 0 | 1 |
| CLPX_HUMAN (+2) | CLPX | 69 kDa | 0 | 1 |
| DHB4_HUMAN (+6) | HSD17B4 | 80 kDa | 0 | 1 |
| DPOA2_HUMAN (+2) | POLA2 | 66 kDa | 0 | 1 |
| E9PLN8_HUMAN (+3) | | 18 kDa | 0 | 1 |
| E9PN77_HUMAN | CYHR1 | 14 kDa | 0 | 1 |
| H3BP20_HUMAN (+2) | HEXA | 62 kDa | 0 | 1 |
| H3BRG4_HUMAN (+1) | UQCRC2 | 45 kDa | 0 | 1 |
| H7C488_HUMAN (+1) | LZTFL1 | 27 kDa | 0 | 1 |
| ID2_HUMAN | ID2 | 15 kDa | 0 | 1 |
| J3QLR8_HUMAN (+1) | MRPS23 | 18 kDa | 0 | 1 |
| K7EIY4_HUMAN (+7) | STX10 | 26 kDa | 0 | 1 |
| LTV1_HUMAN | LTV1 | 55 kDa | 0 | 1 |
| MPRI_HUMAN | IGF2R | 274 kDa | 0 | 1 |
| MRUPP_HUMAN | | 13 kDa | 0 | 1 |
| ORML2_HUMAN | ORMDL2 | 17 kDa | 0 | 4 |
| Q86XC5_HUMAN | TMEM97 | 8 kDa | 0 | 4 |
| TI17A_HUMAN | TIMM17A | 18 kDa | 0 | 1 |
| sp\|LYSC_CHICK\| | | 16 kDa | 0 | 1 |
| SRC8_HUMAN | CTTN | 62 kDa | 0 | 1 |
| A9J4F5_HUMAN (+1) | ZNF593 | 17 kDa | 0 | 1 |
| B3KNJ4_HUMAN (+2) | SAE1 | 33 kDa | 0 | 0 |
| UBL3_HUMAN | UBL3 | 13 kDa | 0 | 0 |
| A0A0A0MSQ9_HUMAN (+1) | MON1A | 63 kDa | 0 | 1 |
| K7EPN4\|K7EPN4_HUMAN-DECOY (+1) | | ? | 0 | 1 |
| FLNC_HUMAN | FLNC | 291 kDa | 0 | 0 |
| DD19A_HUMAN (+7) | DDX19A | 54 kDa | 0 | 1 |
| CCD43_HUMAN (+1) | CCDC43 | 25 kDa | 0 | 1 |
| UBTD2_HUMAN | UBTD2 | 26 kDa | 0 | 1 |
| EFTU_HUMAN (+1) | TUFM | 50 kDa | 0 | 0 |
| 1433G_HUMAN | YWHAG | 28 kDa | 0 | 1 |
| DDX20_HUMAN (+1) | DDX20 | 92 kDa | 0 | 0 |
| ID3_HUMAN | ID3 | 13 kDa | 0 | 0 |
| TOM40_HUMAN | TOMM40 | 38 kDa | 0 | 1 |
| TM14A_HUMAN | TMEM14A | 11 kDa | 0 | 0 |
| XRN2_HUMAN | XRN2 | 109 kDa | 0 | 1 |
| SERC1_HUMAN | SERINC1 | 50 kDa | 0 | 1 |
| SNX18_HUMAN | SNX18 | 69 kDa | 0 | 0 |
| EGLN1_HUMAN | EGLN1 | 46 kDa | 0 | 1 |
| B8ZZY7_HUMAN (+5) | SERF2 | 9 kDa | 0 | 0 |
| CRK_HUMAN | CRK | 34 kDa | 0 | 0 |
| H0Y4T2_HUMAN (+2) | SLC19A1 | 29 kDa | 0 | 1 |
| Q86Y22\|CONA1_HUMAN-DECOY | | ? | 0 | 0 |
| R4GN98_HUMAN (+1) | S100A6 | 10 kDa | 0 | 0 |
| A0A087WUF6_HUMAN (+2) | FGF2 | 31 kDa | 1 | 0 |
| A0A087WV25_HUMAN (+1) | FGFR1OP | 38 kDa | 1 | 0 |
| A0A096LP22_HUMAN (+3) | PTRH1 | 19 kDa | 1 | 0 |
| A0A0A0MT00_HUMAN (+5) | NFYC | 20 kDa | 1 | 0 |
| A0A0D9SEI0_HUMAN (+1) | C6orf203 | 28 kDa | 1 | 0 |
| A0A0D9SFB0_HUMAN (+12) | FHL1 | 17 kDa | 1 | 0 |
| A0A0J9YWB6_HUMAN (+1) | CTDP1 | 87 kDa | 1 | 0 |
| A0A0J9YWG3_HUMAN (+3) | CNTNAP2 | 36 kDa | 1 | 0 |
| A0A140T9G8_HUMAN (+4) | C6orf136 | 48 kDa | 1 | 0 |
| A0A1B0GUB2_HUMAN (+4) | CDC73 | 51 kDa | 1 | 0 |
| A0A1B0GUK8_HUMAN (+6) | RBMXL1 | 17 kDa | 1 | 0 |
| A0A1W2PQ51_HUMAN (+2) | DDX17 | 80 kDa | 1 | 0 |
| A0A1W2PR36_HUMAN (+1) | GAMT | 24 kDa | 1 | 0 |
| A8MXU7_HUMAN (+2) | BAD | 17 kDa | 1 | 0 |
| AN32E_HUMAN (+2) | ANP32E | 31 kDa | 1 | 0 |
| B5MCW2_HUMAN (+2) | RPL3 | 31 kDa | 1 | 0 |
| B9D2_HUMAN | B9D2 | 19 kDa | 1 | 0 |
| C9J6L4_HUMAN (+3) | CPVL | 11 kDa | 1 | 0 |
| C9J6P4_HUMAN (+1) | ZC3HAV1 | 114 kDa | 1 | 0 |
| C9JB30_HUMAN (+1) | MAPRE3 | 25 kDa | 2 | 0 |
| C9JFM7_HUMAN (+1) | RAB43 | 9 kDa | 5 | 0 |
| CATO_HUMAN | CTSO | 36 kDa | 1 | 0 |
| CI040_HUMAN | C9orf40 | 21 kDa | 1 | 0 |
| CRLS1_HUMAN (+1) | CRLS1 | 33 kDa | 1 | 0 |
| D3YTI2_HUMAN | ACP1 | 9 kDa | 2 | 0 |
| D6RAC7_HUMAN (+3) | G3BP2 | 16 kDa | 1 | 0 |
| DEFM_HUMAN | PDF | 27 kDa | 1 | 0 |
| DNJC7_HUMAN (+10) | DNAJC7 | 56 kDa | 1 | 0 |
| DRAM2_HUMAN | DRAM2 | 30 kDa | 1 | 0 |
| DTD2_HUMAN (+1) | DTD2 | 19 kDa | 1 | 0 |
| E7ERF2_HUMAN (+3) | TCP1 | 47 kDa | 1 | 0 |
| E7EX17_HUMAN (+1) | EIF4B | 70 kDa | 1 | 0 |
| E9PDW2_HUMAN (+1) | OXCT1 | 37 kDa | 1 | 0 |
| EF1B_HUMAN | EEF1B2 | 25 kDa | 2 | 0 |
| ERF3B_HUMAN | GSPT2 | 69 kDa | 1 | 0 |
| ESYT1_HUMAN | ESYT1 | 123 kDa | 1 | 0 |
| F2Z2W6_HUMAN (+3) | HMGN1 | 5 kDa | 1 | 0 |
| F5GZG1_HUMAN | RAP1B | 14 kDa | 10 | 0 |
| F8VR77_HUMAN (+3) | PA2G4 | 31 kDa | 1 | 0 |
| H0Y548_HUMAN | PPIF | 18 kDa | 7 | 0 |
| H0YCR7_HUMAN (+1) | RNH1 | 30 kDa | 1 | 0 |
| H0YHF1_HUMAN | COQ10A | 18 kDa | 1 | 0 |
| H32_HUMAN | HIST2H3A | 15 kDa | 5 | 0 |
| H7BY84_HUMAN (+2) | GTF3C5 | 40 kDa | 1 | 0 |
| H7C068_HUMAN | ATP5O | 10 kDa | 10 | 0 |
| ID4_HUMAN | ID4 | 17 kDa | 1 | 0 |
| J3KNC0_HUMAN (+1) | GTF2A1 | 7 kDa | 1 | 0 |
| J3KS31_HUMAN (+3) | ZNF207 | 35 kDa | 1 | 0 |
| KBRS1_HUMAN | NKIRAS1 | 22 kDa | 1 | 0 |
| MMAB_HUMAN (+1) | MMAB | 27 kDa | 1 | 0 |
| MRP_HUMAN | MARCKSL1 | 20 kDa | 1 | 0 |
| NDKB_HUMAN | NME2 | 17 kDa | 76 | 0 |
| NUDT3_HUMAN | NUDT3 | 19 kDa | 7 | 0 |
| NUDT4_HUMAN (+1) | NUDT4 | 20 kDa | 3 | 0 |
| Q5T760_HUMAN (+1) | SRSF11 | 42 kDa | 1 | 0 |
| RAB5A_HUMAN | RAB5A | 24 kDa | 2 | 0 |
| RHOQ_HUMAN | RHOQ | 23 kDa | 2 | 0 |
| RING2_HUMAN | RNF2 | 38 kDa | 1 | 0 |
| RNF11_HUMAN | RNF11 | 17 kDa | 1 | 0 |
| SCAM4_HUMAN | SCAMP4 | 26 kDa | 1 | 0 |
| TIMP3_HUMAN | TIMP3 | 24 kDa | 1 | 0 |
| UFSP1_HUMAN | UFSP1 | 15 kDa | 1 | 0 |
| YBOX3_HUMAN | YBX3 | 40 kDa | 1 | 0 |
| ZNHI1_HUMAN | ZNHIT1 | 18 kDa | 1 | 0 |
| Q5VX52\|SPAT1_HUMAN-DECOY | | ? | 0 | 0 |
| B4DEH8_HUMAN (+3) | PABPN1 | 19 kDa | 1 | 0 |
| G3V579_HUMAN (+2) | PNN | 14 kDa | 0 | 0 |
| COX7C_HUMAN (+1) | COX7C | 7 kDa | 0 | 0 |
| A0A087WWP5_HUMAN (+1) | TAF10 | 12 kDa | 0 | 0 |
| D3YTC7_HUMAN (+1) | PEMT | 23 kDa | 0 | 1 |
| RGS16_HUMAN | RGS16 | 23 kDa | 1 | 0 |
| A0A0A0MQS5_HUMAN (+1) | WDR83OS | 12 kDa | 0 | 0 |
| TX261_HUMAN | TEX261 | 23 kDa | 0 | 1 |
| FILA2_HUMAN (+1) | FLG2 | 248 kDa | 0 | 1 |
| B4E3S0_HUMAN (+2) | CORO1C | 42 kDa | 0 | 0 |
| A0A087WV97_HUMAN (+2) | SMAP2 | 46 kDa | 1 | 0 |
| PHLA3_HUMAN | PHLDA3 | 14 kDa | 0 | 0 |
| H7BZ35_HUMAN (+1) | DARS | 22 kDa | 0 | 0 |
| B4DZ60_HUMAN (+2) | AMD1 | 24 kDa | 0 | 0 |
| E7ERS3_HUMAN (+2) | ZC3H18 | 109 kDa | 0 | 0 |
| ID=ARBNEW_243 Name=g6568_1 Supercontig_1.34 | | 11 kDa | 0 | 0 |
| A9UJP8_HUMAN (+2) | TTC38 | 29 kDa | 0 | 1 |
| AN13B_HUMAN | ANKRD13B | 70 kDa | 1 | 0 |
| G8JLA2_HUMAN | MYL6 | 17 kDa | 0 | 40 |
| T179B_HUMAN | TMEM179B | 24 kDa | 0 | 0 |
| CQ100_HUMAN | C17orf100 | 18 kDa | 0 | 1 |
| F5H8D7\|F5H8D7_HUMAN-DECOY (+2) | | ? | 0 | 1 |
| K7ENG2_HUMAN (+1) | U2AF2 | 34 kDa | 0 | 1 |
| J3QSZ6_HUMAN (+1) | TMEM138 | 13 kDa | 0 | 1 |
| MORN4_HUMAN | MORN4 | 16 kDa | 0 | 1 |
| A0A3B3ITY0\|A0A3B3ITY0_HUMAN | | ? | 1 | 0 |
| sp\|K1C15_SHEEP\| | | 49 kDa | 0 | 3 |
| DCA16_HUMAN | DCAF16 | 24 kDa | 1 | 0 |
| A0A075B7G4_HUMAN (+15) | ZNF595 | 71 kDa | 0 | 0 |
| VDAC3_HUMAN | VDAC3 | 31 kDa | 0 | 0 |
| A0A0A0MSC4_HUMAN (+2) | PDGFA | 10 kDa | 0 | 0 |
| F8WAJ0_HUMAN | DDX31 | 84 kDa | 0 | 0 |
| B4DT96_HUMAN (+2) | NUPL2 | 17 kDa | 1 | 0 |
