# Supplementary material for: Loss of Ecrg4 improves calcium oxalate nephropathy
Source: PLoS One. 2022 Oct 13;17(10):e0275972. doi: 10.1371/journal.pone.0275972 (PMC9560046; doi:10.1371/journal.pone.0275972)
Supplement: S1 File — (DOCX) [file pone.0275972.s018.docx]

# Supplemental methods

**HEK 293 cell transfection:** The HEK 293 cell culture was carried out using standard procedures. Cells were transfected with plasmid containing the subunit cDNA (#MR201031, OriGene Technologies, USA) using FuGENE® HD Transfection Reagent (#E2311, Promega Wisconsin, USA).

For protein extraction, the same protocol as before was used.

The quantification mix was done in NuPAGE LDS sample Buffer and 20ug (tissues)/30ug (cells) were loaded on the gel. MES running buffer was used to run the gel, followed by staining O/N with Candianno Coomassie, de-staining, gel scanning and bands sectioning.

**Database searching:** Tandem mass spectra were extracted. All samples were analyzed using Mascot (matrix Science, London, UK, version 2.6.2). Mascot was searched with a fragment ion mass tolerance of 0.020Da and a parent ion tolerance of 10.0 PPM. Carbamidomethyl of cysteine was specified in Mascot as a fixed modification. Oxidation of methionine and N-acetylation of the protein N-terminus were specified in Mascot as variable modifications.

**Criteria for protein identification:** Identified proteins were re-evaluated using Scaffold files (Proteome software, Portland, OR), which combine the Peptide Prophet (1) and Protein Prophet algorithms (2). A threshold of 90% with at least one peptide was set as an acceptance criterion for protein identification.

1. Keller A, Purvine S, Nesvizhskii AI, Stolyar S, Goodlett DR, Kolker E. Experimental Protein Mixture for Validating Tandem Mass Spectral Analysis. OMICS: A Journal of Integrative Biology. 2002;6(2):207-12.

2. Nesvizhskii AI, Keller A, Kolker E, Aebersold R. A Statistical Model for Identifying Proteins by Tandem Mass Spectrometry. Analytical Chemistry. 2003;75(17):4646-58.
