# Supplementary material for: Loss of Ecrg4 improves calcium oxalate nephropathy
Source: PLoS One. 2022 Oct 13;17(10):e0275972. doi: 10.1371/journal.pone.0275972 (PMC9560046; doi:10.1371/journal.pone.0275972)

Kidney (30g protein)

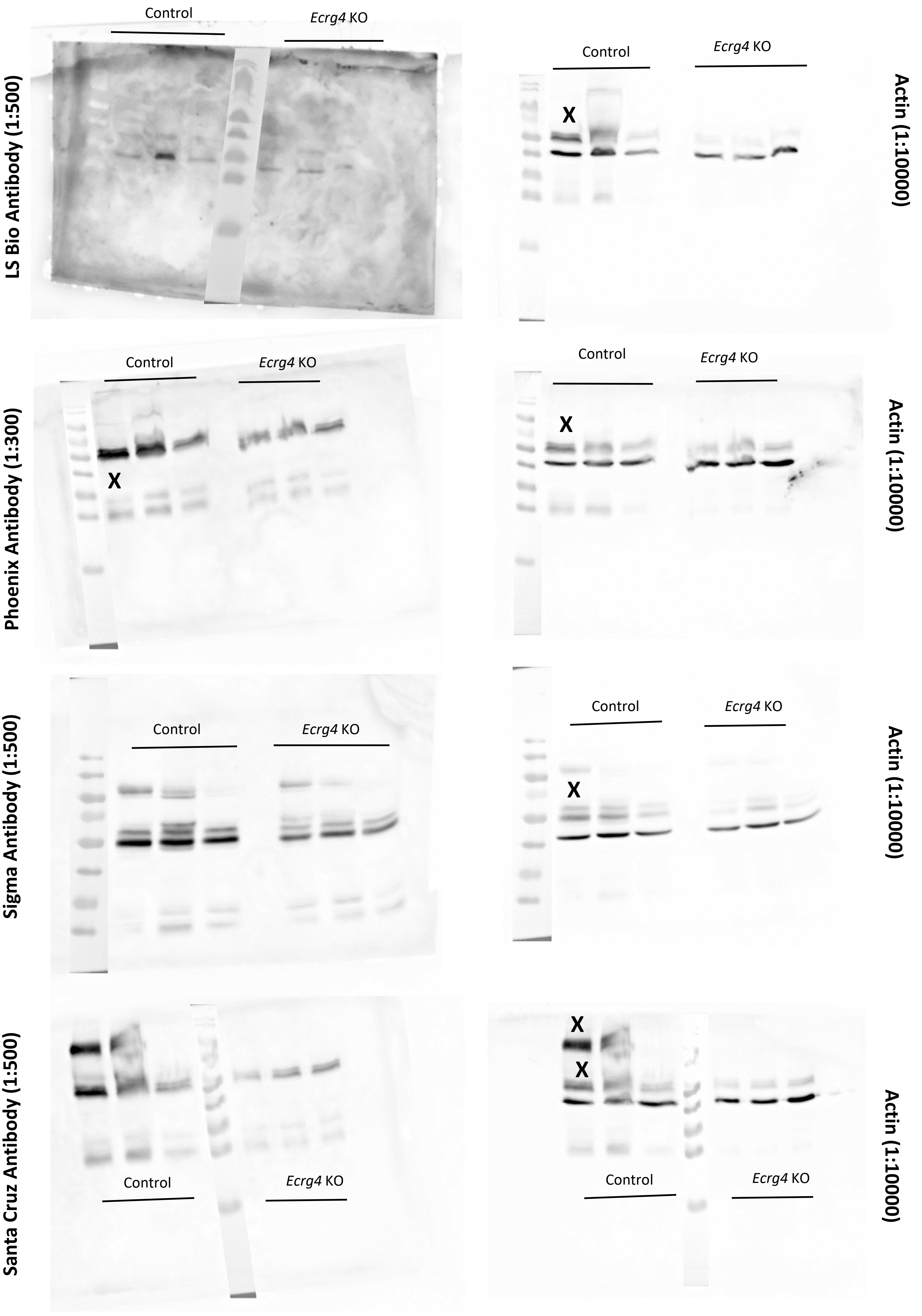

Supplement: S2 Raw images — (PDF) [file pone.0275972.s020.pdf]
