# Supplementary material for: Loss of Ecrg4 improves calcium oxalate nephropathy
Source: PLoS One. 2022 Oct 13;17(10):e0275972. doi: 10.1371/journal.pone.0275972 (PMC9560046; doi:10.1371/journal.pone.0275972)
Supplement: Attachment — Submitted filename: REVIEW_JX.docx [file pone.0275972.s023.docx]

This study characterized the expression of Ecrg4 mRNA in different tissues as well as the expression pattern along the renal segment. The manuscript is very well written. The experimental approach is straightforward and clearly described. By in vivo and in vitro studies, authors reported the potential role of Ecrg4 in calcium oxalate nephropathy. Although there are several interesting concepts put forward by the data, there remains several unanswered questions and the underlying mechanisms are elusive. For this reason, I have some suggestions to improve the manuscript:

Major:

1. In response to hypercalciuria, Ecrg4 mRNA is upregulated, however this is not seen in furosemide-induced calciuria. In addition, renal Ecrg4 mRNA is not changed by PTH or 1,25(OH)3-VD. Therefore, it is speculated that beside directly sensing urine Ca concentration, renal Ecrg4 mRNA can also respond to other unknown local or systemic factors. Discussion about the underlying mechanisms for different hypercalciuric models and the resulting possible systemic changes could help to explain the discrepancy in renal Ecrg4 mRNA response to various hypercalciuric models. In addition, have author measured the Ecrg4 mRNA expression in control mice on calcium-oxalate diet, which induces hypercalciuria.
2. Is there sex difference in Ecrg4 mRNA expression, given that female mice are resistant to crystal formation in all conditions?
3. Plasma Ca concentration is comparable between the genotypes on calcium-oxalate diet, Ecrg4 KO shows lower urinary Ca excretion, which might indicate higher renal Ca reabsorption, meanwhile, mice might have lower intestinal Ca absorption to maintain the plasma Ca homeostasis. The authors measured mRNA expression of genes related to renal calcium handling, and found no differences between genotypes. mRNA expression is not always consistent with the protein abundance, therefore, WB to check the protein levels should provide more precise information. In addition, have the authors checked the hormone levels related to Ca homeostasis, PTH, VD3, FGF23, in the plasma of KO mice on calcium-oxalate diet compared to control mice?
4. in the line 358, the subhead “Ecrg4 protects against tubular calcification in males”. Here the conclusion is confusing. It is Ecrg4 KO mice show lower tubular dilation and nephrocalcinosis, means that loss of Ecrg4 has protective effect but not Ecrg4 itself. Same problem occurs in the later discussion section, in line 397 “Second, the mechanism by which Ecrg4 reduces tubular calcifications remains open”. Loss of Ecrg4 reduces injury rather Ecrg4 itself; In line 385-386, “In this system, the decrease of Nfkb1 expression might indicate that crystal-induced inflammation is reduced in presence of Ecrg4”, based on current results that decrease in Nfkb1 expression is in parallel with reduced Ecrg4 mRNA expression, so precisely, the possibly reduced inflammation (no evidence here) is due to decreased Ecrg4 mRNA expression rather “in presence of Ecrg4”. In contrast, the absence of Ecrg4 might have anti-inflammatory effect based on authors’ conclusions in this study, which is consistent with the following discussion that ECRG4 peptide is proinflammatory.

Minor

5. Y axis legend of S13A Fig is wrong, please correct it. It is not Ecrg4 expression.
6. In result section, line 313-314, “without major changes in the expression levels of 314 the ECRG4 downstream genes Nfkb1, Nfkb2, P53 and Cox2”. However, Nfkb1 mRNA expression is significantly reduced on 600 ug crystal and author should make it clear and clarify it because authors used this result in later discussion.
